# C‐terminal deletion‐induced condensation sequesters AID from IgH targets in immunodeficiency

**DOI:** 10.15252/embj.2021109324

**Published:** 2022-04-26

**Authors:** Xia Xie, Tingting Gan, Bing Rao, Weiwei Zhang, Rohit A Panchakshari, Dingpeng Yang, Xiong Ji, Yu Cao, Frederick W Alt, Fei‐Long Meng, Jiazhi Hu

**Affiliations:** ^1^ State Key Laboratory of Molecular Biology CAS Center for Excellence in Molecular Cell Science Shanghai Institute of Biochemistry and Cell Biology Chinese Academy of Sciences University of Chinese Academy of Sciences Shanghai China; ^2^ The MOE Key Laboratory of Cell Proliferation and Differentiation Genome Editing Research Center School of Life Sciences Peking‐Tsinghua Center for Life Sciences Peking University Beijing China; ^3^ Program in Cellular and Molecular Medicine Howard Hughes Medical Institute Boston Children’s Hospital Boston MA USA; ^4^ Department of Genetics Harvard Medical School Boston MA USA; ^5^ Shanghai Institute of Precision Medicine Ninth People’s Hospital Shanghai Jiao Tong University School of Medicine Shanghai China

**Keywords:** activation‐induced cytidine deaminase, class switch recombination, dominant‐negative, immunodeficiency, protein condensation, Chromatin, Transcription & Genomics, DNA Replication, Recombination & Repair, Immunology

## Abstract

In activated B cells, activation‐induced cytidine deaminase (AID) generates programmed DNA lesions required for antibody class switch recombination (CSR), which may also threaten genome integrity. AID dynamically shuttles between cytoplasm and nucleus, and the majority stays in the cytoplasm due to active nuclear export mediated by its C‐terminal peptide. In immunodeficient‐patient cells expressing mutant AID lacking its C‐terminus, a catalytically active AID‐delC protein accumulates in the nucleus but nevertheless fails to support CSR. To resolve this apparent paradox, we dissected the function of AID‐delC proteins in the CSR process and found that they cannot efficiently target antibody genes. We demonstrate that AID‐delC proteins form condensates both *in vivo* and *in vitro*, dependent on its N‐terminus and on a surface arginine‐rich patch. Co‐expression of AID‐delC and wild‐type AID leads to an unbalanced nuclear AID‐delC/AID ratio, with AID‐delC proteins able to trap wild‐type AID in condensates, resulting in a dominant‐negative phenotype that could contribute to immunodeficiency. The co‐condensation model of mutant and wild‐type proteins could be an alternative explanation for the dominant‐negative effect in genetic disorders.

## Graphical abstract

The dominant‐negative effect of immunodeficiency‐associated activation‐induced deaminase variants lacking the C‐terminus can be explained by its trapping of wild‐type AID away from antibody switch regions.

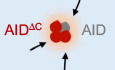

## Introduction

Antigen receptor diversification is one of the hallmarks of adaptive immunity. Lymphocytes employ programmed DNA lesions to initiate diversification processes and generate diverse antigen receptor repertoires (Alt *et al*, 2013). In developing B cells, V(D)J recombination assembles the antigen receptor variable exons to form the primary antibody repertoire. Upon antigen stimulation, naïve B cells experience another two diversification processes, named class switch recombination (CSR) and somatic hypermutation (SHM), to further diversify the antibody repertoire. CSR switches the immunoglobulin (*Ig*) constant gene to change its effector function, while SHM mutates the variable exons to allow antibody affinity maturation (Alt *et al*, 2013). The resulting antigen receptor repertoires help to recognize diverse pathogens including infectious viruses.

Activation‐induced cytidine deaminase (AID), coded by the *AICDA* gene, initiates both CSR and SHM (Muramatsu *et al*, 2000) by directly deaminating the cytidines (C) at *Ig* heavy chain (*IgH*) switch (S) regions or *Ig* variable exons, respectively. Besides the on‐target *Ig* loci, AID frequently miss‐targets a group of off‐target sites associated with intragenic super‐enhancers (Meng *et al*, 2014; Qian *et al*, 2014), leading to B cell malignancies (Casellas *et al*, 2016). Thus, the mutator activity of AID is highly regulated in B cells (Yeap & Meng, 2019). AID protein contains a nuclear localization signal (NLS) sequence at the N‐terminus and a nuclear export signal (NES) sequence at the C‐terminus (Brar *et al*, 2004; Ito *et al*, 2004; McBride *et al*, 2004). Wild‐type AID protein is mostly located in the cytoplasm, while the NES‐mutated AID mutants prefer to accumulate in the nucleus (Ito *et al*, 2004; McBride *et al*, 2004).

Nuclear accumulation of AID mutator protein potentially possesses a great threat to genome integrity. The C‐terminal NES sequence is not required for its deaminase activity *in vitro* (Mu *et al*, 2012; Qiao *et al*, 2017), and ectopically overexpressed NES‐mutated AID protein can efficiently deaminate *E. coli* genomic DNA (Barreto *et al*, 2003; Bransteitter *et al*, 2004), generate substantial mutations in chicken B‐lineage DT40 cells (Barreto *et al*, 2003; Geisberger *et al*, 2009) and mouse fibroblasts (Ta *et al*, 2003). Surprisingly, a group of AID C‐terminal deletion (AID^ΔC^) alleles, which lack the C‐terminal NES, were reported in immunodeficient Hyper‐IgM syndrome type 2 (HIGM2) patients, including R190X, V186X, etc. (Durandy *et al*, 2007). Similarly, in mouse B cells, the nuclear‐retention AID^ΔC^ mutants fail to support CSR in *ex vivo* cytokine‐activated B cells (Barreto *et al*, 2003; Ta *et al*, 2003; Geisberger *et al*, 2009; Zahn *et al*, 2014).

The defective CSR in AID^ΔC^ expression B cells leads to the hypothesis that the AID C‐terminus has an NES‐independent role(s), for example, replacement of the endogenous NES sequence with other NESs can restore the nuclear export but not CSR (Geisberger *et al*, 2009). The C‐terminus could affect protein stability and/or interact with unknown factors (Geisberger *et al*, 2009; Ellyard *et al*, 2011). In this context, C‐terminus‐dependent interactions with polyA RNA and/or hnRNP were proposed (Nonaka *et al*, 2009; Hu *et al*, 2015; Mondal *et al*, 2016). Similarly, AID C‐terminus was proposed to be involved in DNA repair and recombination in CSR, for example, affecting recombinant end pairing (Sabouri *et al*, 2014), recruiting UNG and Msh2 protein to antibody gene (Ranjit *et al*, 2011), or affecting the functions of DNA damage response factors (Zahn *et al*, 2014). How this link between AID and downstream DNA repair factors contributes to physiological CSR is not clear, as CSR is processed in a two‐step manner (Zarrin *et al*, 2007), that is, AID initiates DNA lesions first and the general DNA repair factors finally join the breaks.

Among the deleterious *AICDA* mutations in HIGM2 (Revy *et al*, 2000), most of the HIGM2 patient‐derived *AICDA* mutants are autosomal recessive mutants (Ta *et al*, 2003) and highly correlated to AID protein stability or catalytic activity (Qiao *et al*, 2017). However, AID^ΔC^ alleles were reported to be autosomal dominant mutants (Durandy *et al*, 2007). Dominant mutations inhibit the functions of wild‐type gene products through the formation of nonfunctional self‐assembled oligomers or competition of limited substrates/cofactors (Herskowitz, 1987). AID^ΔC^ mutants cannot be simply grouped into these two categories. In the current study, we find the AID^ΔC^ mutants fail to target antibody genes and frequently infiltrate into nuclear membraneless organelles through their intrinsic sequence features. Wild‐type AID proteins, which dynamically shuttle between nucleus and cytoplasm, are trapped within these subnuclear structures in presence of AID^ΔC^ protein, resulting in the dominant‐negative effect.

## Results

### Guided AID^ΔC^ can support high levels of CSR

Pathogenic mutant AID^R190X^ with truncation of the C‐terminal 9 amino acids (aa) failed to support efficient CSR in CSR‐activated B cells (Appendix Fig S1A) and CH12F3 B cell line (Appendix Fig S1B), consistent with previous reports (Barreto *et al*, 2003; Ta *et al*, 2003; Geisberger *et al*, 2009; Zahn *et al*, 2014). The defective CSR in AID^R190X^‐complemented B cells cannot be attributed to nuclear protein levels (Appendix Fig S1C and D), or its deamination activity (Appendix Fig S1E and F). To examine the *IgH* breakage and rearrangements in activated B cells expressing different AID variants, we applied the high‐throughput genome‐wide translocation sequencing (HTGTS) (Hu *et al*, 2016), which presents both “bait” Sμ DSBs and CSR junctions. We applied HTGTS with the same numbers of B cells and detected marginal levels of CSR junctions from B cells complemented with AID^R190X^ and AID^cry^ with low CSR levels (Appendix Fig S2A). For the bait Sμ DSBs, wild‐type AID generated a great number of broken ends, which can be visualized as spikes at RGYW (R: A/G; Y: T/C; W: A/T) motifs (Appendix Fig S2B). However, no such specific enrichments at RGYW motifs were observed in B cells complemented with AID^ΔC^ mutants (Appendix Fig S2B). Therefore, the catalytic‐active AID^ΔC^ protein induces fewer DNA breaks at the *IgH* locus.

**Figure 1:**
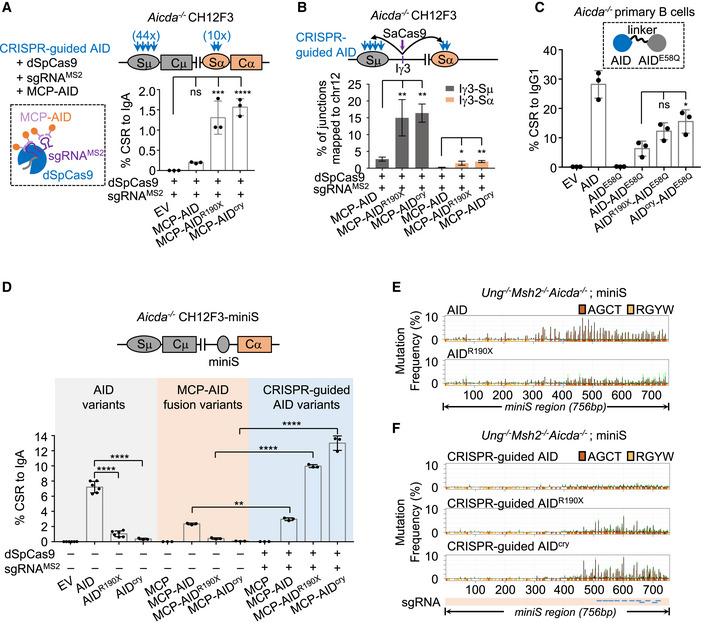
AID C‐terminal tail is required for its targeting to *IgH* substrates CRISPR‐guided AID^ΔC^ protein could support CSR in *Aicda* ^−/−^ CH12F3 cells. Left, CRISPR‐guided AID is schematically illustrated with each component listed. Right, CRISPR‐guided AID‐initiated CSR levels to IgA. Blue arrows represent multiple CRISPR recognition sites. The number in the parentheses indicates CRISPR recognition sites in each S region. Three biological replicates are plotted by dots and mean with standard deviation (SD).CRISPR‐guided AID‐generated breaks at the S region in *Aicda* ^−/−^ CH12F3 cells were quantitatively detected by PEM‐seq. Top, schematic illustration shows the bait *Sa*Cas9 site at Iγ3 region and CRISPR‐guided AID variants targeting sites at S regions. Bottom, percentages of translocation junctions between Iγ3 and Sμ or Sα regions are plotted as mean with SD of three biological replicates in the bar plot.AID‐AID^E58Q^ dimer proteins were expressed in AID‐deficient CSR‐activated B cells, AID dimer is schematically illustrated on top and CSR levels to IgG1 are shown. Three biological replicates are plotted by dots and mean with SD is shown.CSR levels to IgA in *Aicda* ^−/−^ CH12F3‐miniS cells with indicated AID variants or CRISPR‐guided AID variants. Levels of CSR to IgA are plotted as mean with SD in the bar plot (six biological replicates for EV, AID, AID^R190X^, and three biological replicates for other samples).Mutation profiles of miniS region in *Ung* ^−/−^ *Msh2* ^−/−^ *Aicda* ^−/−^ miniS cell lines complemented with indicated AID. Mutation frequency at each nucleotide along the 756‐bp miniS region is plotted as a bar graph with green error bars, representing mean with standard error of the mean (SEM) of three biological replicates. Positions of AID‐preferred AGCT and other RGYW motifs are marked with orange and yellow respectively under each plot.Mutation profiles of miniS region in *Ung* ^−/−^ *Msh2* ^−/−^ *Aicda* ^−/−^ miniS cell lines expressing CRISPR‐guided AID variants. Mutation frequency at each nucleotide is plotted as mean with SEM of three biological replicates in the bar graph with green error bar. Plots are labeled as in (B). The sgRNA targeting position is indicated with blue lines at the bottom. Data information: For panels A, B, and C, one‐way ANOVA followed by Dunnett’s multiple comparisons test was used for significance assessment. Unpaired two‐tailed Student’s *t*‐test was performed for panel D. For all panels, ****P < 0.0001; ***P < 0.001; **P < 0.01; *P < 0.05; ns, *P* > 0.05. Source data are available online for this figure.

To test whether reduced AID recruitment at *IgH* locus or insufficient processing of AID deamination products to breaks leads to the observed phenotypes, we constructed a synthetically guided AID^ΔC^ by two independent approaches and examined the CSR levels in the respective cells. In the first, we fused AID variants with the CRISPR/Cas9 modules (Fig 1A). When the AID^ΔC^ protein was fused to nuclease‐dead *Streptococcus pyogenes* Cas9 (dSpCas9) or MS2 coat protein (MCP), its deamination activity could be directed to a specific genomic locus with a single‐guide RNA (sgRNA) or sgRNA with MS2 hairpins (sgRNA^MS2^) to generate DNA mutations (Ma *et al*, 2016; Liu *et al*, 2018). We thus tested whether guided AID^ΔC^ protein could support CSR in B cells. Given the repetitive nature of S regions, we designed sgRNA targeting Sμ and Sα multiple times (Sμ: 44 times; Sα: 10 times; allowing 1 bp mismatch in a 12 bp‐long seed sequence proximal to the NRG PAM, Appendix Table S2, Appendix Fig S3A). The multiple recognition sites in S regions ensured the efficient Cas9‐initiated CSR when wild‐type SpCas9 was used (Appendix Fig S3A). Therefore, we fused AID or AID^ΔC^ mutants to MCP and introduced different MCP‐AID variants together with the dSpCas9 and sgRNA^MS2^ (a system dubbed CRISPR‐guided AID, Fig 1A) into AID‐deficient CH12F3 cells. Different MCP‐AID variants were expressed at comparable levels, with relatively higher MCP‐AID levels than others (Appendix Fig S3B). The CRISPR‐guided wild‐type AID could induce substantial CSR from IgM to IgA, while higher efficiencies were observed by using AID^cry^ and AID^R190X^ (Fig 1A). To visualize the CRISPR‐guided AID‐generated breaks at S regions, we performed a quantitative version of HTGTS referred to as PEM‐seq (primer‐extension‐mediated sequencing) (Yin *et al*, 2019), which can quantitatively reveal end‐joining initiated by CRISPR/Cas9 tools. We employed *Staphylococcus aureus* Cas9 (SaCas9) and an sgRNA targeting the Iγ3 region to induce “bait” DSBs for preparing PEM‐seq libraries. Substantial translocation junctions were captured between Iγ3 bait and Sμ or Sα in PEM‐seq libraries from CRISPR‐guided AID variants expressing AID‐deficient CH12F3 cells, and the junction numbers at both S regions (Fig 1B) were well‐correlated with the CSR levels (Fig 1A).

In the second approach, AID^ΔC^ was synthetically recruited to endogenous *IgH* locus in B cells through a dimerization method. Among the AID/APOBEC family members, several of them contain two tandem domains, for example, APOBEC3B, APOBEC3F, and APOBEC3G (Harris & Dudley, 2015). A similar dimer configuration has been applied to access the function of AID mutants by fusing AID mutants with the full‐length catalytic‐dead AID^E58Q^ (Methot *et al*, 2018). Thus, we generated a panel of synthetic AID^ΔC^‐AID^E58Q^ dimer proteins (Fig 1C), in which setting the full‐length AID^E58Q^ could recruit AID^ΔC^ to the *IgH* locus. Similar to the CRISPR‐guided AID, the AID^ΔC^‐AID^E58Q^ dimer proteins supported substantial levels of CSR (Fig 1C), though the levels of dimer proteins were lower than those of the monomers (Appendix Fig S3C).

Therefore, with two different approaches to synthetic recruitment, synthetic‐recruited AID^ΔC^ can support substantial levels of CSR *in vivo*.

### Guided AID^ΔC^ can support deamination‐initiated mutagenesis in an S region

To further test the AID targeting in S regions, we generated a CH12F3‐miniS cell line which allows the assessment of the mutation spectrum at an intact mini‐switch region. In the AID‐deficient CH12F3‐miniS cells, the endogenous Sα was replaced with a 756‐bp core Sμ sequence via a recombinase‐mediated cassette exchange approach (Han *et al*, 2011) (Appendix Fig S3D and E). Both wild‐type AID and the MCP‐tagged wild‐type AID could support substantial levels of CSR in the AID‐deficient CH12F3‐miniS cells (Fig 1D, columns 2 and 6 from left), while the latter showed a relatively lower CSR level. AID^ΔC^ failed to support efficient CSR in CH12F3‐miniS cells (Fig 1D, columns 3,4 and 7,8 from left; Appendix Fig S3F). However, in the CRISPR‐guided approach, both AID and AID^ΔC^ supported substantial levels of CSR under the same treatments at comparable protein levels (Fig 1D and Appendix Fig S3G), similar to the findings in the parental CH12F3 cells (Fig 1A). CRISPR‐guided AID^ΔC^ induced a higher CSR level to IgA than guided AID in CH12F3‐miniS cells, reaching more than 10%.

The CH12F3‐miniS cells allow us to sequence through the whole miniS region to check for AID footprints. To trace AID deamination sites, we knocked out *Ung* and *Msh2* in CH12F3‐miniS cells (Fig 1E and Appendix Fig S3E), in which genetic background the *bona fide* AID deamination events are processed during DNA replication and can be visualized by C>T transition mutations (Rada *et al*, 2004). We found that the mutation frequency at the miniS region was greatly decreased in AID^R190X^‐expressing cells compared to that in wild‐type AID‐expressing cells (Fig 1E), indicating a decreased targeting of AID^R190X^. In the same context, CRISPR‐guided AID^ΔC^ efficiently generated C>T transition mutations around the CRISPR recognition sites (Fig 1F), and the mutation frequencies of different AID variants were consistent with the CSR levels (Fig 1D, last three columns from left).

Collectively, the results demonstrate that AID C‐terminus is crucial for its recruitment to the S regions, and the AID^ΔC^ deamination products can be efficiently channeled into recombination events.

### AID^ΔC^ protein has the tendency of condensation in cells

**Figure 2:**
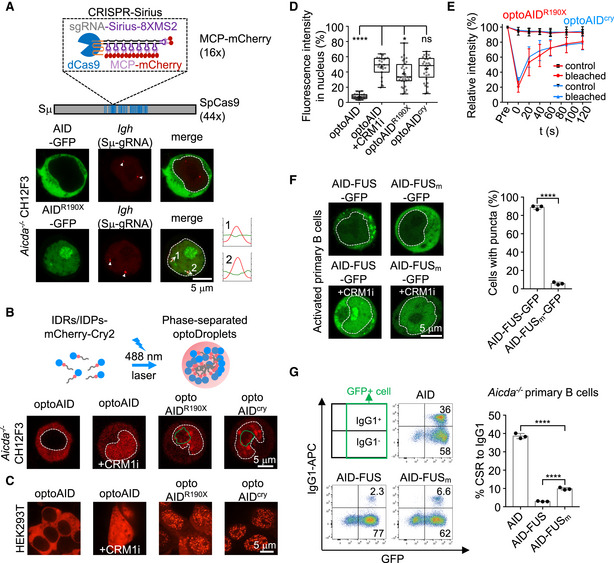
The condensation of AID‐delC reduces its activity in CSR Representative images showing the localization of AID (green), AID^R190X^ (green), and endogenous *IgH* loci (red) in nucleofected *Aicda* ^−/−^ CH12F3 cells. The *IgH* loci are visualized using Sμ‐gRNA and indicated by white arrows. An illustration of CRISPR‐Sirius *IgH* locus visualization is shown on top. Scale bar, 5 µm.OptoDroplet formation in CH12F3 cells. Schematic illustration of light‐induced optoDroplet system is depicted on top. AID variants were tagged to the N‐terminus of the optoDroplet construct and blue light was employed to promote the formation of puncta three times followed by imaging each time. Representative images after the third blue light activation in *Aicda* ^−/−^ CH12F3 cells with the indicated AID variants are shown at the bottom. White dashed line and green line depict the shapes of nucleus and nucleolus based on Hoechst staining or GFP‐FBL, respectively. Scale bar, 5 µm.Representative images showing optoDroplet formation of indicated AID variants in HEK293T cells. CRM1 inhibitor (CRM1i) was used for selective trapping of AID in nuclei. Scale bar, 5 µm.The fluorescence intensity of indicated AID variants in each HEK293T cell nucleus was measured and normalized relative to the fluorescence intensity of the whole cell. Data are shown as box plots. Values between lower quartile and upper quartile are represented by box ranges, a horizontal line within the box represents the median, and whisker extends from the minimum value to the maximum value. “+” indicates the mean level. Each dot indicates one cell, and more than 20 cells represent two biological replicate samples were counted. One‐way ANOVA followed by Dunnett’s multiple comparisons test was performed. ****P < 0.0001; *P < 0.05; ns, *P* > 0.05.Intensity recovery lines for optoAID^ΔC^ droplets in FRAP experiment in HEK293T cells. Bleaching occurs at *t* = 0 s. The background‐subtracted fluorescence intensities are normalized by pre‐bleach values. The mean with SD of 10 pairs of optoAID^R190X^ and 10 pairs of optoAID^cry^ is shown, respectively.Formation of puncta in primary B cells complemented with AID‐FUS‐GFP or AID‐FUSm‐GFP. FUSm contains 13 mutations on either Y or R residue. Left: Representative images showing cellular localization of AID‐FUS‐GFP and AID‐FUS_m_‐GFP with and without CRM1 inhibitor (CRM1i) treatment in *Aicda* ^−/−^ primary B cells. Scale bar, 5 µm. Right: Quantification of the number of cells with condensed puncta (3 biological replicates). Unpaired two‐tailed Student’s *t*‐test was performed. ****P < 0.0001.CSR levels to IgG1 in activated *Aicda* ^−/−^ primary B cells complemented with the indicated protein. Left, representative flow cytometry plots measuring CSR to IgG1. Co‐expressed GFP was used to indicate the infected fractions. Right, the mean with SD of three biological replicates is shown in the bar plot. Unpaired two‐tailed Student’s *t*‐test was performed. ****P < 0.0001. Source data are available online for this figure.

To investigate the mechanism underlying the defective recruitment of AID^ΔC^, we ectopically expressed AID and mutants in CH12F3 cells. Under the current experimental condition, AID expression levels measured by the mRNA quantity were similar to those of the endogenous AID expression in CH12F3 cells stimulated by cytokines (Appendix Fig S4A). AID‐GFP protein was dominantly located in the cytoplasm, and partially retained in the nucleus upon CRM1 inhibitor (CRM1i) treatment (Appendix Fig S4B), as previously reported (Brar *et al*, 2004; McBride *et al*, 2004; Patenaude *et al*, 2009). AID^ΔC^‐GFP proteins, including AID^R190X^‐GFP and AID^cry^‐GFP, were constitutively retained in the nuclei (Appendix Fig S4C), with a relative enrichment in the nucleoli marked by Fibrillarin (FBL) (Appendix Fig S4B). In addition, AID^ΔC^‐GFP puncta were occasionally observed outside the nucleolus (Appendix Fig S4B, arrowhead). When increasing the protein levels by overexpressing GFP‐fused AID variants in the U2OS cells, we found that AID^R190X^ puncta can also co‐localize with nucleolus and other nuclear membraneless organelles, such as Cajal bodies, gems, nuclear speckles, or peri‐nucleolar compartments (Appendix Fig S4D), as previous observations in Hela cells (Hu *et al*, 2013, 2014). To test whether the AID^ΔC^ puncta co‐localize with the *IgH* targets in B cells, we visualized the endogenous *IgH* locus by using the CRISPR‐Sirius system in live CH12F3 cells, in which assay the *IgH* Sμ region was labeled with the multiple‐targeting sgRNA (Fig 2A and Appendix Table S2). The CRISPR‐Sirius assay consistently detected two *IgH* loci in CH12F3 cells, and none co‐localized with AID^ΔC^ puncta (Fig 2A). Therefore, these results indicate that the frequent infiltration of AID^ΔC^ proteins into nuclear compartments might affect their targeting of the physiological *IgH* substrate.

Many subnuclear membraneless organelles are formed through the protein phase separation mechanism. We hypothesized that AID^ΔC^ might infiltrate into the subnuclear membraneless organelles through a similar mechanism. The cellular optoDroplet system (Shin *et al*, 2017) has been widely used to verify the intrinsic potential of a protein to form condensates in mammalian cells. In the system, target protein fused with Cry2 tag rapidly forms puncta after blue light activation if the target protein has the potential to form condensates (Fig 2B). When AID^ΔC^ or wild‐type AID was fused to the light‐inducible optoDroplet cassette, scattered micro‐sized spherical puncta were observed in AID^R190X^ and AID^cry^ samples (optoAID^ΔC^) after blue light activation in the CH12F3 (Fig 2B) and HEK293T (Fig 2C) cells. As the activation time of blue light increased, more puncta formed (Appendix Fig S5A). Conversely, wild‐type AID could not drive the formation of optoDroplet either in the cytoplasm or in the nucleus (Fig 2B and 2C), despite the nuclear protein expression level of wild‐type AID in the presence of CRM1i being comparable to that of AID^ΔC^ in HEK293T cells (Fig 2D). Besides, CRM1i treatment did not affect the formation of optoAID^ΔC^ puncta (Appendix Fig S5B). The optoAID^ΔC^ puncta were sensitive to 1,6‐hexanediol (HEX) which is suggested to interrupt hydrophobic interactions (Appendix Fig S5C). Also, the optoAID^ΔC^ puncta could be quickly recovered from photobleaching (Fig 2E and Appendix Fig S5D), suggesting a dynamic assembling nature. Therefore, AID^ΔC^ but not AID shows the condensation‐prone property in cells.

### Protein condensation reduces the *in vivo* deaminase activity of AID at *IgH* locus

We next sought to examine whether the immunodeficiency in human patients caused by AID^R190X^ and AID^V186X^ is related to protein condensation. For this purpose, we made synthetic AID fusion proteins to force full‐length AID to form condensates and then checked the consequent deaminase activity at the *IgH* locus by the CSR assay. We placed a FUS protein, which has a strong ability to form liquid droplets *in vivo* (Qamar *et al*, 2018), at the 3’ end of AID (Appendix Fig S5E). In CSR‐activated AID‐deficient B cells, we found that AID‐FUS chimera protein formed puncta in the cytoplasm and also in the nuclei when treated with CRM1i to elevate the nuclear protein levels (Fig 2F and Appendix Fig S5F). Whereas the FUS mutant with 13 aa mutations (Qamar *et al*, 2018) abolished the phase transition ability of AID‐FUS_m_ (Fig 2F and Appendix Fig S5E). Comparing the CSR levels, we found that AID‐FUS complement resulted in a relatively lower CSR level than that of cells complemented with AID‐FUS_m_ (Fig 2G and Appendix Fig S5G and H). Of note, AID‐FUS_m_ could not fully complement the CSR as wild‐type AID did (Fig 2G), probably due to decreased AID fusion protein levels (Appendix Fig S5G). The comparison of AID‐FUS and AID‐FUS_m_ suggests that phase separation has a deleterious impact on AID activity at *IgH* locus and thus CSR.

### Nuclear AID^ΔC^ condensation traps wild‐type AID protein

**Figure 3:**
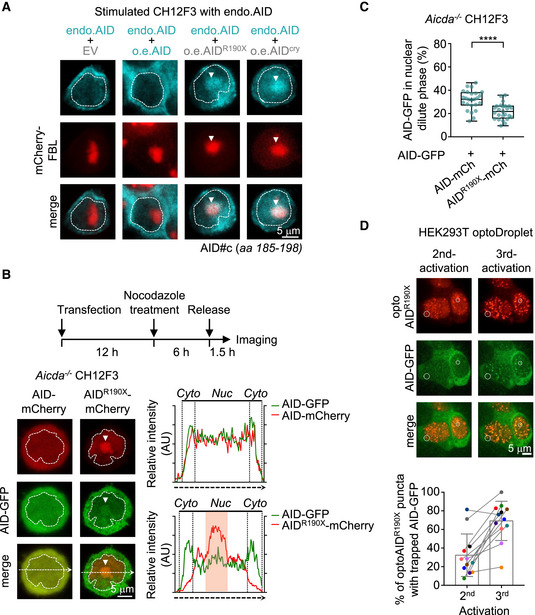
AID^ΔC^ condensate traps wild‐type AID in B cells Representative immunofluorescence images of endogenous AID in the presence of ectopically expressed EV, AID, AID^R190X^, or AID^cry^ in cytokine‐stimulated CH12F3 cells. EV, empty vector control. An antibody against AID C‐terminus (185‐198 aa) detected both endogenous and ectopically expressed full‐length AID. The nucleolus is indicated by co‐nucleofected mCherry‐FBL. The white dashed line depicts the shape of the nucleus based on Hoechst staining. Arrows indicate the location of puncta formed by endogenous AID. The images were taken by the N‐SIM microscope and processed via N‐SIM software. A middle slice of the images was shown. Scale bar, 5 µm.Co‐condensation of AID and AID^R190X^ in the nucleus of CH12F3 cells in the early G1 phase. Top: experimental layout. GFP‐tagged wild‐type AID (green) and mCherry‐tagged AID variants (red) were co‐nucleofected in *Aicda* ^−/−^ CH12F3 cells. The resulting cells were arrested with nocodazole for 12 h after transfection. Six hours later, nocodazole was removed from the system for 1.5 h before imaging. Bottom left: representative images showing subcellular localization of indicated AID variants. The white dashed line depicts the shape of the nucleus based on Hoechst staining. The white arrow indicates the location of the co‐condensed puncta. Scale bar, 5 µm. Bottom right: plot profiles of the images. Cyto: cytoplasm, Nuc: nucleus.Quantification of the relative fluorescence intensity of AID‐GFP in the diluted phase of cells shown in (B). The fluorescence intensity of AID‐GFP in the diluted nuclear phase was normalized by the total intensity from the whole cell. Data are shown as box plots. Values between lower quartile and upper quartile are represented by box ranges, a horizontal line within the box represents the median, and whisker extends from the minimum value to the maximum value. “+” indicates the mean level. Each dot indicates one cell (*n* = 25), and representative results from three biological replicates were shown. Unpaired two‐tailed Student’s *t*‐test was performed. ****P < 0.0001.Dynamics of trapping of wild‐type AID in optoAID^R190X^ condensates. Top, representative fluorescence images at different time points. Dashed circles indicate the co‐condensed puncta formation of AID‐GFP. Scale bar, 5 µm. Bottom, a summary of 12 observed cells is shown as mean ± SD in a bar graph. Percentages of optoAID^R190X^ puncta with trapped wild‐type AID are plotted for the second and third blue light activations, and the colored dot indicates one cell. Source data are available online for this figure.

AID^ΔC^ behaves in a dominant‐negative manner in the heterogeneous AID^ΔC^/AID cells. We sought to test whether the condensation‐prone feature of AID^ΔC^ protein affects the subnuclear location of wild‐type AID. To test that, we expressed different AID variants in AID‐proficient CH12F3 cells and examined the subcellular location of endogenous wild‐type AID with an antibody specifically recognizing the C‐terminal epitope (185–198 aa) of AID. In the experimental design, the detected fluorescent signal came from full‐length AID. In the control cells transfected with an empty vector, most of the endogenous AID protein was detected in the cytoplasm with a diffused distribution pattern (Fig 3A). In the cells transfected with wild‐type AID, the dominant cytoplasmic locations of both endogenous AID and ectopically expressed AID proteins were detected (Fig 3A, the endogenous and ectopic full‐length AID proteins cannot be distinguished). However, in the AID^ΔC^‐transfected cells, a bright spot formed by endogenous AID was detected inside the nucleus (Fig 3A), which co‐localized with nucleolus marked by FBL (Fig 3A). These data suggest that co‐expressed AID^ΔC^ protein can alter the subcellular location of endogenous wild‐type AID protein.

To further examine the “tapping” model, we co‐transfected AID‐deficient CH12F3 cells with mCherry‐tagged AID^R190X^ and GFP‐tagged wild‐type AID and checked their subcellular localization. In the cycling CH12F3 cells, the AID‐GFP was trapped in the nuclear AID^R190X^‐mCherry puncta (Appendix Fig S6A), consistent with our observation with the anti‐AID C‐terminus antibody (Fig 3A). The subcellular location of AID is linked to the cell cycle, and in the early G1 phase, wild‐type AID showed a partially nuclear location (Fig 3B; Wang *et al*, 2017). We synchronized the AID‐GFP expressing CH12F3 cell with nocodazole treatment and examined the AID location in early G1 upon nocodazole release (Appendix Fig S6B and C). In early G1, a significant amount of wild‐type AID protein was trapped in the nuclear AID^ΔC^ condensates (Fig 3B). The nuclear AID‐GFP signal in the diluted phase in cells with AID^ΔC^ condensate was significantly lower than that in cells without AID^ΔC^ condensate (Fig 3C). Similar “trapping” of wild‐type AID in nuclei by AID^R190X^ condensates was also observed in non‐B cells, for example, U2OS cells (Appendix Fig S6D). Thus, although wild‐type AID dynamically shuttles between cytoplasm and nucleus, nuclear AID^ΔC^ can trap wild‐type AID proteins in subnuclear compartments.

Last, to visualize the dynamic “trapping” process, we applied the optoDroplet system, which allows us to tune the formation of optoAID^ΔC^ droplets via light activation. We co‐transfected GFP‐tagged wild‐type AID with optoAID^ΔC^ in HEK293T cells and found that wild‐type AID was specifically trapped in nuclear optoAID^ΔC^ condensates but not optoFUS_N_ condensates (Appendix Fig S6E). In the process of light‐induced droplet formation, we noticed that wild‐type AID was dynamically trapped into preexisting AID^ΔC^ condensates (Fig 3D). Collectively, the data demonstrated a dynamic trap of full‐length AID in nuclear AID^ΔC^ puncta.

### AID^ΔC^ condensation ability is associated with the dominant‐negative effect

**Figure 4:**
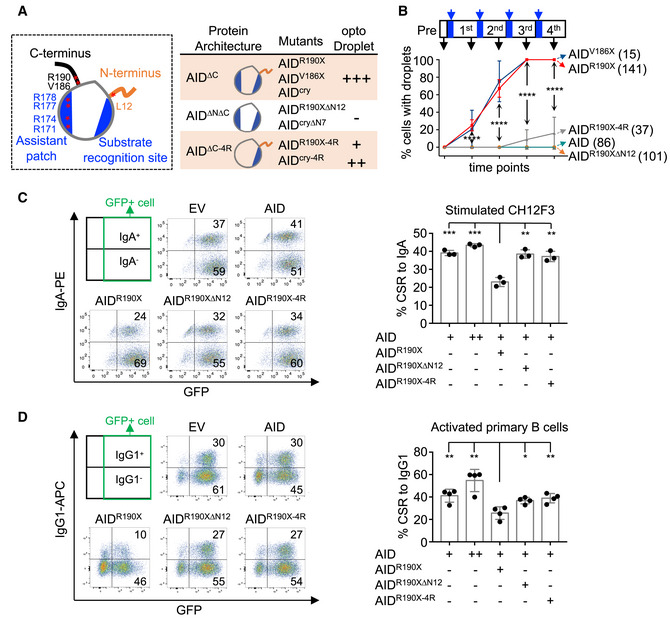
Intrinsic sequence features contribute to AID^ΔC^ condensation and dominant‐negative effect Summary of optoDroplet formation driven by indicated AID mutants. Left, AID domain architecture is illustrated based on the published structure and AlphaFold prediction. The orange and black lines indicate predicted low‐complexity sequence. Asterisks indicate residues for AID mutations. Right, AID mutants are grouped into three categories based on protein architecture and the optoDroplet formation capacity is summarized at right. “+++”: strong capacity with a cutoff of 90% cells showing optoDroplet puncta formation. “++”: a lower capacity with 50–90% of cells showing puncta formation. “+”: capacity with less than 50% of cells showing puncta formation. “‐”: empty vector control level.The optoDroplet formation of indicated AID mutants along with light stimulation. Top, the procedure of optoDroplet assay is illustrated with indicated light stimulation (blue arrow) and image acquisition (black arrow) time points. Bottom, line plots show quantification of cells with optoDroplet formation as mean ± SD at each acquisition time point. The total acquired cell numbers of each genotype are indicated in parentheses. Significance assessment between AID^R190X^ and AID^R190X‐4R^ was shown at each time point. Unpaired two‐tailed Student’s *t*‐test was performed. ****P < 0.0001.CSR levels of ectopically expressed AID variants in AID‐proficient CH12F3 cells. Left, representative flow cytometry plots of CSR to IgA. Co‐expressed GFP indicates the infected cell fractions. Right, the CSR mean with SD of three biological replicates is shown in the bar plot.CSR levels of ectopically expressed AID variants in CSR‐activated B cells. Left, representative flow cytometry plots of CSR to IgG1. Right, the bar plot shows the CSR mean with SD of four biological replicates. Data information: One‐way ANOVA followed by Dunnett’s multiple comparisons test was performed for (C) and (D). ***P < 0.001; **P < 0.01; *P < 0.05. Source data are available online for this figure.

The co‐condensation model offers an explanation for the observed dominant‐negative effect. To establish a causal link between co‐condensation and dominant‐negative effect, we identified and tested the AID variants without the condensation ability in B cells undergoing CSR. First, we generated a panel of AID mutants to dissect key residues involved in AID^ΔC^ phase separation and applied optoDroplet analysis to summarize the condensation property of these mutants (Fig 4A and Appendix Fig S7A). Besides R190X mutation, we found that another *AICDA* mutation, V186X, identified in HIGM syndrome, also led to punctum formation after light activation in the optoDroplet assay (Fig 4A and Appendix Fig S7B), further suggesting that C‐terminal deletion promotes AID condensation. Both the N‐ and C‐terminuses of AID harbor short *in silico*‐predicted intrinsically disordered sequences (Appendix Fig S7A) that might be involved in the formation of protein condensates, which, however, were excluded from structure determination (Qiao *et al*, 2017) and have low per‐residue confidence scores in AlphaFold prediction (Jumper *et al*, 2021). We constructed the mutants with deletion of the N‐terminal 12 aa in AID^R190X^ (AID^R190XΔN12^) or the N‐terminal 7 aa in AID^cry^ (AID^cryΔN7^) and found that both of them could not drive the optoDroplet formation even with four times of light activation (Fig 4B and Appendix Fig S7B and C). Based on the structure of AID protein, we further mutated all four arginines (4R) at the positively charged assistant patch of AID (Fig 4A and Appendix Fig S7A). The 4R mutants (AID^R190X‐4R^ and AID^cry‐4R^) were still able to drive puncta formation, but at a much slower speed when compared to the corresponding proteins with the same times of light activation (Fig 4A and B and Appendix Fig S7C). Consistent with the optoDroplet results, the AID^R190XΔN12^ or AID^R190X‐4R^ protein lost the nucleolar enrichment when expressed in CH12F3 cells (Appendix Fig S8A). It is of note that the N‐terminal deletion and 4R mutation also slightly affected the nuclear localization of AID^R190X^, which could be attributed to the partial deletion of the NLS for the former. How the 4R mutation affects cytoplasm/nuclear trafficking is still not clear. Thus, these results imply that the intrinsic sequence features, including both the flexible N‐terminus and the positively charged surface patch, are involved in AID^ΔC^ protein condensation.

Second, we tested these mutants in CSR assays. In the *ex vivo* CSR assay, the AID^R190XΔN12^ or AID^R190X‐4R^ failed to support CSR (Appendix Fig S8B), as the N‐terminus and the 4R patch are required for AID activity (Abdouni *et al*, 2018). The dominant‐negative effect of AID variants in patients can be rebuilt by overexpression of the AID mutant in the AID‐proficient B cells. Compared to AID‐transfected cells, AID^R190X^‐transfected cells showed less than half the CSR levels in AID‐proficient CH12F3 (Fig 4C) and *ex vivo* CSR‐activated primary B cells (Fig 4D and Appendix Fig S8C and D). A similar dominant‐negative effect was observed in AID^cry^‐expressed B cells (Appendix Fig S8E). To directly study the causal link between co‐condensation and dominant‐negative effect, we introduced AID^R190XΔN12^ or AID^R190X‐4R^ with a decreased ability to form protein condensation (Fig 4B) into the same B cells. We found that these mutants rescued the dominant‐negative effect of AID^R190X^ on CSR levels (Fig 4C and D), though they expressed at a comparable level to AID^R190X^ (Appendix Fig S8D). Collectively, these data suggest that the sequestering of wild‐type AID activity by AID^ΔC^ condensates leads to impairment of CSR, referred to as the long‐standing dominant‐negative effect in human patients.

### AID^ΔC^ underwent condensation *in vitro* in the presence of the crowding agent

**Figure 5:**
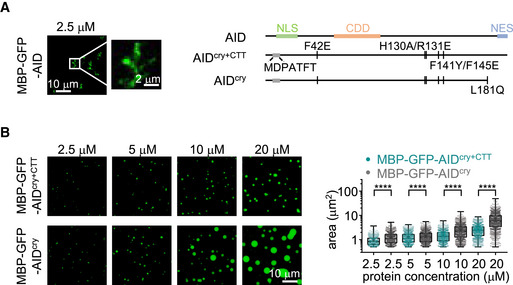
AID^ΔC^ has the tendency to condensation *in vitro* Left, representative images of MBP‐GFP‐AID in the presence of 10% PEG8000. Scale bar, 10 and 2 µm, respectively. Right, the schematic protein sequences.Left, representative images of MBP‐GFP‐AID^cry+CTT^ or MBP‐GFP‐AID^cry^ in the presence 10% PEG8000 at the indicated protein concentrations. Scale bar, 10 µm. Right, sizes of MBP‐GFP‐AID^cry+CTT^ or MBP‐GFP‐AID^cry^ protein condensates are plotted as box plots with three technical replicates. The box represents values between the lower quartile and the upper quartile, a horizontal line within the box represents the median, and whisker extends from the minimum value to the maximum value. All points are listed and mean is indicated by “+”. Unpaired two‐tailed Student’s *t*‐test was performed. ****P < 0.0001. Source data are available online for this figure.

As previously documented by many labs (Bransteitter *et al*, 2003; Chaudhuri *et al*, 2003; Kohli *et al*, 2009), recombinant wild‐type full‐length AID proteins are largely heterogeneous aggregates and tend to nonspecifically bind to single‐strand nucleotides (Bransteitter *et al*, 2004; Larijani *et al*, 2007; Qiao *et al*, 2017). Although a small fraction of recombinant purified wild‐type AID protein is catalytically active (Appendix Fig S1E and F), the purity and quantity of wild‐type AID and AID^R190X^ do not allow their characterization in *in vitro* protein condensation assays. We partially purified MBP‐GFP‐AID protein and found that the protein tended to aggregate. The aggregation was accelerated by adding the crowding agent PEG8000, and the protein formed irregularly shaped aggregates under the microscope (Fig 5A).

Therefore, we used the MBP‐GFP‐AID^cry^ instead for the *in vitro* protein condensation assay (Appendix Fig S9A and B). The chimeric protein did not show aggregation or condensation at a concentration up to 20 μM. However, in the presence of 10% PEG‐8000, the MBP‐GFP‐AID^cry^ but not the MBP‐GFP solution turned opaque (Appendix Fig S9C). The MBP‐GFP‐AID^cry^ protein formed micro‐sized spherical droplets under the fluorescence microscope (Appendix Fig S9D). After removing the MBP tag, AID^cry^ could form spherical droplets with relatively smaller sizes (Appendix Fig S9E and F). The sizes of MBP‐GFP‐AID^cry^ droplets were positively correlated with protein concentration (Appendix Fig S9G). The droplets could fuse in a liquid‐like fashion in close proximity (Appendix Fig S9H). High salt can affect electrostatic interactions and is frequently used to study the biophysical properties of protein condensation (Sabari *et al*, 2018). We found that AID^cry^ condensation was sensitive to the high concentration of NaCl *in vitro* (Appendix Fig S9I), which is correlated with the role of the surface positive‐charged patch in AID^ΔC^ condensation. These results further confirm that AID^ΔC^ protein also has the tendency of condensation *in vitro*. To further test the role of the AID C‐terminal tail (CTT) in protein condensation *in vitro*, we put the CTT back to AID^cry^ and purified the corresponding protein (AID^cry+CTT^) to homogeneous levels (Appendix Fig S9J). We found that the CTT greatly diminished the condensation of AID^ΔC^ proteins *in vitro* (Fig 5B). Thus, the C‐terminal truncated AID chimeric protein is more prone to condensation *in vitro* in the presence of the crowding agent, suggesting that AID^ΔC^ protein may not condensate by itself.

### An unbalanced molecular ratio of AID^ΔC^/AID in the nucleus facilitates co‐condensation

**Figure 6:**
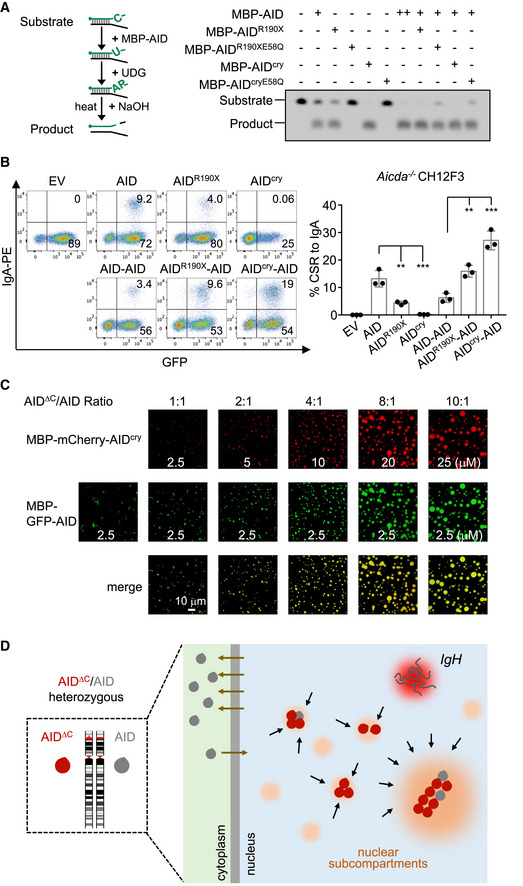
Unbalanced molecular ratio of AID^ΔC^/AID facilitates the dominant‐negative effect AID deamination assay was performed *in vitro*. Left, a schematic diagram of deamination activity assay. Right, the deamination reaction with indicated protein combinations was processed and visualized. “+”: 0.25 μM of indicated protein; “++”: 0.5 μM of indicated protein; “‐”: not added.CSR to IgA induced by AID variants in CH12F3 cells. Left, representative flow cytometry plots. Right, the CSR mean with SD of three biological replicates is shown in the bar plot. One‐way ANOVA followed by Dunnett’s multiple comparisons test was performed. ***P < 0.001; **P < 0.01.Representative images of the co‐condensation of MBP‐mCherry‐AID^cry^ and MBP‐GFP‐AID. The protein concentrations and molecular ratios are indicated. Scale bar, 10 µm.A working model of AID^ΔC^ dominant‐negative effect. In cells with one C‐terminal deletion *AICDA* allele, large amounts of pathological AID^ΔC^ protein accumulate in the nucleus and are frequently enriched in nuclear subcompartments through its condensation tendency, thereby blocking its proper targeting to the physiological *IgH* substrates. While the wild‐type AID dynamically shuttles between cytoplasm and nucleus, the high nuclear AID^ΔC^:AID ratio causes the trapping of wild‐type AID into AID^ΔC^‐associated condensates, manifesting a dominant‐negative effect. Source data are available online for this figure.

To examine the possibility that the trap of AID protein with AID^ΔC^ leads to an inactive oligomer as elaborated in many other dominant‐negative diseases, we checked the AID^ΔC^ and AID complex activity *in vitro* and *in vivo*. First, we examined the catalytic activity of AID in the presence of equal amounts of different AID^ΔC^ proteins *in vitro*. The AID mixture retained robust deamination activity in the presence of either active or catalytic‐dead AID^ΔC^ proteins (Fig 6A). Second, synthetic AID^ΔC^‐AID dimer proteins were functionally active to support CSR *ex vivo* and induced higher CSR levels (Fig 6B and Appendix Fig S10A). Ectopically overexpressed AID^ΔC^ protein is genotoxic (Zahn *et al*, 2014), leading to cell death. However, genotoxicity cannot fully account for the dominant‐negative effect, as more toxic APOBEC3A overexpression failed to affect CSR (Appendix Fig S10B).

In the AID^ΔC^/AID heterogeneous cells, the estimated nuclear molecular ratio was around 7:1 based on measurements of the relative fluorescence intensity of GFP‐tagged AID^ΔC^ or AID in the nucleus of B cells (Appendix Fig S4A and B). To test the molecular ratio effect, we reconstituted the co‐condensation of wild‐type AID and AID^ΔC^ *in vitro*. The purified MBP‐mCherry‐AID^cry^ and partially purified MBP‐GFP‐AID proteins were mixed with different molecular ratios in the *in vitro* condensation assay. With an increased AID^ΔC^/AID ratio (≥4:1), MBP‐GFP‐AID formed co‐condensates with MBP‐mCherry‐AID^cry^ protein *in vitro* (Fig 6C). Next, we tested whether full‐length AID could dynamically infiltrate into AID^ΔC^ droplets. In the preexisting MBP‐mCherry‐AID^cry^ condensates, MBP‐GFP‐AID gradually infiltrated into the droplets (Appendix Fig S10C), implying that MBP‐GFP‐AID could be dynamically captured by the MBP‐mCherry‐AID^cry^ condensates as revealed *in vivo* (Fig 3). Thus, the co‐trapping of full‐length AID with AID^ΔC^ protein can be reconstituted *in vitro* at an unbalanced molecular ratio as in nuclei.

## Discussion

AID activity is tightly controlled at multiple layers (Yeap & Meng, 2019), including the active nuclear export mediated by its C‐terminal NES, to prevent its promiscuous activity in lymphomagenesis (Casellas *et al*, 2016). Here, we uncover a molecular mechanism underlying the dominant‐negative immunodeficiency of a group of AID C‐terminal deletion mutants in HIGM2 (Appendix Table S3). AID^ΔC^ protein has the potential to form condensates through its intrinsic sequence features. Although nuclear AID^ΔC^ protein unlikely forms a compartment by itself, it is frequently enriched in other nuclear membraneless organelles. Thus, the AID^ΔC^ “traps” the wild‐type AID protein and both are separated from their physiological CSR substrates at the *IgH* locus (Fig 6D). The condensation of AID^ΔC^ explains several bewildered observations, including that the (hyper‐) active nuclear AID variants cannot support CSR (Barreto *et al*, 2003; Ta *et al*, 2003; Geisberger *et al*, 2009; Zahn *et al*, 2014), the subnuclear location of overexpressed AID^ΔC^ protein (Hu *et al*, 2013, 2014), and the hyper‐IgM symptoms of AID^ΔC^ heterozygous patients (Durandy *et al*, 2007). As suggested by the property of phase separation, there is an equilibrium between the high‐concentration and low‐concentration phases (Case *et al*, 2019), and a small amount of AID^ΔC^ may still escape and cause genomic damage, which leads to the genotoxicity of AID^ΔC^ (Zahn *et al*, 2014). CSR requires extensive DSBs in the S regions, and the function of AID is dose dependent (Takizawa *et al*, 2008). Therefore, the escaped amount of AID^ΔC^ or AID cannot efficiently support CSR but may be sufficient to cause mutations at various off‐target sites to induce genotoxicity.

Several lines of evidence support the proposed co‐condensation model. When compared to the gene expression level in CSR‐activated primary B cells, *Aicda* bulk expression is 4‐fold higher in cytokine‐stimulated CH12F3 cells and 3‐fold lower in mouse splenic germinal center (GC) B cells (Appendix Fig S4A). At the single‐cell level, we found AID was heterogeneously expressed in splenic GC B cells *in vivo* (Appendix Fig S10D). The AID^ΔC^ in high‐AID‐expressing GC B cells can reach a level to support condensation and relative enrichment in nuclear compartments. Biochemical assays with recombinant AID proteins together with *in vivo* assays reveal the intrinsic features contributing to protein condensation. In this context, we have established a causal link between the condensation ability and the dominant‐negative effect. Furthermore, the co‐condensation can be recapitulated *in vitro* with recombinant proteins and *in vivo* with optoDroplet assays. In addition, the *in vivo* and *in vitro* features of AID^ΔC^ condensation also suggest that the protein is unlikely to condensate by itself. Thus, cofactor(s), DNA substrates, and/or nascent RNA can further shape the AID phase separation mode by regulating or driving AID^ΔC^ condensation. In this context, the AID cofactors bridging AID^ΔC^ condensation to the nuclear compartments need to be identified and characterized in the future. Alternatives can also be possible to explain parts of the observations. For example, a potential nucleolar factor may recruit the AID mutants to specific compartments through simple protein–protein interaction. The identification of such a factor and the underlying mechanism would be of great interest to the studies of AID‐initiated antibody diversification and/or AID‐related tumorigenesis.

Although the current work focuses on a group of pathogenic AID^ΔC^ mutations, it also suggests that phase separation may regulate the activity of wild‐type AID in physiological conditions. One possibility is that masking the AID N‐/C‐terminus or surface positively charged patches by cofactor(s) changes the physical property of AID protein and facilitates its specific targeting. In this context, the pathological AID^ΔC^ condensation model does not contradict the previous reports on the physiological roles of these key amino acids (Qiao *et al*, 2017; Methot *et al*, 2018). Besides antigen receptor loci, AID is capable of targeting hundreds of genome‐wide convergently transcribed super‐enhancer hubs (Meng *et al*, 2014; Qian *et al*, 2014), which leads to tumorigenesis (Casellas *et al*, 2016). It is tempting to speculate that the transcription‐associated condensation could regulate AID activity in a physiological condition, as AID targets the pausing sites of RNA Pol II through interaction with Spt5 (Pavri *et al*, 2010), and the clustered surface arginine residues are crucial for its association with transcription elongation machinery (Methot *et al*, 2018). The mechanism(s) by which endogenous AID activity is distributed in different chromatin compartments and regulated by nucleic acids and cofactors under protein condensation represent promising future lines of research. The AID^ΔC^ condensation revealed in this study could represent an extreme case in a pathogenic setting and is distinct from any mechanism(s) of wild‐type AID regulation.

The dominant‐negative effect of AID^ΔC^ could be an exemplar of the role of protein condensation in dominant‐negative diseases beyond the known mechanisms (Herskowitz, 1987). We mapped the multivalent interactions crucial for AID^ΔC^ condensation and found the corresponding mutants no longer have the dominant‐negative effect in functional CSR assays. A higher molecular ratio of nuclear AID^ΔC^:AID facilitates the sequestering of wild‐type AID from the *IgH* locus. Therefore, the co‐expression of AID^ΔC^ and AID manifested immunodeficiency. Here we also showed that the AID^ΔC^ and AID do not form a specific inactive complex. Instead, they retain robust activity and form condensates through multivalent interactions to limit their targeting to the *IgH* locus. The observation of AID^ΔC^/AID condensation is distinguished from the protein aggregation, as the term protein aggregation is more frequently used to describe a non‐native state of misfolded protein assembly mediated by irreversible interaction (Alberti & Hyman, 2021), which is exemplified by the AID aggregation (Fig 5A). Distinct forms of condensation have been reported for many physiological processes (Alberti & Hyman, 2021), while the AID^ΔC^/AID condensation belongs to the group of dysfunctional/pathological condensation. The biochemical and functional features of these different condensations need to be fully elaborated in the future. The co‐condensation dominant‐negative model is an important complement to the classical “nonfunctional self‐assembled oligomers” model. Whether a similar mechanism underlies the dominant‐negative effect in other genetic disorders needs further extensive investigation.

## Materials and Methods

### Mouse

*Aicda* ^−/−^ C57BL/6 mouse line was a kind gift from Dr. Tasuku Honjo (Kyoto U). Both male and female mice were used in this study. Mice were maintained in a pathogen‐free animal facility under standard conditions. All animal experiments were performed under protocols approved by the Institutional Animal Care and Use Committee of Shanghai Institute of Biochemistry and Cell Biology.

### Cell lines

The B‐lineage CH12F3 cell line was a kind gift from Dr. Tasuku Honjo (Kyoto U); CH12F3‐derived *Aicda* ^−/−^ and Sα‐RMCE (Han *et al*, 2011) lines are kind gifts from Dr. Kefei Yu (Michigan State U). *Ung*, *Msh2,* and *Aicda* were deleted with CRISPR/Cas9 and confirmed with genomic DNA genotyping‐PCR and RT‐PCR/western blot. A core Sμ region (Yeap *et al*, 2015) was cloned into an RMCE vector described in Han *et al* (2011), and a cell line with core‐Sμ‐replaced Sα is named as miniS line. These cell lines were monitored for mycoplasma contamination regularly. CH12F3 cell lines were cultured in RPMI1640 plus 10% fetal bovine serum (FBS, ExCell Bio, catalog # FSP500, Lot No.11F364). U2OS and HEK293T were from the National Infrastructure of Cell Line, SIBCB, with identifier SCSP‐5030 and SCSP‐502, and cultured in DMEM medium plus 10% FBS (ExCell Bio, catalog # FSP500, Lot No.11F364). Expi293F cells (Thermo Fisher, A14527) were cultured in a chemically defined Union‐293 medium (Union‐Biotech, UP0050). Sf9 cells (Expression Systems, 94‐001) were cultured in protein‐free ESF 921 Insect Cell Culture Medium (Expression Systems, 96‐001) at 27°C.

### Plasmids, oligos, and reference sequence

Plasmids used in this study are summarized in Appendix Table S1. OptoDroplet system was a kind gift from Dr. Clifford Brangwynne (Princeton) with Addgene ID 101221 and 101223. To generate AID‐AID dimer proteins, an AID gene and a codon‐optimized AID gene were tandemly assembled with a 3x(SGGGG) linker. PCR primers and sgRNAs are listed in Appendix Table S2. MiniS region sequence is showed in Appendix Table S2.

### Antibodies

Antibodies for western blot: AID#c (A16217; Abclonal; RRID: AB_2763671; epitope: a.a.185‐198, EVDDLRDAFRMLGF), AID#n (epitope: a.a.7‐24, KQKKFLYHFKNVRWAKGR; rabbit polyclonal antibody generated in this study, Abclonal), GFP (598; MBL; RRID: AB_591819), β‐tubulin (ac008; Abclonal; RRID: AB_2773006), MCP (ABE76; Millipore; RRID: AB_2827507), Flag (F1804; Sigma; RRID: AB_262044), PARP1 (9542S; CST; RRID: AB_2160739), Msh2 (ab227941; Abcam), HA (3724; CST; RRID: AB_10693385). Antibodies for flow cytometry: FITC‐conjugated anti‐mouse IgA (11‐4204‐83; ebioscience; RRID: AB_465222), PE‐conjugated anti‐mouse IgA (12‐4204‐83; ebioscience AB_465918), APC‐conjugated anti‐mouse IgM (1020‐11S; Southern biotech), APC‐conjugated anti‐mouse IgG1 (560089; BD bioscience; RRID: AB_1645625), and PE‐conjugated anti‐mouse IgG1 (550083; BD bioscience; RRID: AB_393553). Biotinylated antibodies for cell purification: CD43 (553‐269; clone S7; BD bioscience; RRID:2255226), IgD (13‐5993‐85; ebioscience; RRID: AB_466861), and CD11c (13‐0114‐85; ebioscience; RRID: AB_466364).

### CSR assay

CH12F3 cell lines were stimulated with anti‐CD40 (16‐0402‐86; ebioscience; RRID: AB_468947), IL4 (CK15; Novoprotein) plus TGF‐β (CA59; Novoprotein). CSR to IgA was monitored at the indicated time. Splenic naïve B cells were purified as previously described (Qiao *et al*, 2017) and cultured in RPMI1640 plus 15% fetal bovine serum (FBS, ExCell Bio, catalog # FSP500, Lot No.11H235). LPS (sigma; L2630‐100MG) and IL4 (CK15; Novoprotein) were used to activate splenic naïve B cells. Infected with retrovirus on Day 1; CSR to IgG1 was monitored at Day 3 and Day 4. For retroviral AID expression, the *Aicda* gene and its mutants were cloned into a pMX‐IRES‐GFP or pMX‐IRES‐Puro vector (Basu *et al*, 2008). The plasmid was co‐transfected with a packaging plasmid pCL‐10A1 (Novus Biologicals) into HEK293T cells using Ca_3_(PO_4_)_2_ co‐precipitation approach. Cell proliferation was monitored by cell counting with a hemocytometer. The cell viability of retroviral vector‐infected B cells was calculated by the fraction of GFP^+^ cells in the whole population.

### Deamination activity

To purify AID, AID^R190X^, AID^R190XE58Q^, AID^cry^ , and AID^cryE58Q^ for assaying deaminase activity, we put their coding sequences into a modified pcDNA3.4 vector, which contains a PreScission protease cleavage site and an N‐terminal His‐ and MBP tag. Expi293F cells (Thermo Fisher, A14527) were cultured in a chemically defined Union‐293 medium (Union‐Biotech, UP0050) at 37°C, supplied with 5% CO_2_. When cell density reached 2.0 × 10^6^ cells per mL, the cells were transiently transfected with the expression plasmids and PEI MAX (Polysciences). Approximately, 0.5 mg of expression plasmids were pre‐mixed with 1.5 mg PEI MAX in 25 ml fresh medium and incubated for 20 min before transfection. The 25‐ml mixture was then added to 500 ml of cell culture and transfected cells were cultured for about 60 h before collection. Cells were harvested by centrifugation at 2,000 *g* and resuspended in lysis buffer (20 mM HEPES pH 7.5, 150 mM NaCl, 10% Glycerol, 1 mM Tris (2‐carboxyethyl) phosphine (TCEP), 5 mM MgCl_2_, 0.2 mg/ml DNase, and 1mM PMSF, 1× protease inhibitor cocktail (MCE)). Cells were lysed by sonication (2× 60 pulses) on ice, followed by centrifugation at 45,000 *g* for 45 min at 4°C. After centrifugation, the supernatant was loaded onto amylose resin (NEB, E8021S) pre‐equilibrated with Buffer A (20 mM HEPES pH 7.5, 150 mM NaCl, 10% Glycerol, 1 mM TCEP). Proteins were eluted with 4 column volumes of Buffer E (Buffer A + 10 mM maltose), after washing with 20 column volumes of Buffer A.

To generate branched DNA substrate, oligos were dissolved in a buffer containing 20 mM HEPES pH 7.5, 150 mM NaCl, and 1 mM TCEP. The two primers were mixed at a 1:1 molar ratio, and the mixture was incubated at 95°C for 5 min and slowly cooled down to room temperature to yield a branched substrate. Each reaction contained 0.5 μM FAM‐labeled substrate in 10 μl reaction buffer with 20 mM HEPES at PH 7.5, 150 mM NaCl, 1 mM TCEP, and a serial titration of MBP‐tagged AID protein ranging from 0.04 to 2.5 μM. After incubation at 37°C for 100 min, samples were denatured at 95°C for 10 min and quenched on ice. Two units of UDG (New England Biolabs, 5 U/μl) were added to each sample. The uracil hydrolyzing reaction was performed at 37°C for 1 h, which products were further processed by adding NaOH to a final concentration of 150 mM and heated at 95°C for 15 min. Urea gel was used to separate the product from the substrate. Images were collected on Image Scanner FLA‐9000 (Fujifilm). ImageJ software package was used for quantification.

### CRISPR‐guided AID targeting

CH12F3 cells were transfected with CRISPR plasmids (Liu *et al*, 2018) by using an NEPA21 Electroporator (NEPA GENE) at 150 V for 5 ms. In each transfection, 10 μg of a plasmid‐coding sgRNA^MS2^‐dSpCas9 and 10 μg of another plasmid‐coding MCP‐fusion AID variants were co‐transfected into 0.5 million of CH12F3 cells in 100 μl Opti‐MEM I Reduced Serum Medium (Gibco, #31985070). Transfected cells were then cultured in 2 ml RPMI1640 medium plus 10% FBS. Three days after transfection, CSR to IgA was monitored and the genomic DNA of the cells was extracted for further studies.

### HTGTS and PEM‐Seq

HTGTS was performed as previously reported (Hu *et al*, 2016). S region rearrangements were cloned from endogenous AID‐initiated Sμ breaks with 5’‐RED‐Iμ primer (Dong *et al*, 2015). The HTGTS cloning primers are listed in Appendix Table S2. The switch junctions were calculated and plotted as previously described (Dong *et al*, 2015). To examine the un‐joined broken ends, the “bait‐only” reads were retrieved from the data, and frequency of breaks at each position was plotted along the bait sequence. The relative frequency at each nucleotide position downstream the bait primer site was indicated by using the ratio of break frequency in AID‐complemented and that in an empty vector complemented sample. To monitor CRISPR‐guided AID‐generated S region breaks, a bait site cleaved by SaCas9 at Iγ3 was used (Appendix Table S2). PEM‐Seq libraries were performed as previously described (Yin *et al*, 2019; Liu *et al*, 2022) to quantitatively recover the Cas9‐generated translocations. In brief, primer extension was performed with biotinylated primers as listed in Appendix Table S2. Enriched biotinylated PCR products were ligated to the bridge adapters, and two subsequent PCR reactions were performed to amplify products for sequencing. Sequences were mapped to mm10 reference via the SuperQ pipeline (Yin *et al*, 2019; Liu *et al*, 2022). In the PEM‐Seq assay, a random barcode was introduced to remove PCR repeats and quantify translocation junctions. The junctions that fall into different S regions were calculated and normalized to the total junctions that fall into Chr.12.

### MiniS somatic mutation assay

MiniS region was PCR amplified with primers listed in Appendix Table S2. PCR was started with 2 μg genomic DNA in four separate reactions. The PCR product was further gel‐purified and fragmentated with an S220 High‐Performance Ultra‐Sonicator (Covaris). Fragments from 50 to 150 bp were harvested and subjected to library preparation with NEBNext Ultra II DNA Library Prep Kit. For a typical miniS Amplicon‐Seq library, about 200–300 k PE150 reads were retrieved and subjected to a previously published SHM analysis pipeline (Yeap *et al*, 2015). At each nucleotide site, an average of 20,000X coverage was usually achieved. Mutation frequency was first normalized to biological controls, for example, either the cells infected with an empty viral vector for retroviral AID expression experiments or non‐transfection control in the CRISPR‐guided AID experiments. Mutation frequency at each nucleotide was plotted along the DNA sequence with highlighted AID‐preferred motifs.

### Live‐cell imaging

*Aicda* ^−/−^ CH12F3 cells were transfected with 10 μg plasmids via the NEPA21 Electroporator (NEPA GENE) at 150 V for 5 ms. Images were taken 24 h after transfection. Cytokine‐stimulated primary B cells were infected with retrovirus and images were taken 48 h after infection. U2OS cells were incubated on 35 mm glass‐bottom plates (Cellvis, D35‐20‐1‐N). Cells were transfected by X‐tremeGENE™ HP DNA transfection reagent (Sigma, 6366244001) following the manufacturer’s instruction with 0.8 μg plasmids at ~ 50% confluency. To label the endogenous *IgH* locus, sgRNA targeting the repetitive Sμ sequence (Appendix Table S2) was cloned into the CRISPR‐Sirius system (Ma *et al*, 2018). The dCas9 and MCP‐mCherry were stable‐expressed in *Aicda* ^−/−^ CH12F3 cells by lentiviral infection, and the sgRNA^MS2^ was transiently transfected into the dCas9 *Aicda* ^−/−^ CH12F3 cells.

The culture medium was replaced with the medium containing 10 μg/ml Hoechst33342 for 10 min in a CO_2_ incubator and then the cells were washed twice with PBS before imaging. All live‐cell imaging experiments were performed using an LSM880 confocal microscope (Zeiss, Thornwood, NY) equipped with an incubation device (5% CO_2_, 37°C).

Quantification was performed using the ImageJ software package (v1.53). In brief, the fluorescence intensity in the whole‐cell region and the nucleus, which was depicted based on Hoechst staining, was measured and background‐subtracted for every single cell. Then the ratio of fluorescence in the nucleus was calculated by normalizing to the fluorescence in the whole cell.

### OptoDroplet assay and FRAP

The optoDroplet assay was adapted from Shin *et al* (Shin *et al*, 2017). HEK293T cells were plated on 35 mm glass‐bottom dishes with 2 mL DMEM medium plus 10% FBS and transfected with Ca_3_(PO_4_)_2_ co‐precipitation approach with 1 μg optoDroplet plasmids. Pre‐activation images were taken 18 h after transfection. OptoDroplet formation was induced with a 488 nm laser for three 1‐second pulses. Images of mCherry (stimulated with 561 nm laser) were taken at 5, 15, and 60 s after each light activation. Image of Hoechst33342 (stimulated with 405 nm laser) was taken at the 60 s time point. Quantification was performed using the ImageJ software package (v1.53).

FRAP assay was performed with optoDroplet systems assembled as abovementioned. OptoDroplet spots (~ 1.6 μm in diameter) were bleached with a 488 nm laser and images were taken every 5 s in a 2.5‐min duration. ImageJ software package was used to quantify the fluorescence intensity. Briefly, background intensity was first subtracted and the relative intensity of the bleaching area at different time points after bleaching was measured and normalized to pre‐bleaching intensity.

HEX treatment was performed with optoDroplets assembled as abovementioned and 1,6‐hexanediol (HEX) (sigma, 240117‐50G) was added into the medium at a final concentration of 5%. Images were taken every 5 s afterward.

CRM1 inhibitor Verdinexor (ApexBio, B4889) was dissolved in DMSO and was used to treat cells 6 h after transfection at a final concentration of 70 nM. OptoDroplet assembling and imaging were performed 18 h after treatment.

### OptoDroplet in the presence of wild‐type AID

Cells were plated at a 35 mm glass‐bottom plate overnight and transfected via Ca_3_(PO_4_)_2_ co‐precipitation approach with 1 μg optoDroplet plasmids plus 0.6 μg AID‐GFP plasmid (1:1 mole ratio) for 18 h. OptoDroplets were assembled as abovementioned.

### OptoDroplet in CH12F3 cells

*Aicda* ^−/−^ CH12F3 cells were transfected with optoDroplet plasmids by using NEPA21 Electroporator at 150 V for 5 ms. Each reaction contains 0.5 million cells, 8 μg pCMV‐optoDroplet plasmid plus 2 μg GFP‐FBL plasmid. OptoDroplets were assembled 24 h after transfection as abovementioned.

### Immunofluorescence and N‐SIM

CH12F3 cells were cultured in a 6‐well plate at 0.5 million/mL overnight under the stimulation of anti‐CD40, IL4, and TGF‐β. Cytokine‐stimulated CH12F3 cells were nucleofected with 1 μg AID plasmid and 2 μg FBL‐mCherry plasmid per million cells following the instructions of Lonza nucleofection kits (Lonza #V4XC‐2012). Then the cells were fixed with 4% formaldehyde for 15 min at room temperature. PBS with 0.5% Triton X‐100 was used for permeabilization after triple PBS wash for about 15 min. Cells were then blocked with PBS with 3% BSA for 45 min at room temperature, followed by a primary antibody of AID#c (A16217; Abclonal; epitope: *185–198 aa*) incubation for 2 h. After a 4‐step wash with the PBS and 0.2% Tween‐20, the cells were incubated with the secondary antibody Alxa Fluor™ 647 goat anti‐rabbit IgG (Invitrogen, #1959073) for 1 h. Then the cells were washed and mounted to a microscope slide for microscopy analysis.

Cells were imaged with the Nikon N‐SIM microscopy at a setting as: pixel—0.12 μm, laser—640 nm, intensity—65%, time resolution—100 ms per frame; laser—561 nm, intensity—70%, time resolution—60 ms; the laser—405 nm, intensity—40%, time resolution—30 ms. The images were taken through the thin slice scan technology. The N‐SIM software package was used to process multiple images.

### Cell cycle synchronization and cell imaging

*Aicda* ^−/−^ CH12F3 cells were transfected by using the NEPA21 Electroporator at a setting of 150 V for 5 ms. Nocodazole was added 12 h after transfection for cell synchronization at a final concentration of 100 ng/ml. After 6 h of nocodazole treatment, cells were washed with PBS and released in the nocodazole‐free medium for 0‐1.5 h. The culture medium was then replaced with the medium containing Hoechst33342 (10 μg/ml) for 10 min. After staining, the cells were washed with PBS and fixed with 4% formaldehyde for 20 min at room temperature. The fixed cells were then used for imaging and flow cytometry analysis. Image capture was performed using an LSM880 confocal microscope (Zeiss). ImageJ software was used for measuring fluorescence intensity. The cell cycle stages (nocodazole‐arrested or nocodazole‐released) were analyzed based on Hoechst staining by using CytoFlex LX flow cytometer (Beckman).

### Protein condensation *in vitro* assay

For protein expression, AID^cry^, EGFP, EGFP‐AID^cry^, EGFP‐AID^cry+CTT^ were cloned into a pFastBac‐Dual vector, which contains an N‐terminal His‐MBP tag. The constructs were transformed into DH10Bac *E. coli* cells for bacmid preparation, and the resulting bacmids were isolated from 5 ml overnight cultures by the magnetic separation method. Baculoviruses were prepared according to the manufacturer’s manual (Invitrogen, Bac‐to‐Bac, cat #10359016). After 72 h infection with baculoviruses, the overexpressed Sf9 cells were harvested and resuspended in lysis buffer (20 mM HEPES pH 7.5, 150 mM NaCl, 10% Glycerol, 1 mM Tris(2‐carboxyethyl) phosphine (TCEP), 5 mM MgCl_2_, 0.2 mg/ml DNase, and 1 mM PMSF, 1x protease inhibitor cocktail). Cells were lysed by sonication on ice and centrifuged at 45,000 *g* for 45 min at 4°C. The supernatant was loaded onto TALON metal affinity resin (Clontech) pre‐equilibrated with Buffer A (20 mM HEPES pH 7.5, 150 mM NaCl, 10% Glycerol, 1 mM TCEP). After a two‐step wash with Buffer A and Buffer W (Buffer A + 20 mM imidazole), proteins were eluted with Buffer E (Buffer A + 150 mM imidazole). The elute was further purified by size‐exclusion chromatography on a Superdex 200 column in buffer containing 20 mM HEPES pH 7.5, 150 mM NaCl, and 1 mM TCEP, and the fractions from the monomer peak were pooled and used in further condensate formation assay. To remove the MBP tag of MBP‐AID^cry^ (with PreScission protease cleavage site in the linker region), PreScission protease was added and incubated with MBP‐AID^cry^ for 5 min at 4°C. MBP‐mCherry‐AID^cry^ and MBP‐GFP‐AID were expressed in Expi293F cells and purified with amylose resin.

Purified MBP‐fused proteins were diluted to the indicated concentrations in a buffer (20 mM HEPES pH 7.5, 150 mM NaCl, 1 mM TCEP) and phase separation of purified protein was induced by adding PEG‐8000 at a final concentration of 10%. The protein solution was loaded onto a homemade chamber with a glass slide and a coverslip attached by two parallel strips of double‐sided tape and incubated at room temperature for 30 min. Then images were taken with an LSM880 confocal microscope (Zeiss, Thornwood, NY) using 63×/1.4 oil magnification objective. The NaCl concentration in the protein solution was adjusted by 1:1 (volume ratio) mixing with buffers with different NaCl concentrations. To separate the protein in either dilute or dense phase, the solution was centrifuged at 10,000 *g* for 15 min. Proteins from the supernatant and pellet fractions were separated by SDS‐PAGE followed by Coomassie blue staining. To test the trapping of wild‐type AID, MBP‐mCherry‐AID^cry^ and MBP‐GFP‐AID were mixed at the indicated molar ratio, incubated with 10% PEG‐8000 to induce phase separation. In an alternative approach shown in Appendix Fig S9A, MBP‐mCherry‐AID^cry^ protein (5 μM) was induced to phase transition in the presence of 10% PEG‐8000, and 0.5 μM MBP‐GFP‐AID was added into the phase‐separated mixture.

ImageJ was used for quantitative measurements. A “Median filter of radius 2” and “Huang auto threshold method” were applied. Watershed analysis was performed to calculate necking shapes. The circularity range was set from 0.7 to 1.0 to exclude the dots with irregular shapes. Droplets at the edge of the image and/or fluorescence dots smaller than 0.5 μm^2^ were excluded from the analysis.

### Assessment of *AICDA* expression level with qPCR

Germinal center (GC) B cells were purified from the spleens of sheep red blood cell (SRBC)‐immunized mice (Day 9 after immunization) using magnetic‐based purification (Cato *et al*, 2011). Red blood cells were removed and the splenocytes were incubated with biotinylated antibodies (CD43, IgD, and CD11c) and then were magnetically sorted for GC B cells, followed by further purification with CD45R (B220) microbeads (130‐049‐501; Miltenyi Biotec). Splenic primary naïve B cells were cultured in a 6‐well plate at 0.5 million/mL under the stimulation of LPS and IL4 for 36 h. B‐lineage CH12F3 cells were activated with anti‐CD40, IL4, and TGF‐β for 36 h. *Aicda* ^−/−^ CH12F3 cells were transfected with 10 μg plasmids by using an NEPA21 Electroporator at 150 V for 5 ms and cultured for 36 h. U2OS cells were transfected by X‐tremeGENE™ HP DNA transfection reagent with 0.8 μg plasmids and cultured for 36 h.

Total RNA was extracted using RNAprep Pure Cell/Bacteria Kit (TIANGEN Biotech; DP430) according to the manufacturer’s protocol. One microgram of total RNA was subjected to cDNA synthesis with FastQuant RT Kit (TIANGEN Biotech; KR106). Real‐time quantitative PCR (qPCR) was carried out with a LightCycler 96 Real‐Time PCR System (Roche). Ten‐fold serial‐diluted plasmids with *AICDA* cDNA sequence were used as standard templates, and *AICDA* expression levels in different cells were calculated based on the standard curve. RT‐qPCR primer sequences are listed in Appendix Table S2.

### Single‐cell qPCR

Splenic GC B cell (B220^+^PNA^high^) was sorted into a well of 96‐well plates individually containing 2 μl 2X SuperScriptIII One‐Step RT‐PCR buffer (Invitrogen), 4 U RNase Inhibitor (Invitrogen), and 0.15% NP40 by using flow cytometry. The final volume of single‐cell resuspension was about 4 μl. A mixture of one‐step RT‐PCR primers and RT‐PCR enzymes in 1X buffer was added into the reaction to make a final 6 μl volume. One‐step RT‐PCR was performed according to the manufacturer’s protocol. The one‐step RT‐PCR products were used to quantify gene expression levels in Taqman real‐time PCR assays: *Aicda*, Mm01184115_m1, Catalog number: 4331182 (Applied Biosystems); *Gapdh*, Mm99999915_g1, Catalog number: 4331182 (Applied Biosystems).

### Statistical analysis

Statistical analyses were performed using R version 3.3.2. The number of replicates and statistical test procedures are indicated in the figure legends.

## Author contributions

**Feilong Meng:** Conceptualization; Supervision; Funding acquisition; Methodology; Writing—original draft; Project administration; Writing—review & editing. **Jiazhi Hu:** Conceptualization; Supervision; Funding acquisition; Methodology; Writing—original draft; Project administration; Writing—review & editing. **Xia Xie:** Conceptualization; Data curation; Formal analysis; Investigation; Visualization; Methodology; Writing—review & editing. **Tingting Gan:** Conceptualization; Formal analysis; Investigation; Visualization; Methodology; Writing—review & editing. **Bing Rao:** Formal analysis; Investigation; Writing—review & editing. **Weiwei Zhang:** Resources. **Rohit A Panchakshari:** Resources. **Dingpeng Yang:** Resources. **Xiong Ji:** Conceptualization. **Yu Cao:** Resources; Supervision. **Frederick W Alt:** Resources; Supervision.

In addition to the CRediT author contributions listed above, the contributions in detail are:

FLM and JH conceived of the experiments. XX, TG, FLM, and JH analyzed the data with inputs from YC, XJ, and FWA Experiments were performed by XX, TG, and BR, and WZ, RP, and DY contributed essential materials.

## Disclosure and competing interests statement

The authors declare that they have no conflict of interest.

## Supporting information



Appendix

Source Data for Appendix

Source Data for Figure 1

Source Data for Figure 2

Source Data for Figure 3

Source Data for Figure 4

Source Data for Figure 5

Source Data for Figure 6

## Data Availability

Raw sequencing data generated in this study, including HTGTS, PEM‐seq, and amplicon‐seq data, are deposited in the NCBI Sequence Read Archive (SRA accession: PRJNA668241), accessible at https://www.ncbi.nlm.nih.gov/sra/PRJNA668241.
