# Supplementary material for: C‐terminal deletion‐induced condensation sequesters AID from IgH targets in immunodeficiency
Source: EMBO J. 2022 Apr 26;41(11):e109324. doi: 10.15252/embj.2021109324 (PMC9156971; doi:10.15252/embj.2021109324)
Supplement: Supplementary file 1 — Appendix [file EMBJ-41-e109324-s001.pdf]

Appendix

C-terminal deletion-induced condensation sequesters AID from IgH targets in immunodeficiency

Xia Xie[1†], Tingting Gan[2†], Bing Rao[1†], Weiwei Zhang[2], Rohit A. Panchakshari[3], Dingpeng Yang[1], Xiong Ji[2], Yu Cao[4], Frederick W. Alt[3], Fei-Long Meng[1*] and Jiazhi Hu[2*]

Table of contents

Appendix Figure S1. Catalytic-active AID$^{\Delta C}$ mutants fail to support CSR *in vivo*.

Appendix Figure S2. AID C-terminal truncation mutants fail to initiate breaks at the *IgH* locus.

Appendix Figure S3. Two different approaches to guide AID$^{\Delta C}$ protein to *IgH* locus.

Appendix Figure S4. AID expression levels in different settings and the subcellular localization of AID variants.

Appendix Figure S5. The condensation of AID$^{\Delta C}$.

Appendix Figure S6. Trap of full-length AID in AID$^{\Delta C}$ condensates in different experimental systems.

Appendix Figure S7. AID N-terminus and assistant patch are required for AID$^{\Delta C}$ condensation.

Appendix Figure S8. Cell proliferation and CSR levels of B cells in the presence of different forms of AID.

Appendix Figure S9. AID$^{\Delta C}$ protein has the tendency of condensation *in vitro*.

Appendix Figure S10. Expression of different forms of AID protein in cells and *in vitro* reconstitution of wild-type AID trapping.

Appendix Table S1. Plasmids used in the study.

Appendix Table S2. Oligos and Reference sequence used in the study.

Appendix Table S3. Summary of AID$^{\Delta C}$ patient clinical information.

Appendix References.

Appendix Figure S1. Catalytic-active AID$^{\Delta C}$ mutants fail to support CSR *in vivo*.

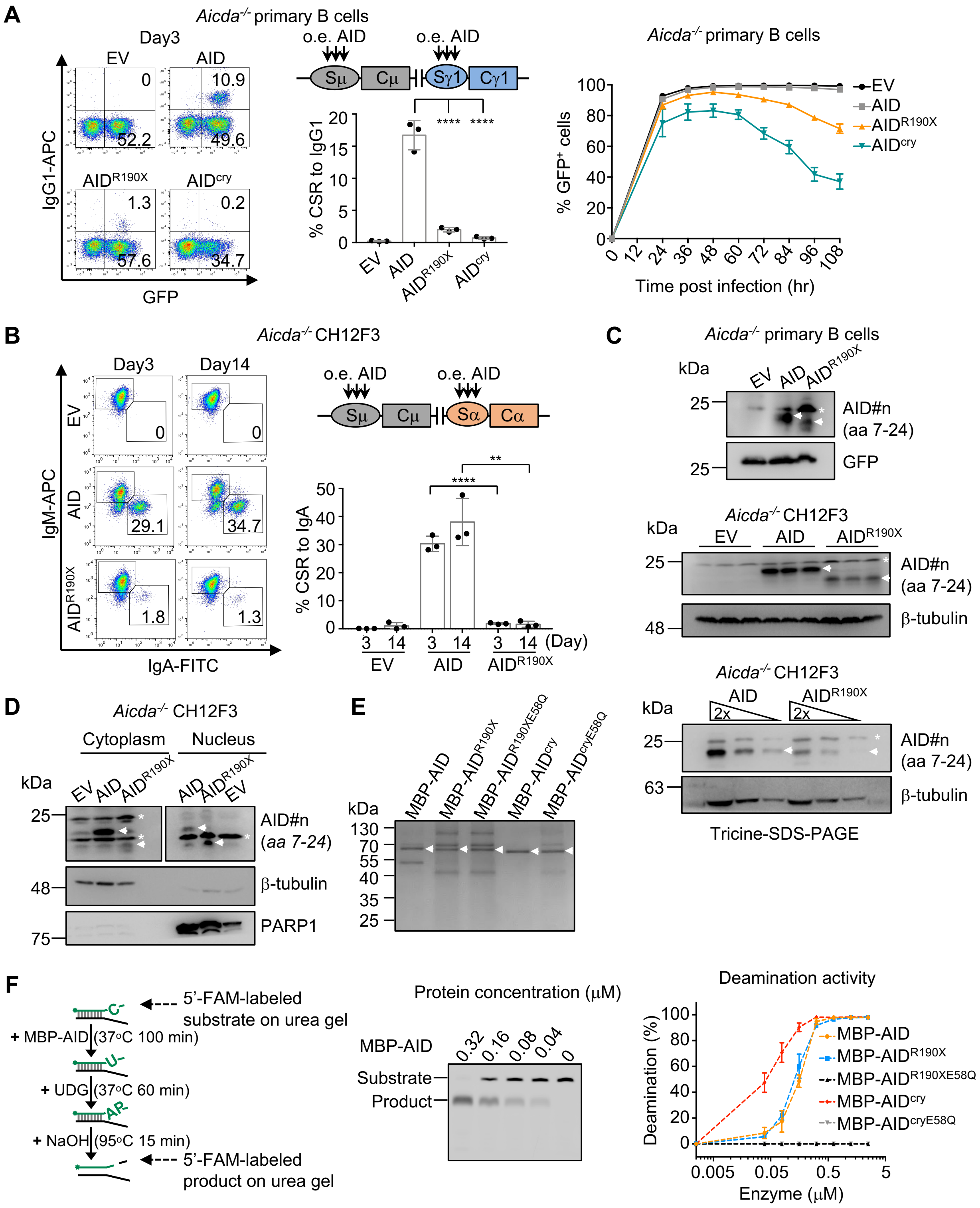

(**A**) CSR levels to IgG1 in LPS/IL4-stimulated *Aicda*$^{-/-}$ splenic primary B cells complemented with retroviral-expressed AID variants. Left, representative flow cytometry plots at Day 3 after stimulation. Co-expressed GFP indicates productively infected cells. Middle, bar plot shows the mean with SD of three biological replicates. Right, effect of AID and AID$^{\Delta C}$ proteins on the cell proliferation. GFP^{+} indicates the retroviral-infected cell population and the relative proportion of GFP^{+} cells at various time points post-infection was used to evaluate the cell viability. The mean with SD of three biological replicates is shown. One-way ANOVA followed by Dunnett's multiple comparisons test was performed. ****, p<0.0001.

(**B**) CSR levels to IgA in *Aicda*$^{-/-}$ CH12F3 cells complemented with retroviral-expressed AID mutants. Left, representative flow cytometry plots. Right, the mean with SD of three biological replicates in the bar plot. Unpaired two-tailed Student's *t*-test was performed. ****, p<0.0001. **, p<0.01.

(**C**) Representative western blots show the retroviral-expression of AID and AID^{R190X} proteins in *Aicda*$^{-/-}$ splenic primary B cells (top) and *Aicda*$^{-/-}$ CH12F3 cells (middle and bottom). Co-expressed GFP in *Aicda*$^{-/-}$ splenic B cells was used as an internal control. For *Aicda*$^{-/-}$ CH12F3 cells, representative western blots with three repeats are shown (middle). Serial diluted CH12F3 cell samples were loaded on Tricine-SDS-PAGE, and protein levels of AID and AID^{R190X} were accessed semi-quantitatively (bottom). AID proteins were detected with a custom anti-AID N terminus (7-24 aa) antibody. The arrows indicate AID and AID^{R190X} bands respectively, while the asterisk indicates an unspecific band. EV, empty vector control. Of note, AID and AID^{R190X} proteins display a bigger difference when they are separated on Tris-Glycine-SDS-PAGE, even with only a 9-aa difference, while they are not well-separated on Tricine-SDS-PAGE. Without specifically noted, Tris-Glycine-SDS-PAGE was used in this study.

(**D**) The indicated subcellular fractions of AID and AID^{R190X} from retroviral-infected *Aicda*$^{-/-}$ CH12F3 cells were analyzed by western blot. β-tubulin was used as a marker for the cytoplasmic fraction, and PARP1 was used as a nuclear marker. The arrows indicate AID and AID^{R190X} bands respectively, while the asterisks indicate unspecific bands.

(**E**) MBP-tagged AID variants purified from Expi293F cells are shown by Coomassie blue staining of SDS-PAGE. White arrows indicate the bands of MBP-fusion AID variants.

(**F**) The *in vitro* deamination activity of AID variants on branched DNA substrate. Left, a schematic illustration of *in vitro* deamination assay. Each reaction was performed with 0.5 μM DNA substrate. Middle, representative urea gel for visualization of the 5' FAM-labeled DNA substrate and product in the deamination reaction. Right, the percentage of deaminated DNA products in the indicated reaction is summarized in line plot as mean with SD of three biological replicates.

Appendix Figure S2. AID C-terminal truncation mutants fail to initiate breaks at the *IgH* locus.

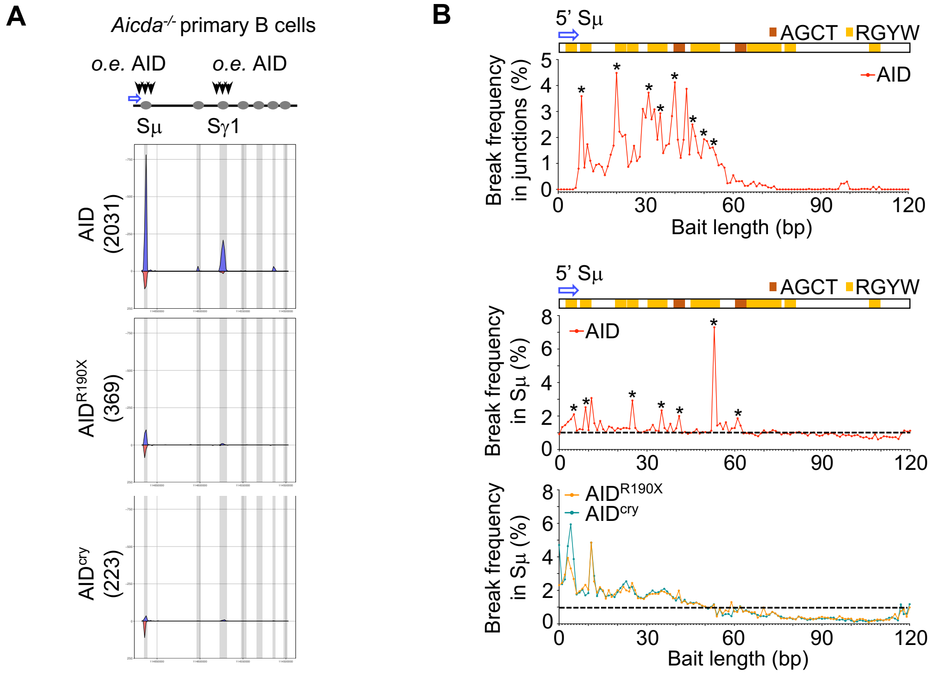

(**A**) Distribution of S junctions in the CSR-activated B cells expressing AID, AID^{R190X,} or AID^{cry}. Top, AID targeting (black arrow) in Sμ and Sγ1, and HTGTS bait primer site (blue arrow) are depicted. Bottom, linear distribution of pooled junctions in *IgH* constant gene region. Grey boxes indicate different S regions. Blue peaks at the top indicate junctions orientated from left to right, while red peaks at the bottom indicate junctions orientated from right to left. Total junction numbers in *IgH* constant gene region are labeled in parentheses.

(**B**) Top, distribution, and frequency of 5'Sμ breakpoints in junctions to downstream S regions recovered from CSR-activated B cells expressing wild-type AID. Middle and Bottom, distribution and relative frequency of 5'Sμ breakpoints in "bait-only" HTGTS reads recovered from CSR-activated B cells expressing indicated AID variants. HTGTS bait primer site (blue arrow) and positions of RGYW (R: A/G; Y: T/C; W: A/T) are depicted in the schematic diagram. Asterisks in the plots indicate the peaks coincide with the positions of RGYW motifs.

Appendix Figure S3. Two different approaches to guide AID$^{\Delta C}$ protein to *IgH* locus.

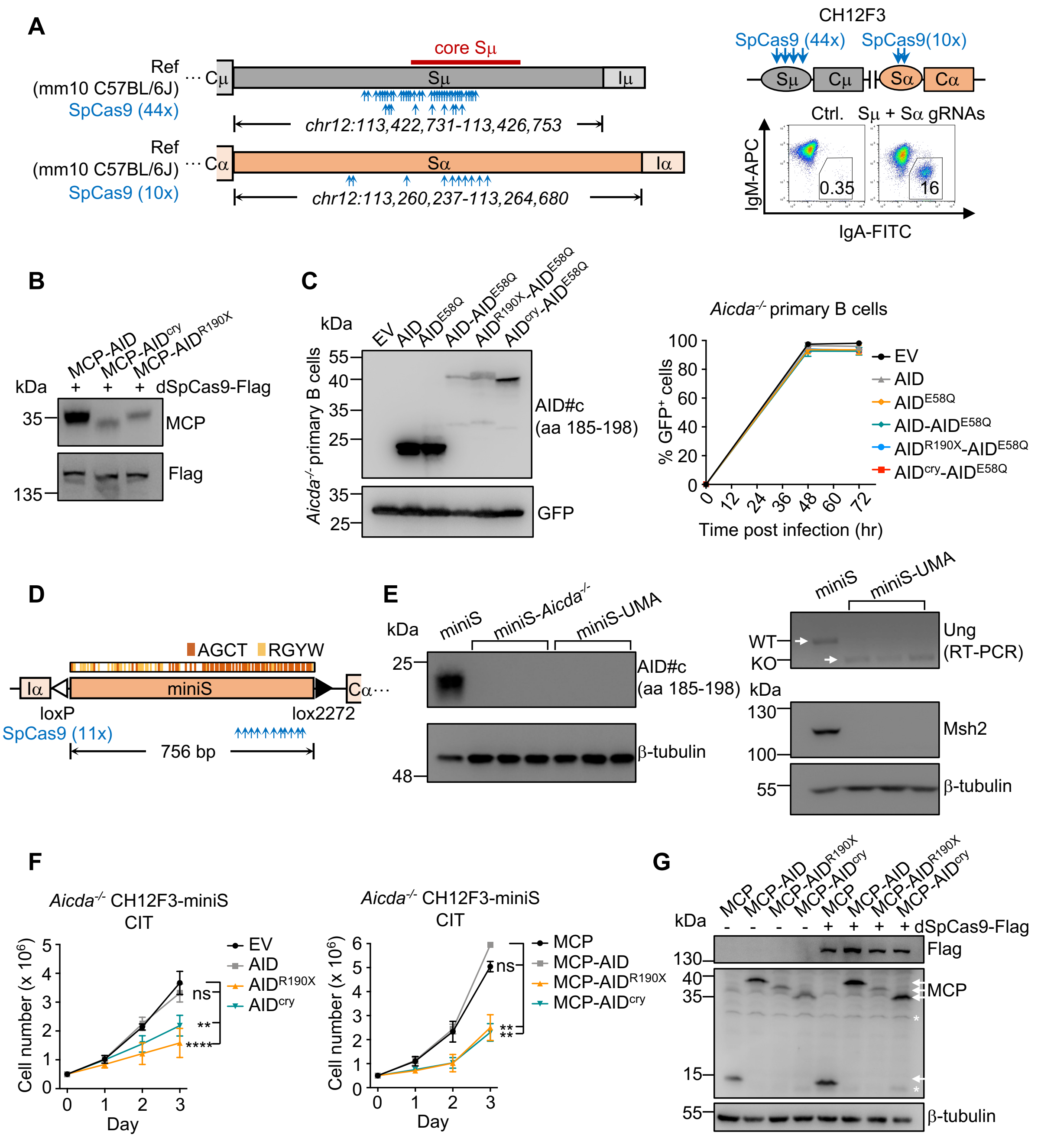

(**A**) Multiple recognition sites at Sμ and Sα regions ensured efficient SpCas9-initiated CSR to IgA in CH12F3 cells. Left, schematic illustration shows the multiple targeting sites of sgRNAs at Sμ and Sα regions. The number in the parentheses indicates sgRNA targeting times in the indicated S region. A core Sμ region is marked in red. Right, representative flow cytometry plots of Cas9-mediated CSR.

(**B**) Expression levels of MS2 coat protein (MCP)-tagged AID variants in an *Aicda*$^{-/-}$ dSpCas9^{+} CH12F3 cells. Flag-tagged dSpCas9 is used as the loading control.

(**C**) Left, expression levels of AID-AID^{E58Q} dimers in CSR-activated *Aicda*$^{-/-}$ primary B cells. Co-expressed GFP was used as an internal control. Right, the effect of AID-AID^{E58Q} dimer proteins on the cell proliferation of CSR-activated B cells is shown by the relative proportion of GFP+ cells. The mean with SD of three biological replicates is shown.

(**D**) Illustration of miniS region sequence. The positions of RGYW are depicted on top, and multiple sgRNA targeting sites in the miniS region are indicated by blue arrows.

(**E**) Western blot of AID and Msh2 proteins, and RT-qPCR of UNG in cells with the indicated genotypes. miniS-UMA: *Ung*$^{-/-}$*Msh2*$^{-/-}$*Aicda*$^{-/-}$ miniS cell line.

(**F**) Growth curves of CH12F3-miniS cells with the indicated AID expression are presented as mean with SD of three biological replicates. AID variants were retroviral-expressed with an AID-IRES-Puro cassette, and the corresponding cells were cultured in the presence of 1 μg/mL puromycin. The mean with SD of three replicates is shown. The survival data were fitted to a mixed-effects model using l-mer function from the R package 'lme4', with "Day" and "genotype" as fixed-effects parameters, and "cell number from each repeat" as the random effect parameter. The significance of the fixed effects parameters is obtained by the *t*-test. ****, $p<0.0001$; **, $p<0.01$; ns, $p>0.05$.

(**G**) Representative western blot showed expression levels of Flag-tagged dSpCas9 and MCP-tagged AID variants in the indicated *Aicda*$^{-/-}$ CH12F3-miniS cells. White arrows indicate MCP or MCP-tagged AID variant bands respectively, while asterisks indicate unspecific bands.

Appendix Figure S4. AID expression levels in different settings and the subcellular localization of AID variants.

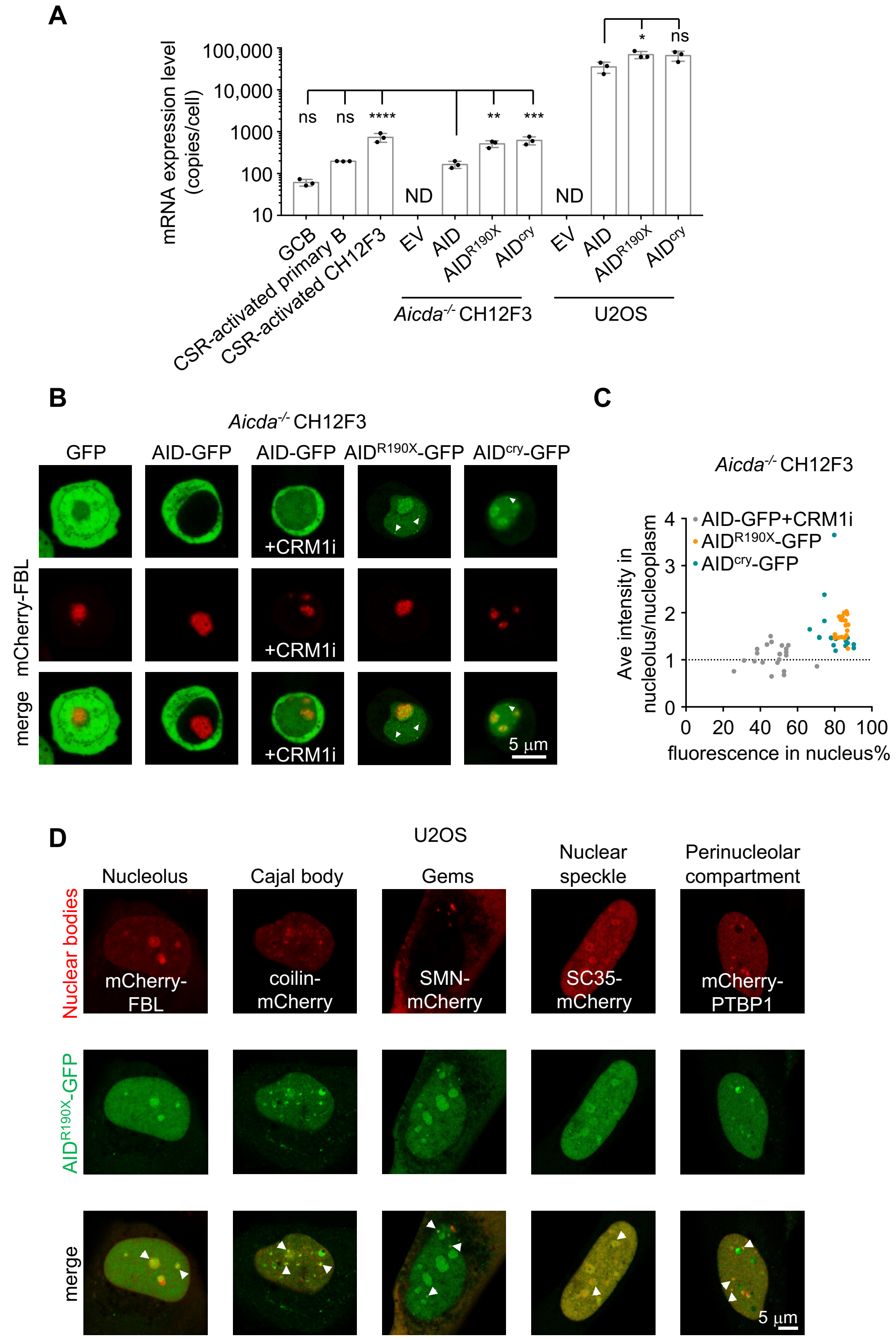

(**A**) AID expression levels under different experimental or physiological conditions were shown. Quantitative RT-PCR analysis of *Aicda* variant mRNA levels in germinal center B cells (GCB), *ex vivo* activated splenic primary B cells, cytokine-stimulated B-lineage CH12F3 cells, nucleofected *Aicda*$^{-/-}$ CH12F3 cells, and transfected U2OS cells as indicated. EV, empty vector control. mRNA levels of *Aicda* variants per cell are plotted as mean with SD of three independent replicates. ND, not detected. One-way ANOVA followed by Dunnett's multiple comparisons test was used for significance assessment. ****, $p<0.0001$; ***, $p<0.001$; **, $p<0.01$; *, $p<0.05$; ns, $p>0.05$.

(**B**) Representative images showing the subcellular localization of AID variants in nucleofected *Aicda*$^{-/-}$ CH12F3 cells. The nucleolus is indicated by co-nucleofected mCherry-fibrillarin (FBL). Scale bar, 5 μm.

(**C**) The correlation between puncta formation of AID variants in the nucleolus and the relative nuclear fluorescence intensity of AID variants in each cell (n=22 for AID-GFP+CRM1i, n=18 for AID^{R190X}-GFP and AID^{cry}-GFP, representing three biological replicates).

(**D**) AID^{R190X}-GFP (green) and mCherry-tagged markers (red) were co-transfected in U2OS cells. Membraneless organelles are indicated on top, and the used surrogate markers fused with mCherry are indicated in the first line of images. Representative fluorescence images are shown. Scale bar, 5 μm.

Appendix Figure S5. The condensation of AID$^{\Delta C}$.

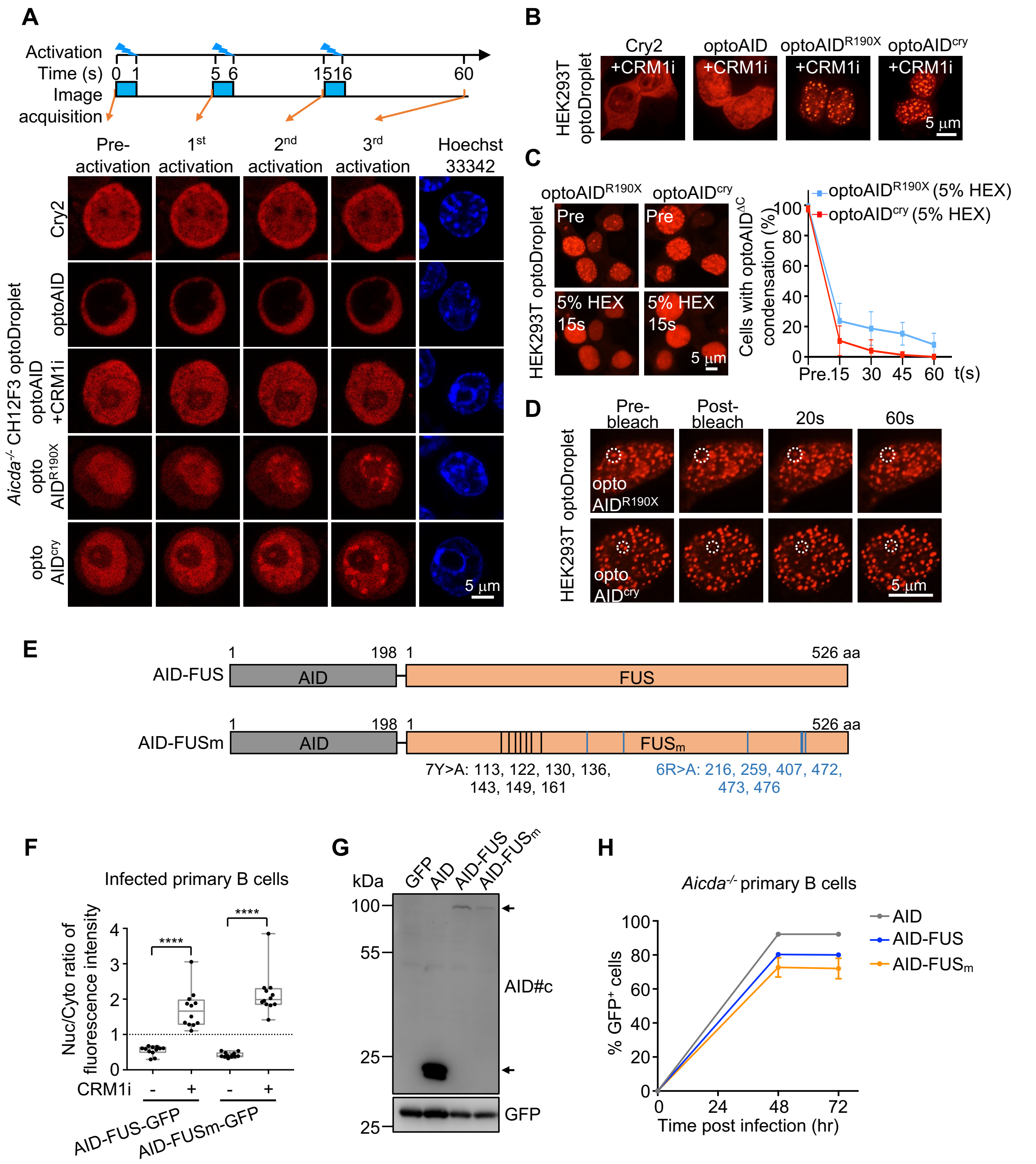

(**A**) Representative optoDroplet images for indicated proteins with increased activation. The procedure of optoDroplet assay with the blue light activation and image acquisition time points is depicted on top. Scale bar, 5 µm.

(**B**) Representative optoDroplet images for indicated AID variants with CRM1i treatment after the third blue light activation. Cells were treated 6 hours after transfection at a final

concentration of 70 nM and images were taken 18 hours after treatment. AID can be selectively trapped in nuclei by CRM1i treatment in this assay. Scale bar, 5 µm.

(**C**) 5% HEX abolishes optoAID$^{\Delta C}$ condensates. Pre-assembled optoAID$^{\Delta C}$ condensates were treated with 5% HEX for 15 s. Left, representative images for indicated optoAID$^{\Delta C}$ before (top) and after (bottom) HEX treatment. Scale bar, 5 µm. Right, the dissolving speed of pre-assembled optoAID$^{\Delta C}$ droplets in 5% HEX treatment is shown by line plots. Mean with SD of three independent biological replicates at indicated time points.

(**D**) Representative FRAP images of pre-assembled optoAID$^{\Delta C}$ droplets in HEK293T cells. Dashed line circles indicate the photobleaching sites. Scale bar, 5 µm.

(**E**) A schematic illustration of AID-FUS fusion protein constructs with mutations labeled.

(**F**) Cellular localization of AID-FUS-GFP and AID-FUS$_m$-GFP with and without the treatment of CRM1 inhibitor. The background-subtracted average fluorescence intensity in the nucleus and the cytoplasm was measured to determine the Nuc/Cyto ratio in each cell. Data are shown as box plots, values between lower quartile and upper quartile are represented by box ranges, a horizontal line within the box represents the median, and whisker extends from the minimum value to the maximum value. Each dot indicates one cell (n = 12). Unpaired two-tailed Student's *t*-test was performed. ****, p<0.0001.

(**G**) The expression level of AID-FUS fusion proteins in CSR-activated B cells was revealed by western blot. The arrows indicate AID and AID-FUS bands, respectively.

(**H**) Effect of AID-FUS synthetic proteins on the cell proliferation of CSR-activated B cells. GFP$^+$ indicates the retroviral-infected cell population, and the relative proportion of GFP$^+$ cells at various time points post-infection was used to evaluate the cell viability. The mean with SD of three biological replicates is shown.

Appendix Figure S6. Trap of full-length AID in AID$^{\Delta C}$ condensates in different experimental systems.

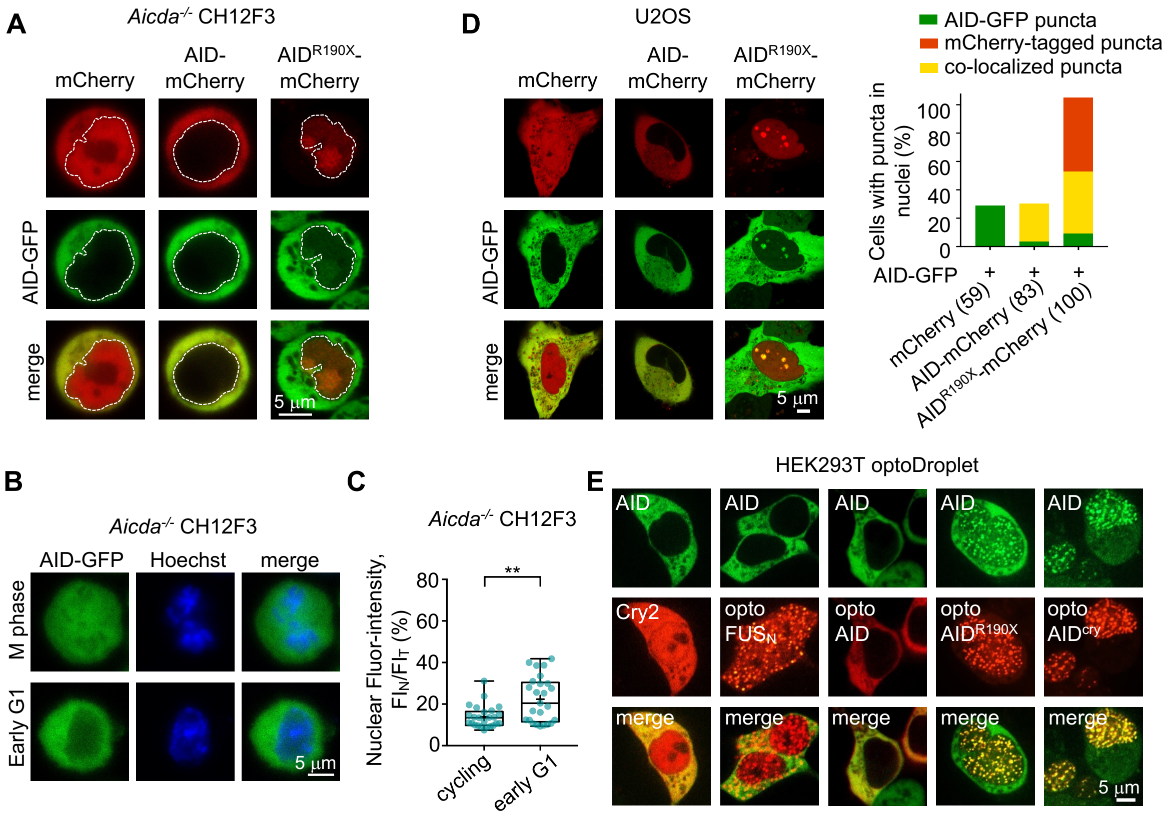

(**A**) Representative images showing the subcellular location of indicated AID variants in nucleofected *Aicda*$^{-/-}$ CH12F3 cells. White dashed line depicts the shape of the nucleus based on Hoechst staining. Scale bar, 5 µm.

(**B**) Representative images showing the subcellular localization of AID-GFP in the M phase and early G1 phase upon nocodazole release. The cell samples with nocodazole treatment for 6 h were released for 1.5 h. Scale bar, 5 µm.

(**C**) The fluorescence intensity of ectopically expressed AID-GFP in each *Aicda*$^{-/-}$CH12F3 cell nucleus was measured and normalized to the fluorescence intensity of the whole cell. The normalized fluorescence intensity in cycling cells and early G1 cells upon nocodazole release is shown by box plots, respectively. Values between lower quartile and upper quartile are represented by box ranges, a horizontal line within the box represents the median, and whisker extends from the minimum value to the maximum value. "+" indicates the mean level. Each dot indicates one cell (n = 25). Unpaired two-tailed Student's *t*-test was performed. **, $p<0.01$.

(**D**) Co-condensation of wild-type AID and AID^{R190X} in the nucleus of U2OS cells. Wild-type AID-GFP (green) and mCherry-tagged AID variants (red) were co-transfected in U2OS cells. Left, representative fluorescence images are shown. Scale bar, 5 μm. Right, quantification of co-transfected U2OS cells with puncta in nuclei is shown by bar plots and the total observed cell number of each genotype is indicated in parentheses.

(**E**) Wild-type AID protein can be selectively trapped into AID$^{\Delta C}$ condensates. AID-GFP fusion protein (green) was co-expressed with the optoDroplet system (red), and representative images after triple blue light activation are shown. Scale bar, 5 μm.

Appendix Figure S7. AID N-terminus and assistant patch are required for AID$^{\Delta C}$ condensation.

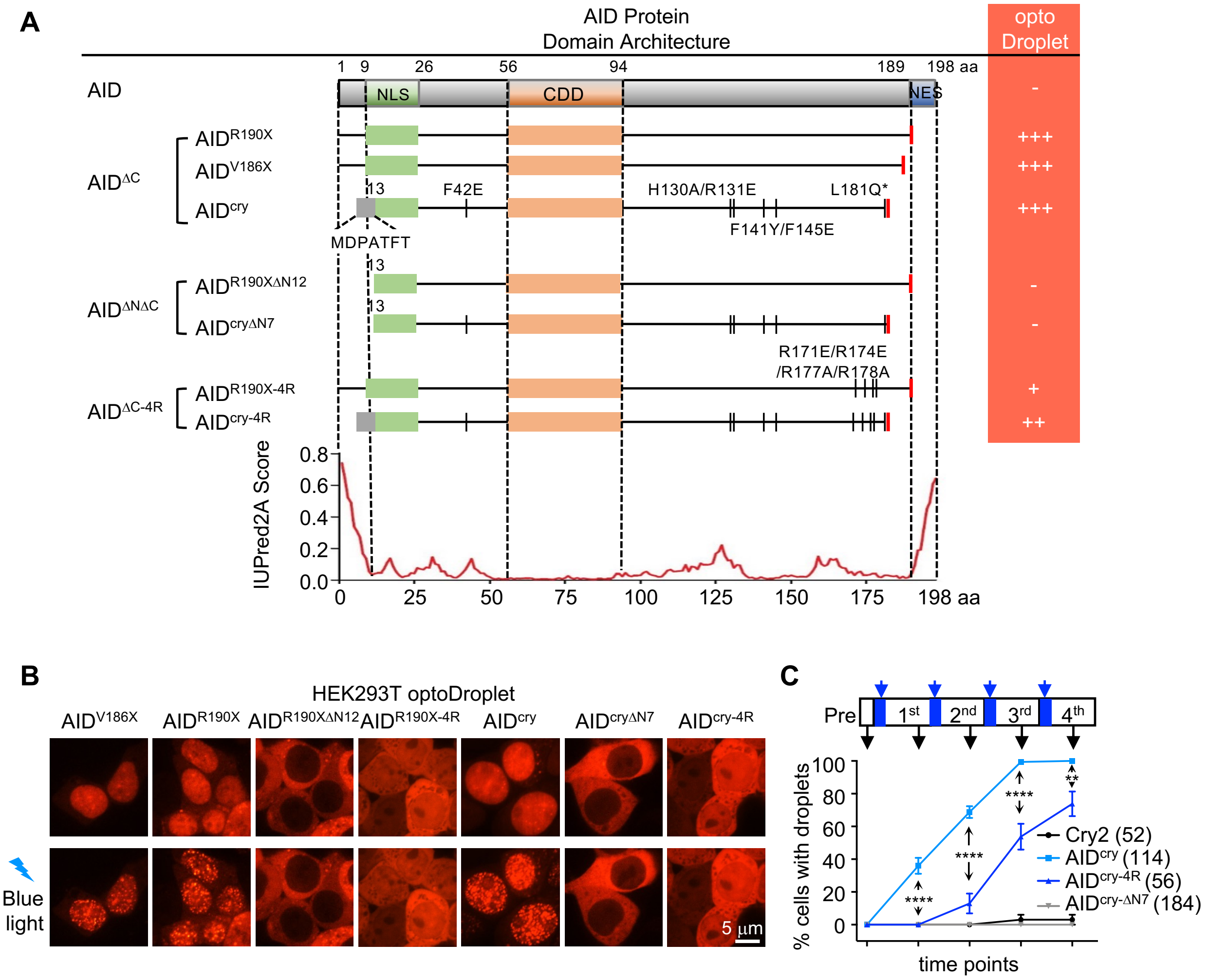

(**A**) Schematic illustration of AID mutant protein domain architectures and their abilities to form optoDroplet upon light-activation are summarized on the right panel. An IDR prediction by using IUPred2A is listed in the lower panel. IUPred2A score is shown on the *y*-axis, and amino acid position is shown on the *x*-axis. "+++": strong capacity with a cutoff of 90% cells showing optoDroplet puncta formation. "++": a lower capacity with 50-90% of cells showing puncta formation. "+": capacity with less than 50% of cells showing puncta formation. "-": empty vector control level.

(**B**) Representative optoDroplet images are shown for the indicated AID mutants before (top) and after (bottom) three times of blue light activation. AID$^{\Delta C}$ mutants, including AID^{V186X}, AID^{R190X},

and AID^{cry}, formed optoDroplet. While the $AID^{\Delta N\Delta C}$ variants ($AID^{R190X\Delta N12}$ and $AID^{cry\Delta N7}$) and $AID^{\Delta C\text{-}4R}$ ($AID^{R190X\text{-}4R}$ and $AID^{cry\text{-}4R}$) were unable to form optoDroplet. Scale bar, 5 μm.

(**C**) The optoDroplet capacity that was assessed based on the dynamics of optoDroplet formation is shown for the indicated AID^{cry} mutants. Top, the procedure of optoDroplet assay is illustrated with indicated light stimulation (blue arrow) and image acquisition (black arrow) time points. Bottom, the percentage of cells with optoDroplet formation at each time point is shown by line plots as mean with SEM, and the total acquired cell number of each genotype is indicated in parentheses. Significance assessment between AID^{cry} and $AID^{cry\text{-}4R}$ is shown at each time point. Unpaired two-tailed Student's *t*-test was performed. ****, $p<0.0001$; **, $p<0.01$.

Appendix Figure S8. Cell proliferation and CSR levels of B cells in the presence of different forms of AID.

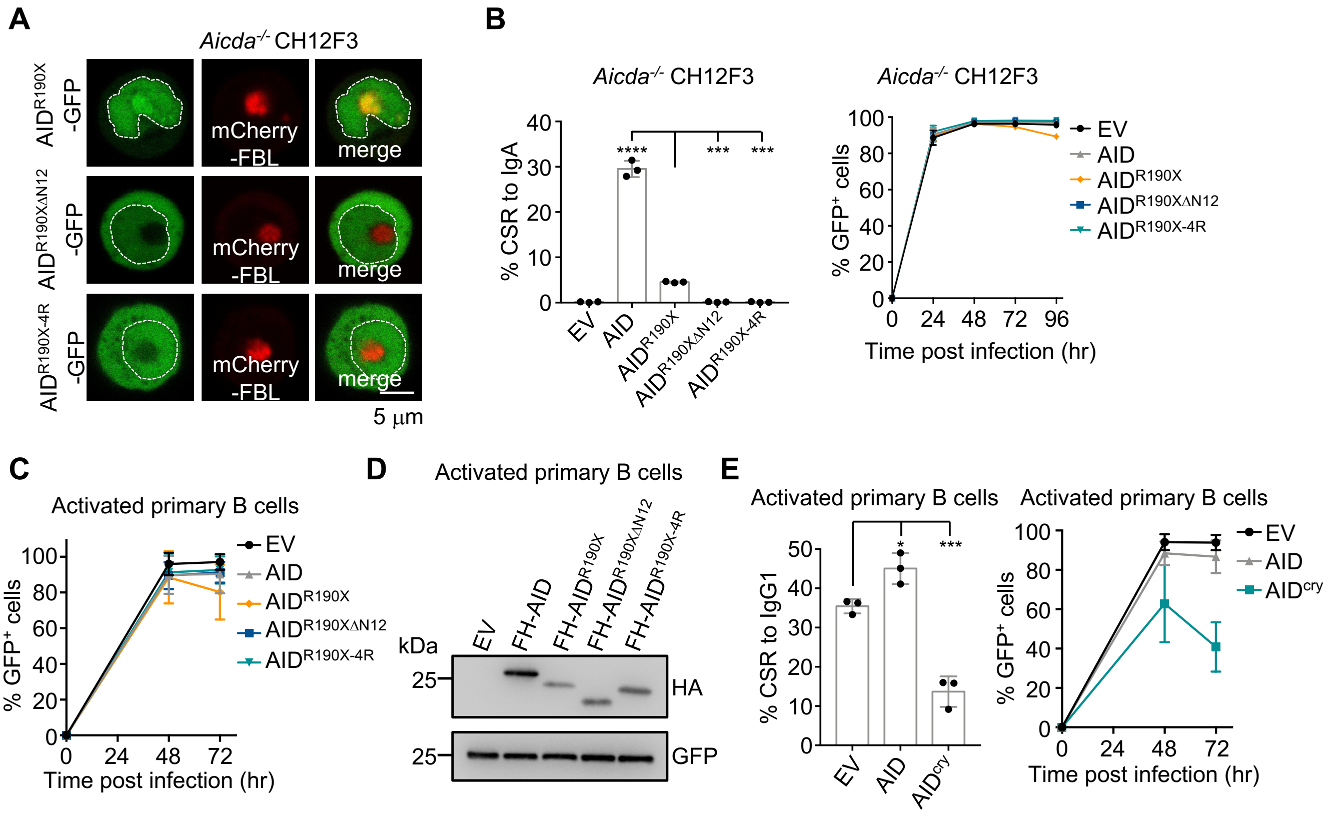

(**A**) Representative images showing the subcellular localization of indicated AID variants in nucleofected *Aicda*$^{-/-}$ CH12F3 cells. The nucleolus is indicated by mCherry-FBL. White dashed line depicts the shape of the nucleus based on Hoechst staining. Scale bar, 5 µm.

(**B**) Left, CSR levels to IgA in *Aicda*$^{-/-}$ CH12F3 cells complemented with retroviral-expressed AID mutants. The mean with SD of three biological replicates in the bar plot. Right, effect of AID mutants on the cell viability is indicated by the percentage of GFP^{+} cells. One-way ANOVA followed by Dunnett's multiple comparisons test was performed. ****, p<0.0001; ***, p<0.001.

(**C**) The effect of AID and AID mutant proteins on the cell proliferation of CSR-activated B cells is indicated by the percentage of GFP^{+} population at various time points post-infection as mean with SD (n=4 biological replicates).

(**D**) Representative western blots show the expression level of Flag-HA-tagged (FH) AID variants in CSR-activated primary B cells.

(**E**) Left, the effect of ectopically expressed AID^{cry} on the CSR levels in CSR-activated primary B cells. Right, cell viability is indicated by the percentage of GFP^{+} cells at various time points post-infection as mean with SD (n=3 biological replicates). One-way ANOVA followed by Dunnett's multiple comparisons test was performed. ***, p<0.001; *, p<0.05.

Appendix Figure S9. AID$^{\Delta C}$ protein has the tendency of condensation *in vitro*.

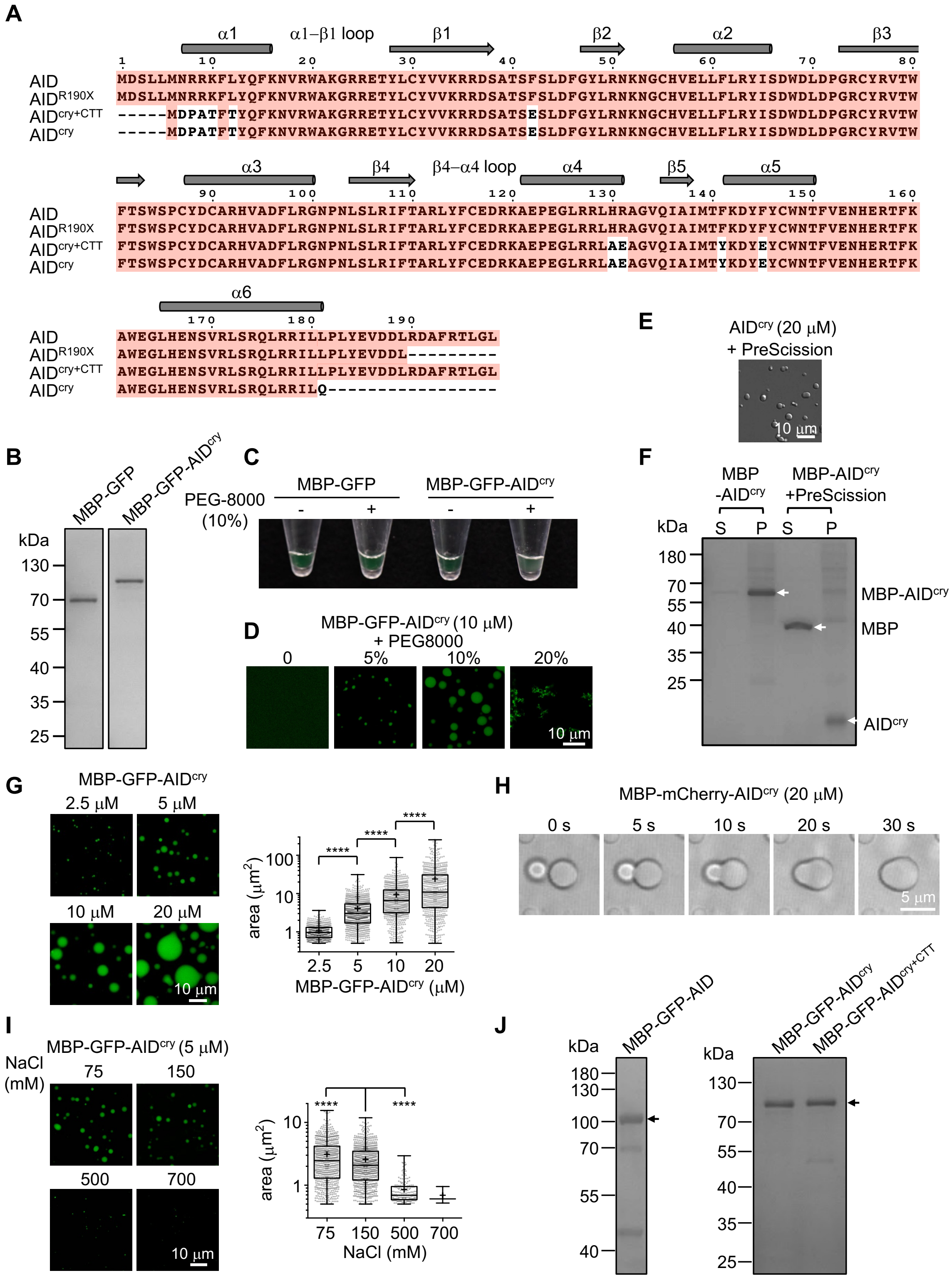

(**A**) Sequence alignment of AID, AID^{R190X}, AID^{cry}, and AID$^{cry+CTT}$. Secondary structural features are labeled.

(**B**) Purified MBP-GFP and MBP-GFP-AID^{cry} proteins were shown by Coomassie blue staining of SDS-PAGE.

(**C**) MBP-GFP-AID^{cry} protein solution turns opaque in the presence of 10% PEG-8000, while the control MBP-GFP protein stays clear under the same condition. The concentration for MBP-GFP-AID^{cry} and MBP-GFP is 10 μM.

(**D**) Representative fluorescence images showing the droplet formation of MBP-GFP-AID^{cry} in the presence of PEG8000 at the indicated concentration. The protein concentration of MBP-GFP-AID^{cry} is 10 μM. Scale bar, 10 μm.

(**E**) Representative differential interference contrast (DIC) images of AID^{cry} condensation are shown after cutting MBP tag off with PreScission enzyme. Twenty micromoles of MBP-AID^{cry} protein were used, and 10% PEG8000 was added to induce droplet formation. Scale bar, 10 μm.

(**F**) The two-phase mixtures of MBP-AID^{cry} or AID^{cry} were centrifuged to isolate the dilute phase (supernatant, S) and dense phase (pellet, P). Proteins were separated by SDS-PAGE staining with Coomassie blue. White arrows indicate the specific bands.

(**G**) *In vitro* droplet formation of MBP-GFP-AID^{cry} at different protein concentrations in the presence of 10% PEG-8000. Left, representative images of MBP-GFP-AID^{cry} condensates at the indicated protein concentrations. Scale bar, 10 μm. Right, sizes of MBP-GFP-AID^{cry} droplets at the indicated concentrations are plotted as box plots with all points listed and mean indicated by "+" (n=5 technical replicates). Values between lower quartile and upper quartile are represented by box ranges, a horizontal line within the box represents the median, and whisker extends from the minimum value to the maximum value. Unpaired two-tailed Student's *t*-test was performed. ****, $p<0.0001$.

(**H**) Time-lapse view of two fusing MBP-mCherry-AID^{cry} droplets. Differential interference contrast images are shown. Scale bar, 5 μm.

(**I**) MBP-GFP-AID^{cry} droplet formation at different concentrations of NaCl in the presence of 10% PEG-8000. The protein concentration of MBP-GFP-AID^{cry} is 5 μM. Left, representative images at the indicated salt concentrations are shown. Scale bar, 10 μm. Right, size distribution of MBP-GFP-AID^{cry} droplets at the indicated salt concentrations are shown in box plots with all points listed and mean indicated by "+" (n=5 technical replicates). One-way ANOVA followed by Dunnett's multiple comparisons was performed. ****, $p<0.0001$.

(**J**) Purified MBP-GFP-AID, MBP-GFP-AID^{cry} and MBP-GFP-AID$^{cry+CTT}$ were shown by Coomassie blue staining of SDS-PAGE. Black arrows indicate the bands of MBP-GFP-fusion AID variants.

Appendix Figure S10. Expression of different forms of AID protein in cells and *in vitro* reconstitution of wild-type AID trapping.

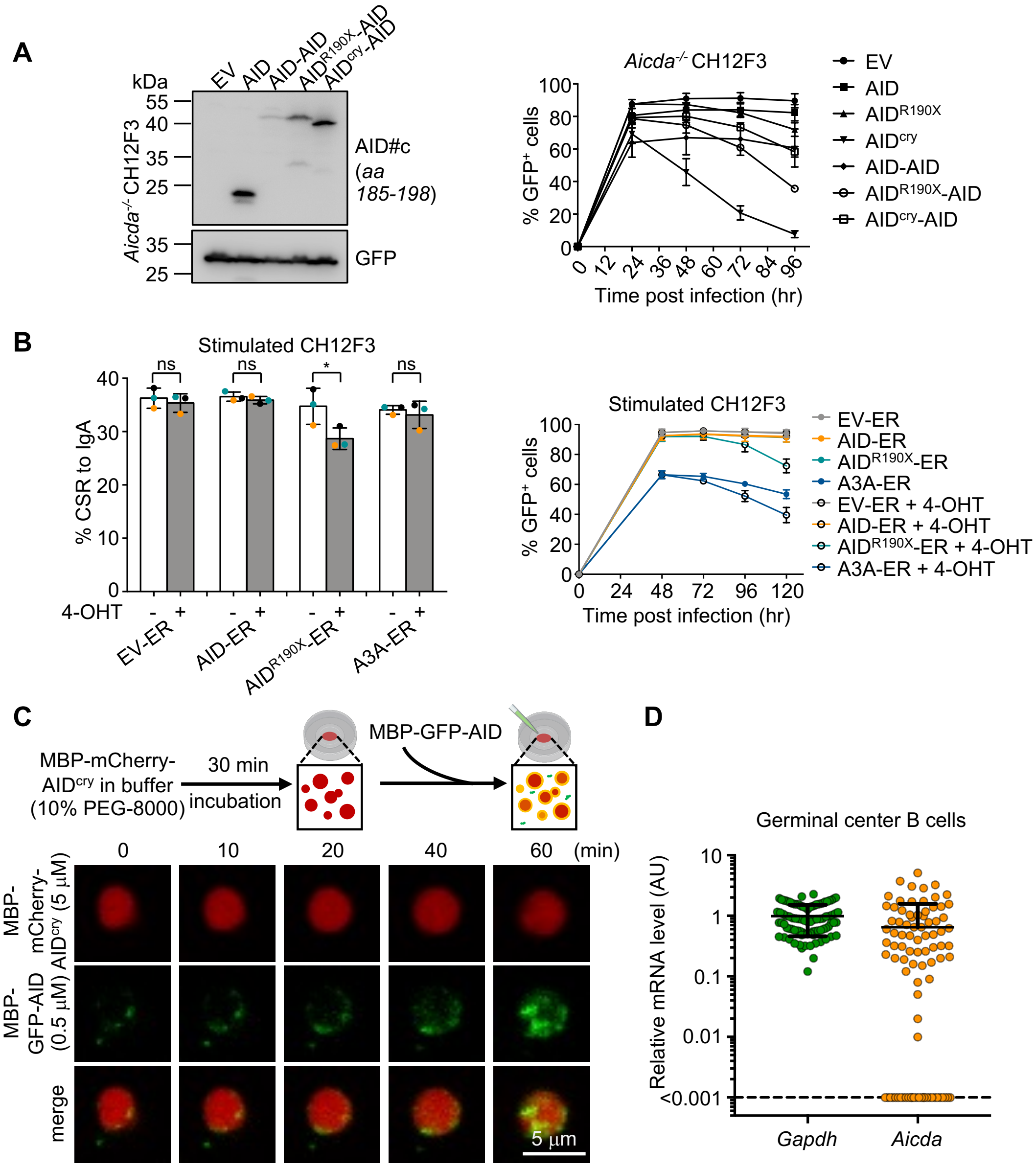

(**A**) Left, representative Western blots showing the expression level of AID and AID dimer proteins in the indicated *Aicda*$^{-/-}$CH12F3 cells. Right, the effect of AID and AID dimers on the cell viability is indicated by the percentage of the GFP^{+} population as mean with SD at various time points post-infection (n=3 biological replicates).

(**B**) Left, CSR levels in the indicated CH12F3 cells without and with the treatment of 4-hydroxy tamoxifen (4-OHT). The dominant-negative effect is accessed by comparing the CSR levels without (-) and with (+) 4-OHT induced ER fusion protein activation. Bar plot shows the mean with SD of three technical replicates. Right, cell viability is indicated by the percentage of the GFP^{+} population as mean with SD at various time points post-infection. Paired two-tailed *t*-test was performed. *, $p<0.05$; ns, $p>0.05$.

(**C**) Representative fluorescence images at the indicated time points were shown for the trapping of MBP-GFP-AID by MBP-mCherry-AID^{cry} condensates. MBP-mCherry-AID^{cry} condensation was induced by 10% PEG8000 first, and then MBP-GFP-AID was added into the two-phase mixture. Scale bar, 5 μm.

(**D**) Single-cell quantitative RT-PCR analysis of *Aicda* and *Gapdh* in splenic germinal center B cells. AU, arbitrary units. For each gene, mean Ct of four 10-cell wells (Ct^{10c}) was subjected to estimate Ct of a single cell. For individual cells, relative mRNA level was determined by 2^(Ct^{10c} + $\log_2 10$ - Ct). Each symbol represents the mRNA level in one cell (n = 90).

Appendix Table S1. Plasmids used in the study.

Name	Purpose	Reference
pMX-IRES-GFP	Retroviral infection	(Basu *et al*, 2008)
pMX-AID-IRES-GFP	Retroviral infection	(Basu *et al,* 2008)
pMX-AID^{R190X}-IRES-GFP	Retroviral infection	This study
pMX-AID^{cry}-IRES-GFP	Retroviral infection	This study
pMX-AID-AID-IRES-GFP	Retroviral infection	This study
pMX-AID^{R190X}-AID-IRES-GFP	Retroviral infection	This study
pMX-AID^{cry}-AID-IRES-GFP	Retroviral infection	This study
pMX-AID^{E58Q}-IRES-GFP	Retroviral infection	This study
pMX-AID-AID^{E58Q}-IRES-GFP	Retroviral infection	This study
pMX-AID^{R190X}-AID^{E58Q}-IRES-GFP	Retroviral infection	This study
pMX-AID^{cry}-AID^{E58Q}-IRES-GFP	Retroviral infection	This study
pMX-AID$^{R190X\Delta N12}$-IRES-GFP	Retroviral infection	This study
pMX-AID$^{R190X\text{-}4R}$-IRES-GFP	Retroviral infection	This study
pMX-AID-FUS-GFP	Retroviral infection	This study
pMX-AID-FUS$_m$-GFP	Retroviral infection	This study
pMX-AID-FUS-IRES-GFP	Retroviral infection	This study
pMX-AID-FUS$_m$-IRES-GFP	Retroviral infection	This study
pMX-FH-AID-IRES-GFP	Retroviral infection	This study
pMX-FH-AID^{R190X}-IRES-GFP	Retroviral infection	This study
pMX-FH-AID$^{R190X\Delta N12}$-IRES-GFP	Retroviral infection	This study
pMX-FH-AID^{R190X4R}-IRES-GFP	Retroviral infection	This study
pMX-IRES-puro	Retroviral infection	This study
pMX-AID-IRES-puro	Retroviral infection	This study
pMX-AID^{R190X}-IRES-puro	Retroviral infection	This study
pMX-AID^{cry}-IRES-puro	Retroviral infection	This study
pMX-MCP-IRES-puro	Retroviral infection	This study
pMX-MCP-AID-IRES-puro	Retroviral infection	This study
pMX-MCP-AID^{R190X}-IRES-puro	Retroviral infection	This study
pMX-MCP-AID^{cry}-IRES-puro	Retroviral infection	This study
pMX-MCP-mCherry-IRES-puro	Retroviral infection	This study
pLL7_lenti_dcas9_3xflag_blast	Lentiviral infection	(Liu *et al*, 2018)
pX33R-Sμ-Sirius-8xMS2	CRISPR-Sirius	(Ma *et al*, 2018)
pcDNA-MBP-AID	Protein purification	This study
pcDNA-MBP-AID^{R190X}	Protein purification	This study
pcDNA-MBP-AID^{R190XE58Q}	Protein purification	This study
pcDNA-MBP-AID^{cry}	Protein purification	This study
pcDNA-MBP-AID^{cryE58Q}	Protein purification	This study
pcDNA-MBP-GFP-AID	Protein purification	This study
pcDNA-MBP-mCherry-AID^{cry}	Protein purification	This study
pFast-MBP-AID^{cry}	Protein purification	This study
pFast-MBP-GFP	Protein purification	This study
pFast-MBP-GFP-AID^{cry}	Protein purification	This study
pFast-MBP-GFP-AID$^{cry+CTT}$	Protein purification	This study

pLL1-sgRNA(MS2)-MCP	CRISPR-guided AID targeting	(Liu *et al,* 2018)
pLL1-sgRNA(MS2)-MCP-AID	CRISPR-guided AID targeting	This study
pLL1-sgRNA(MS2)-MCP-AID^{R190X}	CRISPR-guided AID targeting	This study
pLL1-sgRNA(MS2)-MCP-AID^{cry}	CRISPR-guided AID targeting	(Liu *et al,* 2018)
pLL1-sgRNA(SpCas9)-dSpCas9	CRISPR-guided AID targeting	(Liu *et al,* 2018)
pEGFP-N1	Transfection	Clontech#6085-1
pEGFP-N1-AID	Transfection	This study
pEGFP-N1-AID^{R190X}	Transfection	This study
pEGFP-N1-AID$^{R190X-4R}$	Transfection	This study
pEGFP-N1-AID$^{R190X\Delta N12}$	Transfection	This study
pmCherry-N1	Transfection	This study
pmCherry-N1-AID	Transfection	This study
pmCherry-N1-AID^{R190X}	Transfection	This study
pmCherry-C1-FBL	Transfection	This study
pEGFP-C1-FBL	Transfection	This study
pmCherry-N1-coilin	Transfection	This study
pmCherry-N1-SMN	Transfection	This study
pmCherry-N1-SC35	Transfection	This study
pmCherry-C1-PTBP1	Transfection	This study
pHR-mCh-Cry2WT	OptoDroplet	Addgene#101221
pHR-FUS$_N$-mCh-Cry2WT	OptoDroplet	Addgene#101223
pHR-AID-mCh-Cry2WT	OptoDroplet	This study
pHR-AID^{R190X}-mCh-Cry2WT	OptoDroplet	This study
pHR-AID^{cry}-mCh-Cry2WT	OptoDroplet	This study
pHR-AID^{V186X}-mCh-Cry2WT	OptoDroplet	This study
pHR-AID$^{R190X-4R}$-mCh-Cry2WT	OptoDroplet	This study
pHR-AID^{cry-4R}-mCh-Cry2WT	OptoDroplet	This study
pHR-AID$^{R190X\Delta N12}$-mCh-Cry2WT	OptoDroplet	This study
pHR-AID$^{cry\Delta N7}$-mCh-Cry2WT	OptoDroplet	This study
pCMV-mCh-Cry2WT	OptoDroplet	This study
pCMV-AID-mCh-Cry2WT	OptoDroplet	This study
pCMV-AID^{R190X}-mCh-Cry2WT	OptoDroplet	This study
pCMV-AID^{cry}-mCh-Cry2WT	OptoDroplet	This study

Appendix Table S2. Oligos and Reference sequence used in the study.

Purpose	Name	Sequence
CRISPR-guided AID targeting	Sμ-sgRNA (SpCas9)	GGTGAGCTGGGCTGAGCTG
CRISPR-guided AID targeting	Sα-sgRNA (SpCas9)	AGCTAGGCTGGAATAGGCT
HTGTS	5'-Iμ-BIO	/5bio/CAGACCTGGGAATGTATGGT
HTGTS	5'-Iμ-RED	CACACAAAGACTCTGGACCTC
Iγ3_Bait-for PEM-seq	Iγ3-sgRNA (SaCas9)	AAATCTGCAGGACTAACAAAGGTGAGT
PEM-seq	5'-Iγ3-BIO	/5bio/AGCTGGCAGGACCAAATTCG
PEM-seq	5'-Iγ3-RED	GAGTCAGCAGAGAAGAGGTGG
PEM-seq	BA-up	/5Phor/CCACGCGTGCTCTACANNNTNNNNTNNNTGATCGGAAGAGCACACGT CTGAACTCCAGT-NH_2
PEM-seq	BA-dn	TGTAGAGCACGCGTGGNNNNNN-NH_2
PEM-seq	I7-Barcode	CAGAAGACGGCATACGAGATxxxxxxGTGACTGGAGTTCAGACGTGTGC
PEM-seq	P5-I5	AATGATACGGCGACCACCGAGATCTACACACACTCTTTCCCTACACGACGC
Ung deletion	Ung-sgRNA3; sgRNA4	GCTTGATTAGGTCCGTGATA; TTAATCCTCAGCACCCACGT
Msh2 deletion	Msh2-sgRNA1; sgRNA2	ACATCGTTGAGCGTCTGCAT; GAGTGTCTTACGAGGCGTGC
Aicda deletion	Aicda-sgRNA1;2;3;4	GTAGGTCTCATGCCGTCCCT; GCCGAAGTCCAGTGAGCAGG; GCGAGATGCATTTCGTATGT; GGATTTTGAAAGCAACCTCC
Genotyping	Ung-genoF	CTTGGTGTGTTTCCTGGTGG
Genotyping	Ung-genoR	GTTAGACGCCAAGAACAGCC
Genotyping	Ung-semiRT-F	GAGCAGGTGTTCACATGGAC
Genotyping	Ung-semiRT-R	TCACAGCTCCTTCCAGTTGA
Genotyping	Msh2-genoF	AGGGGAAAGTACGTTGTGCT
Genotyping	Msh2-genoR	AGTCTGGGCAAACTCCTCAA
Genotyping	Aicda-genoF	TGACTTTCTTCTCCAACTCA
Genotyping	Aicda-genoR	GTCGTCTCCATATGCTTTTCCATGC
MiniS-seq (1st step)	Iα-intron-F	CCATCTGGACTCCTCTGCTC
MiniS-seq (1st step)	Sα-intron-R	TTCAGGAAAAAGGACCATGC
Quantitative RT-PCR	Aicda-qRT-F	GCTACGTGGTGAAGAGGAGA
Quantitative RT-PCR	Aicda-qRT-R	CCCAGTCTGAGATGTAGCGT
Quantitative RT-PCR	GFP-qRT-F	GGCTAACCAGACAACTTCGG
Quantitative RT-PCR	GFP-qRT-R	GTTTACGTCGCCGTCCAG
Single cell RT-Q-PCR	Aicda-1st-F (1st step)	CACACAACAGCACTGAAGCA
Single cell RT-Q-PCR	Aicda-1st-R (1st step)	CCTTGCGGTCTTCACAGAA
Single cell RT-Q-PCR	Gapdh-1st-F (1st step)	CGTCCCGTAGACAAAATGGT

Single cell RT-Q-PCR	Gapdh-1st-R (1st step)	CTAAGCAGTTGGTGGTGCAG
MiniS Sequence (*underlined*: sequences present in the parental RMCE cell line(Han *et al*, 2011); *highlighted*: loxP sites used in RMCE; *red*: PCR primer sites; *bolded lower case*: core Sμ sequence):	*CTATGCATCAAGCTTGGTACCGAGCTCGGATCCACTAGTAACGGCCGCCAGTGTGCTGGAATTCAGGccatctggactcctctgctcAT*CGCGATATCGTCGACTACGGGGATAACTTCGTATAATGTATGCTATACGAAGTTATGGGGCCGCTCTAGCCTCGAGTACGTAAGATCTGTCGACGATATCGGATCCATCGCG**agcttgctgagcaaaattaagggaacaaggttgagagccctagtaagcgaggctctaaaaagcatggctgagctgagatgggtgggcttctctgagcgcttctaaaatgcgctaaactgaggtgattactctgaggtaagcaaagctgggcttgagccaaaatgaagtagactgtaatgaactggaatgagctgggccgctaagctaaactaggctggcttaaccgagatgagccaaactggaatgaacttcattaatctaggttgaatagagctaaactctactgcctacactggactgttctgagctgagatgagctggggtgagctcagctatgctacgctgtgttggggtgagctgatctgaaatgagctactctggagtagctgagatggggtgagatggggtgagctgagctgggctgagctggactgagctgagctagggtgagctgagctgggtgagctgagctaagctggggtgagctgagctgagcttgactgagctagggtgagctggactgagctggggtgagctgagctgagctggggtaagctgggatgagctggggtgagctgagctgagctggagtgagctgagctgggctgagctggggtgagctgggctgggctgagctggggtgagctgggctgagctggggtgagctgagctggggtgagctgagctgagctggggtgagctgagctgagctggggtgagctgagctggggtgagctgagctgagctgggctgagc**TGACGCGATATAACTTCGTATAGGAGACTTTATACGAAGTTATCTCGATGAAGGGGTTCTCTAGATCGAGAATTGT*AAAGACTACCTGCAGGTCATGTTCAAAGTCTATACAGCCAGAACTGTTGGTCAGCTCCGACTGCAGGTACACGATGCAGCAGCTGTGTGATACTGGGCTAGGTTCTCCTGTATAAAGAAGAGAAAGgcatggtccttttcctgaaCCTG*

Appendix Table S3. Summary of AID$^{\Delta C}$ patient clinical information.

Patient	Diagnosis Age (yr)	AID	Ig levels* IgM (g/L)	IgG (g/L)	IgA (g/L)	IgE (IU/mL)	SHM (*in vivo*)	Recurrent infection	Reference
JP41	*NA*	R190X/+	1.6	1.6	0.29	*NA*	*NA*	*NA*	(Ta *et al*, 2003)
JP42†	*NA*	R190X/+	2.9	2.8	2.2	*NA*	*NA*	*NA*	(Ta *et al,* 2003)
JP43	*NA*	R190X/+	11.3	0.28	<0.05	*NA*	*NA*	*NA*	(Ta *et al,* 2003)
1-I-2	58	R190X/+	4.05	1.28	0.1	<5	normal	+	(Imai *et al*, 2005)
1-II-2	36	R190X/+	2.85	3.46	0.51	<5	normal	-	(Imai *et al,* 2005)
1-II-3	34	R190X/+	3.02	6.33	0.39	<5	normal	-	(Imai *et al,* 2005)
1-III-1	8	R190X/+	1.22	7.58	0.45	<5	normal	-	(Imai *et al,* 2005)
2-I-1(JP42)†	41	R190X/+	2.93	2.82	2.2	<5	normal	+	(Imai *et al,* 2005)
2-II-2	13	R190X/+	2.34	0.21	<0.05	<5	decreased	+	(Imai *et al,* 2005)
3-II-1	18	R190X/+	2.34	0.38	<0.05	<5	normal	+	(Imai *et al,* 2005)
4-I-1	*NA*	R190X/+	2.03	1.3	<0.05	*NA*	*NA*	*NA*	(Durandy *et al*, 2007; Zahn *et al*, 2014)
5-I-1	*NA*	V186X/+	2.02	5.41	1.19	*NA*	*NA*	*NA*	(Durandy *et al,* 2007; Zahn *et al,* 2014)
5-II-1	*NA*	V186X/+	0.84	*NA*	<0.05	*NA*	decreased	*NA*	(Durandy *et al,* 2007; Zahn *et al,* 2014)
5-II-2	*NA*	V186X/+	1.32	4.71	<0.05	*NA*	*NA*	*NA*	(Durandy *et al,* 2007; Zahn *et al,* 2014)
AD-AID#12	71	R190X/+	2.03	1.3	<0.07	*NA*	decreased	+	(Cantaert *et al*, 2016)
AD-AID#13	13	R190X/+	1.56	<1.58	<0.29	*NA*	decreased	-	(Cantaert *et al*, 2016)
AD-AID#14	*NA*	R190X/+	*NA*	*NA*	*NA*	*NA*	decreased	-	(Cantaert *et al*, 2016)
AD-AID#18	*NA*	V186X/+	*NA*	*NA*	*NA*	*NA*	decreased	*NA*	(Cantaert *et al*, 2016)
AD-AID#28	26	R190X/+	10.3	0.83	<0.05	*NA*	decreased	+	(Cantaert *et al.*, 2016; Kasahara *et al*, 2003)

*: Data retrieved from the references.
NA: Data not available.
†: Same patient.

Appendix References

Basu U, Wang Y, Alt FW (2008) Evolution of phosphorylation-dependent regulation of activation-induced cytidine deaminase. *Mol Cell* 32: 285-291

Cantaert T, Schickel JN, Bannock JM, Ng YS, Massad C, Delmotte FR, Yamakawa N, Glauzy S, Chamberlain N, Kinnunen T *et al* (2016) Decreased somatic hypermutation induces an impaired peripheral B cell tolerance checkpoint. *J Clin Invest* 126: 4289-4302

Durandy A, Taubenheim N, Peron S, Fischer A (2007) Pathophysiology of B-cell intrinsic immunoglobulin class switch recombination deficiencies. *Adv Immunol* 94: 275-306

Han L, Masani S, Yu K (2011) Overlapping activation-induced cytidine deaminase hotspot motifs in Ig class-switch recombination. *Proc Natl Acad Sci U S A* 108: 11584-11589

Imai K, Zhu Y, Revy P, Morio T, Mizutani S, Fischer A, Nonoyama S, Durandy A (2005) Analysis of class switch recombination and somatic hypermutation in patients affected with autosomal dominant hyper-IgM syndrome type 2. *Clin Immunol* 115: 277-285

Kasahara Y, Kaneko H, Fukao T, Terada T, Asano T, Kasahara K, Kondo N (2003) Hyper-IgM syndrome with putative dominant negative mutation in activation-induced cytidine deaminase. *J Allergy Clin Immunol* 112: 755-760

Liu LD, Huang M, Dai P, Liu T, Fan S, Cheng X, Zhao Y, Yeap LS, Meng FL (2018) Intrinsic Nucleotide Preference of Diversifying Base Editors Guides Antibody Ex Vivo Affinity Maturation. *Cell Rep* 25: 884-892 e883

Ma H, Tu LC, Naseri A, Chung YC, Grunwald D, Zhang S, Pederson T (2018) CRISPR-Sirius: RNA scaffolds for signal amplification in genome imaging. *Nat Methods* 15: 928-931

Ta VT, Nagaoka H, Catalan N, Durandy A, Fischer A, Imai K, Nonoyama S, Tashiro J, Ikegawa M, Ito S *et al* (2003) AID mutant analyses indicate requirement for class-switch-specific cofactors. *Nat Immunol* 4: 843-848

Zahn A, Eranki AK, Patenaude AM, Methot SP, Fifield H, Cortizas EM, Foster P, Imai K, Durandy A, Larijani M *et al* (2014) Activation induced deaminase C-terminal domain links DNA breaks to end protection and repair during class switch recombination. *Proc Natl Acad Sci U S A* 111: E988-997
