# Supplementary figures and images for: C‐terminal deletion‐induced condensation sequesters AID from IgH targets in immunodeficiency
Source: EMBO J. 2022 Apr 26;41(11):e109324. doi: 10.15252/embj.2021109324 (PMC9156971; doi:10.15252/embj.2021109324)

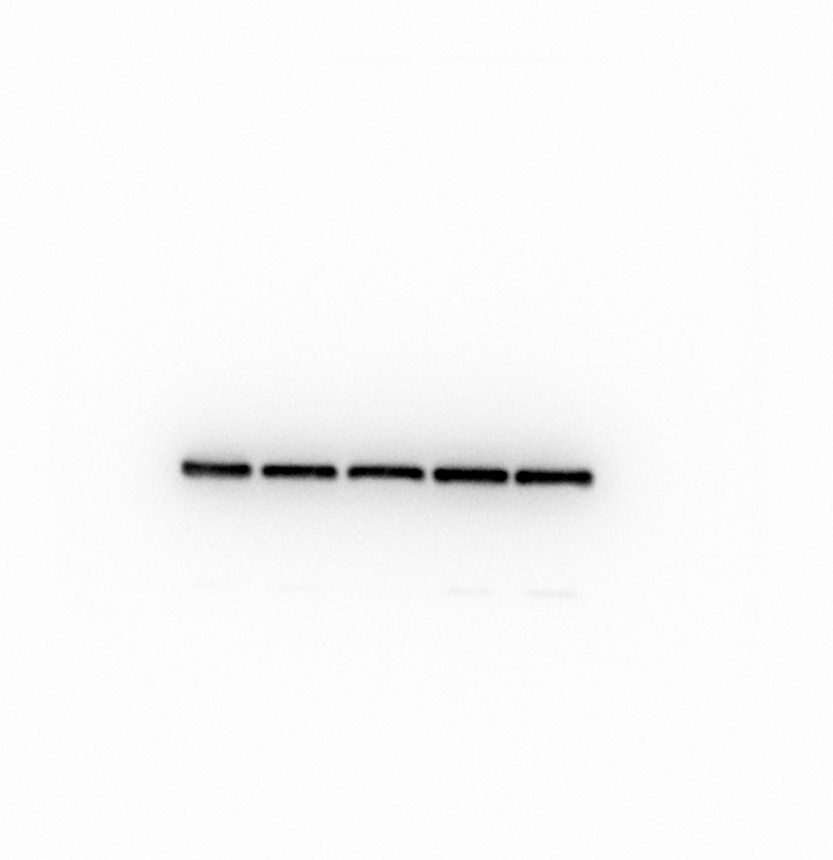

Supplement: Supplementary file 2 — Source Data for Appendix [file EMBJ-41-e109324-s008.zip › EMBOJ-2021-109324_Source_Data_Appendix/Appendix FigS8/FigS8D/FigS8D_GFP_WB.tif]

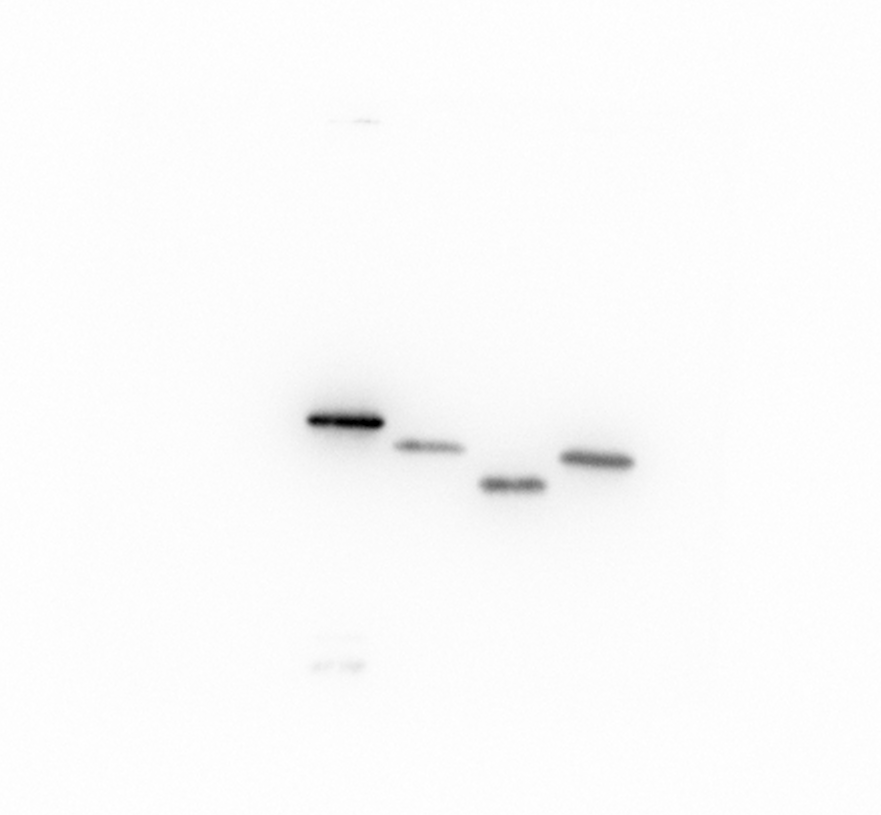

Supplement: Supplementary file 2 — Source Data for Appendix [file EMBJ-41-e109324-s008.zip › EMBOJ-2021-109324_Source_Data_Appendix/Appendix FigS8/FigS8D/FigS8D_HA_WB.tif]

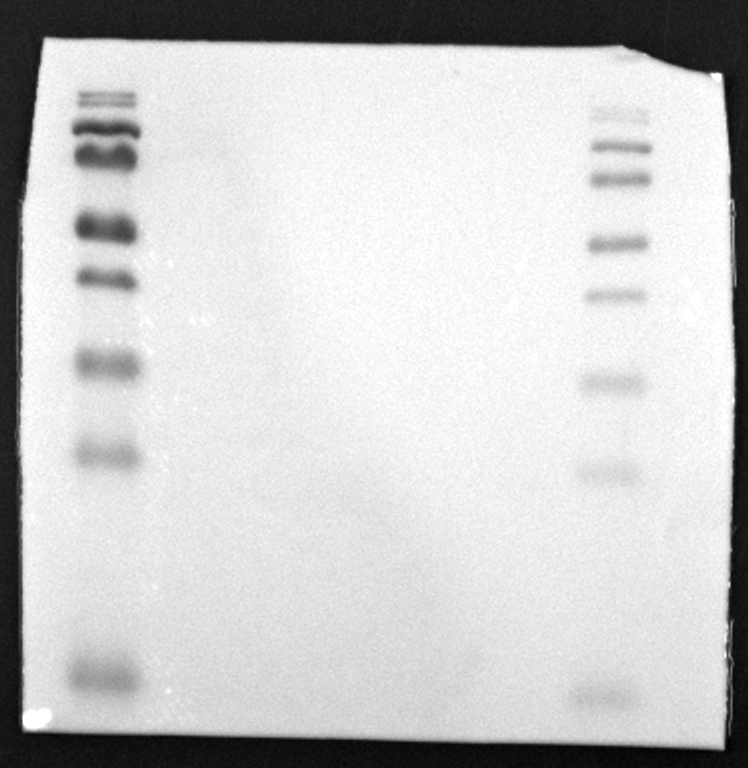

Supplement: Supplementary file 2 — Source Data for Appendix [file EMBJ-41-e109324-s008.zip › EMBOJ-2021-109324_Source_Data_Appendix/Appendix FigS8/FigS8D/FigS8D_GFP_marker.tif]

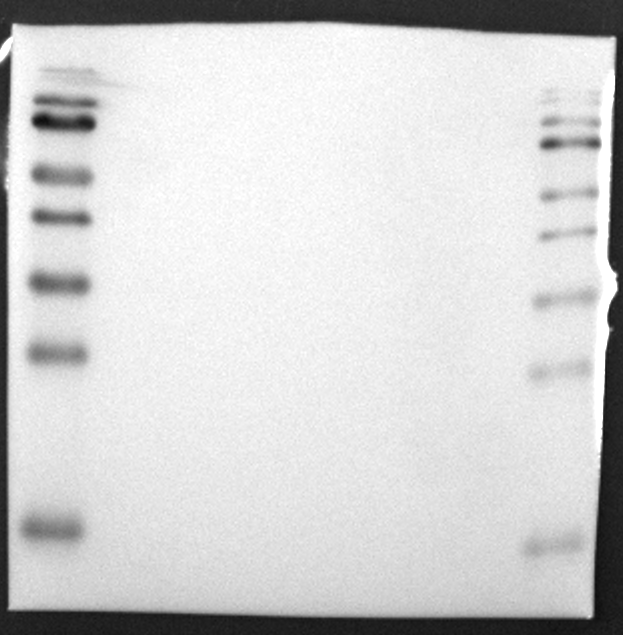

Supplement: Supplementary file 2 — Source Data for Appendix [file EMBJ-41-e109324-s008.zip › EMBOJ-2021-109324_Source_Data_Appendix/Appendix FigS8/FigS8D/FigS8D_HA_marker.tif]

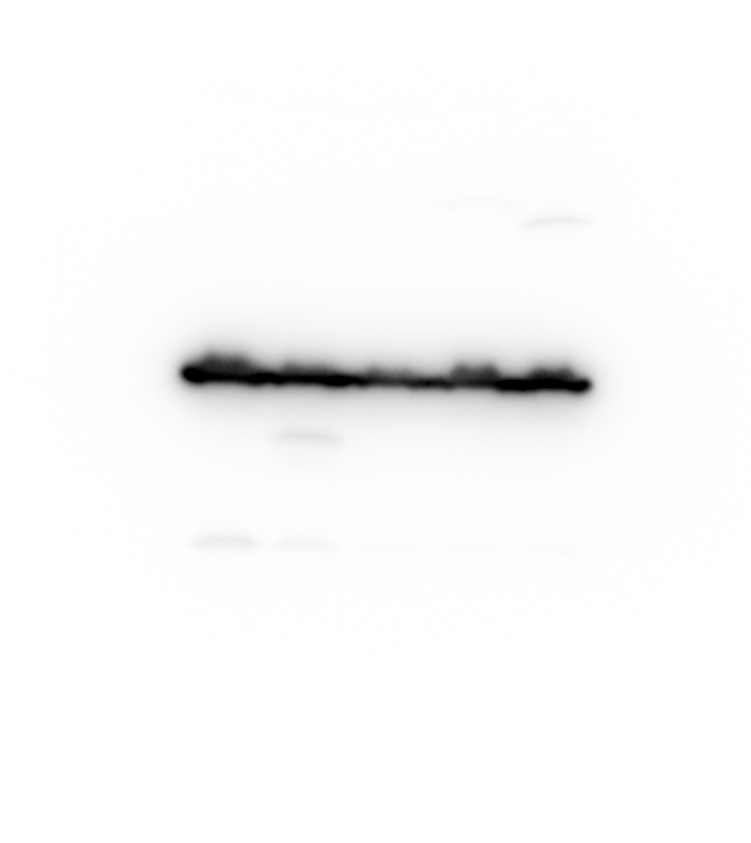

Supplement: Supplementary file 2 — Source Data for Appendix [file EMBJ-41-e109324-s008.zip › EMBOJ-2021-109324_Source_Data_Appendix/Appendix FigS10/FigS10A/FigS10A_GFP_WB.tif]

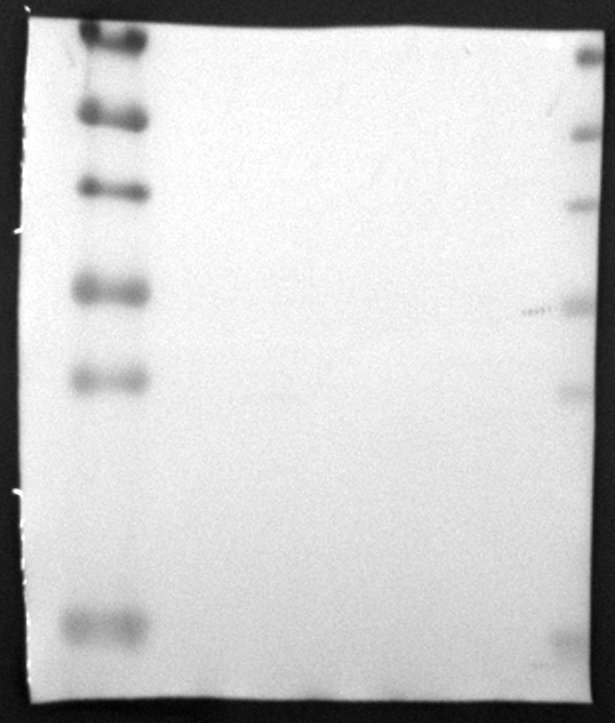

Supplement: Supplementary file 2 — Source Data for Appendix [file EMBJ-41-e109324-s008.zip › EMBOJ-2021-109324_Source_Data_Appendix/Appendix FigS10/FigS10A/FigS10A_GFP_marker.tif]

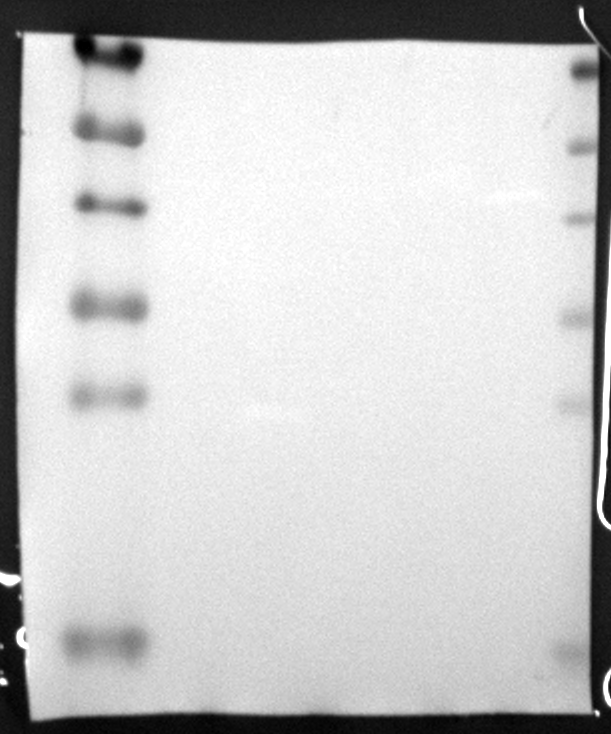

Supplement: Supplementary file 2 — Source Data for Appendix [file EMBJ-41-e109324-s008.zip › EMBOJ-2021-109324_Source_Data_Appendix/Appendix FigS10/FigS10A/FigS10A_AID_marker.tif]

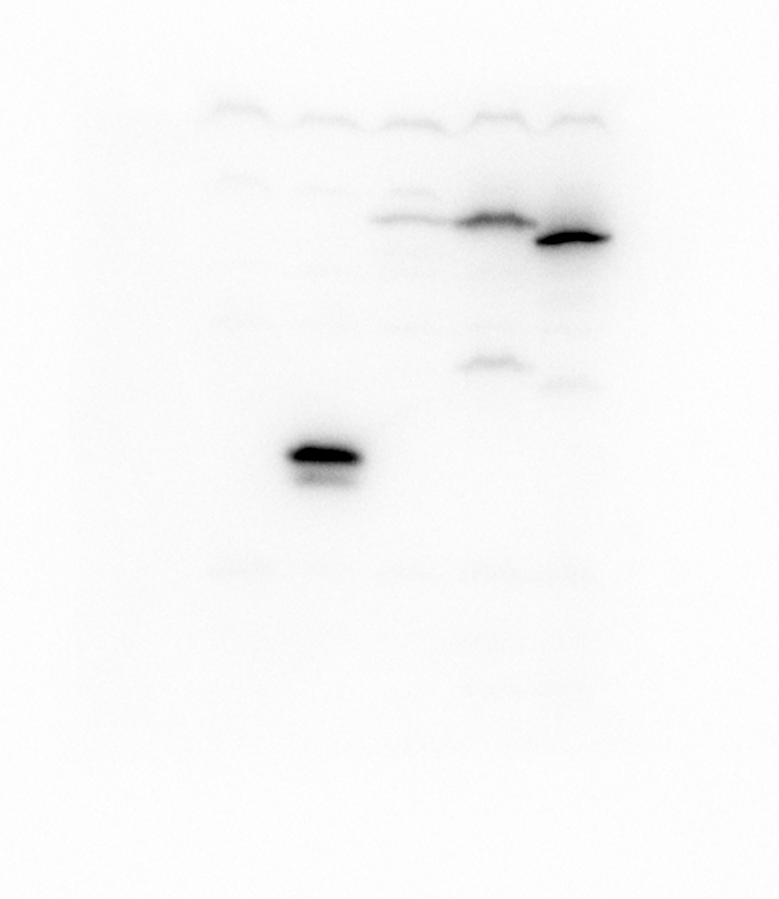

Supplement: Supplementary file 2 — Source Data for Appendix [file EMBJ-41-e109324-s008.zip › EMBOJ-2021-109324_Source_Data_Appendix/Appendix FigS10/FigS10A/FigS10A_AID_WB.tif]

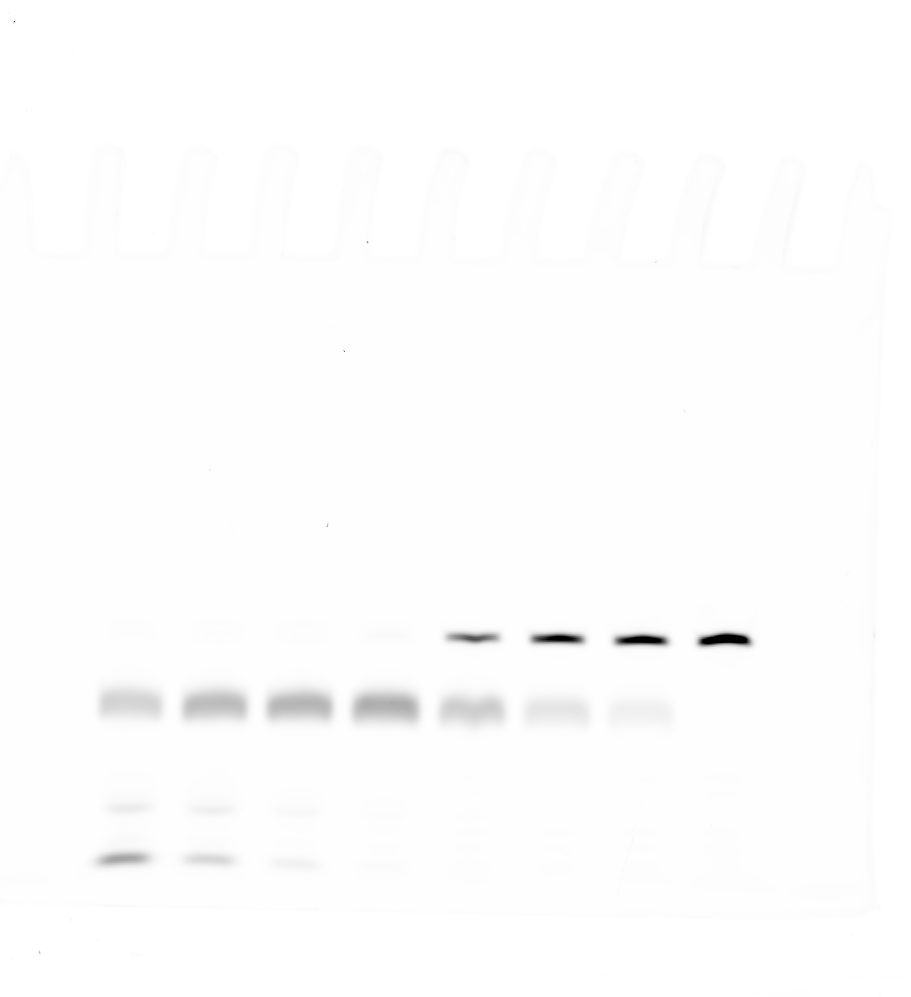

Supplement: Supplementary file 2 — Source Data for Appendix [file EMBJ-41-e109324-s008.zip › EMBOJ-2021-109324_Source_Data_Appendix/Appendix FigS1/FigS1F/FigS1F_MBP-AID_deamination.tif]

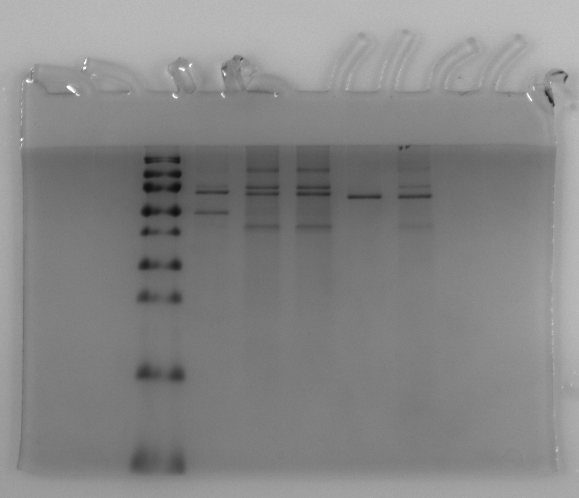

Supplement: Supplementary file 2 — Source Data for Appendix [file EMBJ-41-e109324-s008.zip › EMBOJ-2021-109324_Source_Data_Appendix/Appendix FigS1/FigS1E/FigS1E_MBP-AID variants_CoomassieBlue.tif]

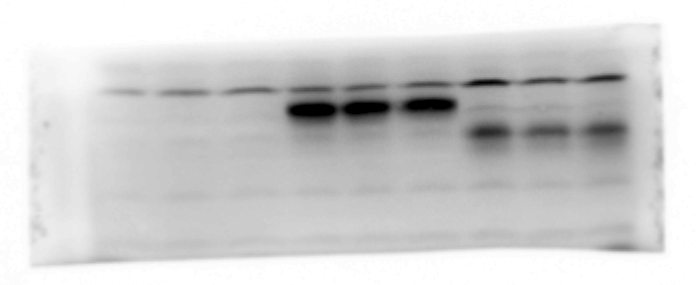

Supplement: Supplementary file 2 — Source Data for Appendix [file EMBJ-41-e109324-s008.zip › EMBOJ-2021-109324_Source_Data_Appendix/Appendix FigS1/FigS1C/FigS1C_CH12F3_AID_WB.tif]

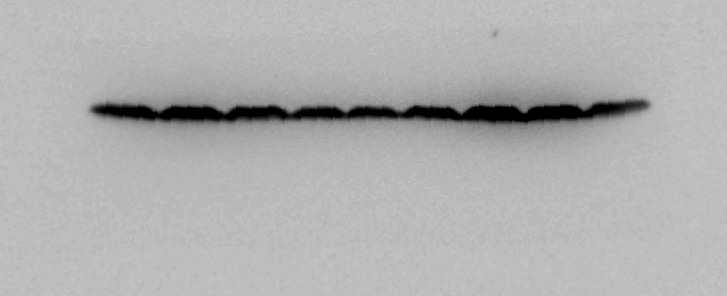

Supplement: Supplementary file 2 — Source Data for Appendix [file EMBJ-41-e109324-s008.zip › EMBOJ-2021-109324_Source_Data_Appendix/Appendix FigS1/FigS1C/FigS1C_CH12F3_tubulin_WB.tif]

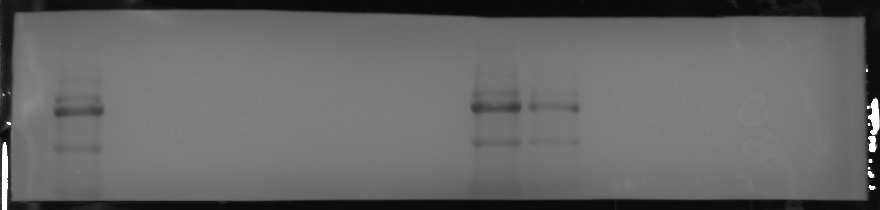

Supplement: Supplementary file 2 — Source Data for Appendix [file EMBJ-41-e109324-s008.zip › EMBOJ-2021-109324_Source_Data_Appendix/Appendix FigS1/FigS1C/FigS1C_CH12F3_tubulin_dilution_marker.tif]

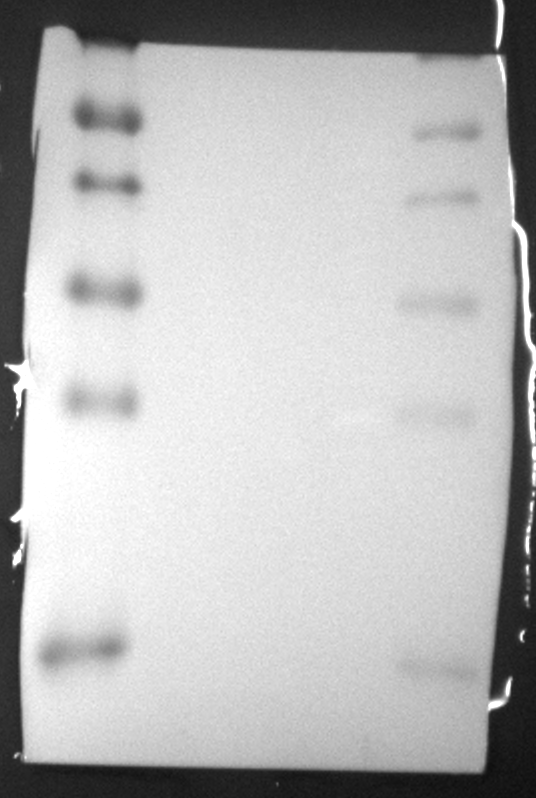

Supplement: Supplementary file 2 — Source Data for Appendix [file EMBJ-41-e109324-s008.zip › EMBOJ-2021-109324_Source_Data_Appendix/Appendix FigS1/FigS1C/FigS1C_primaryB_AID_marker.tif]

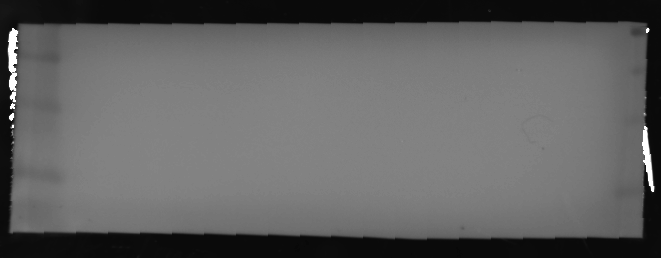

Supplement: Supplementary file 2 — Source Data for Appendix [file EMBJ-41-e109324-s008.zip › EMBOJ-2021-109324_Source_Data_Appendix/Appendix FigS1/FigS1C/FigS1C_CH12F3_tubulin_marker.tif]

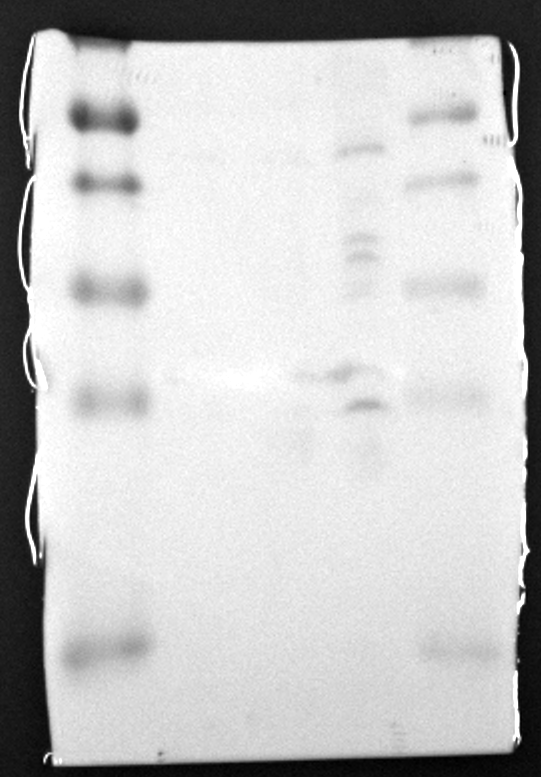

Supplement: Supplementary file 2 — Source Data for Appendix [file EMBJ-41-e109324-s008.zip › EMBOJ-2021-109324_Source_Data_Appendix/Appendix FigS1/FigS1C/FigS1C_primaryB_GFP_marker.tif]

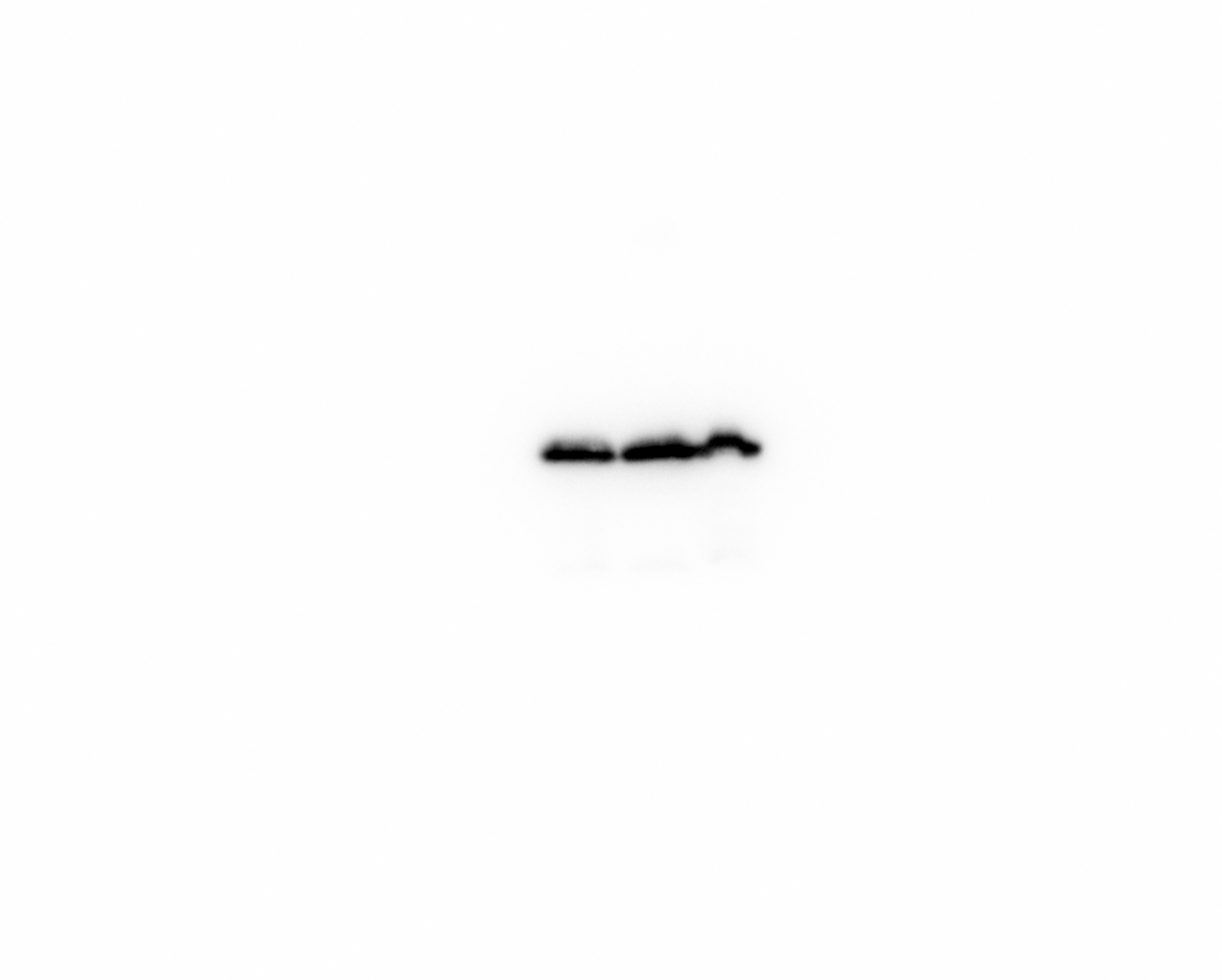

Supplement: Supplementary file 2 — Source Data for Appendix [file EMBJ-41-e109324-s008.zip › EMBOJ-2021-109324_Source_Data_Appendix/Appendix FigS1/FigS1C/FigS1C_primaryB_GFP_WB.tif]

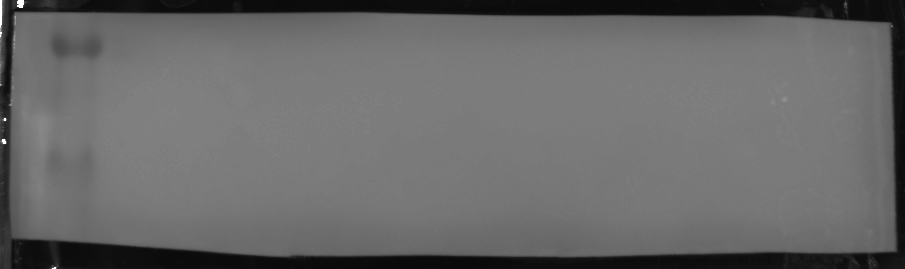

Supplement: Supplementary file 2 — Source Data for Appendix [file EMBJ-41-e109324-s008.zip › EMBOJ-2021-109324_Source_Data_Appendix/Appendix FigS1/FigS1C/FigS1C_CH12F3_AID_dilution_marker.tif]

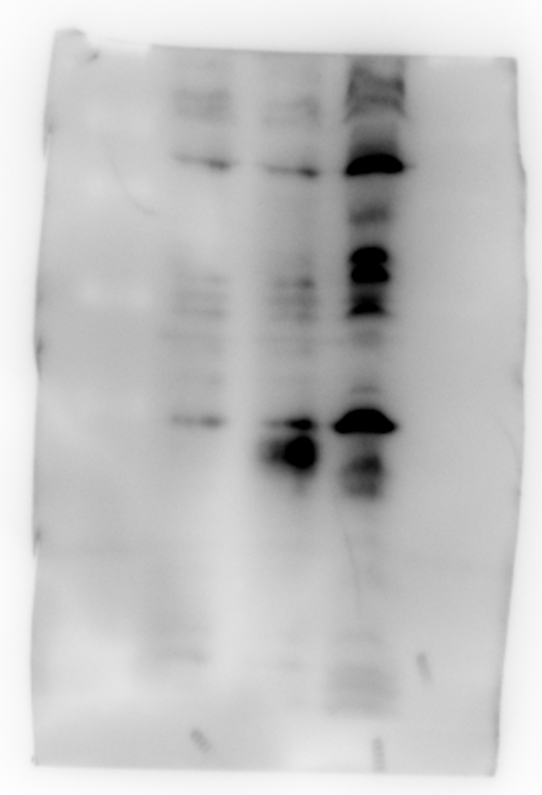

Supplement: Supplementary file 2 — Source Data for Appendix [file EMBJ-41-e109324-s008.zip › EMBOJ-2021-109324_Source_Data_Appendix/Appendix FigS1/FigS1C/FigS1C_primaryB_AID_WB.tif]

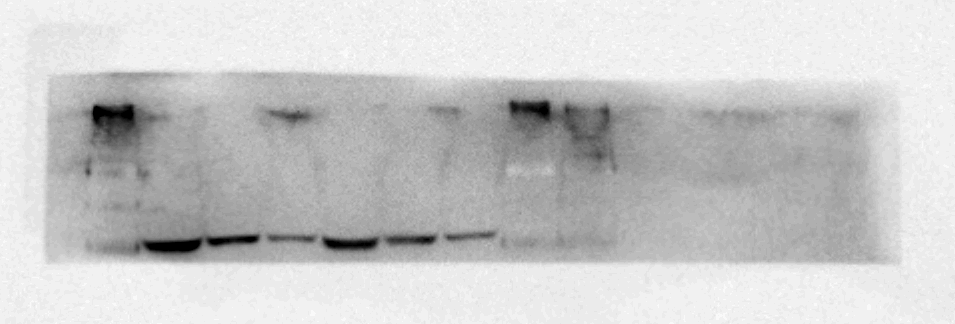

Supplement: Supplementary file 2 — Source Data for Appendix [file EMBJ-41-e109324-s008.zip › EMBOJ-2021-109324_Source_Data_Appendix/Appendix FigS1/FigS1C/FigS1C_CH12F3_tubulin_dilution_WB.tif]

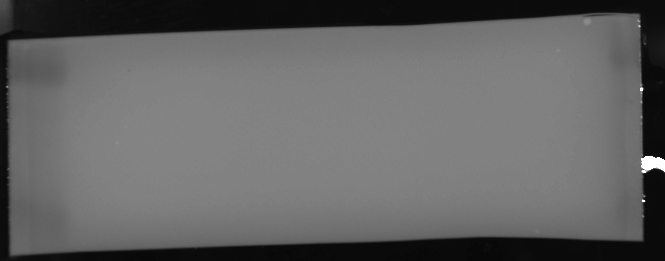

Supplement: Supplementary file 2 — Source Data for Appendix [file EMBJ-41-e109324-s008.zip › EMBOJ-2021-109324_Source_Data_Appendix/Appendix FigS1/FigS1C/FigS1C_CH12F3_AID_marker.tif]

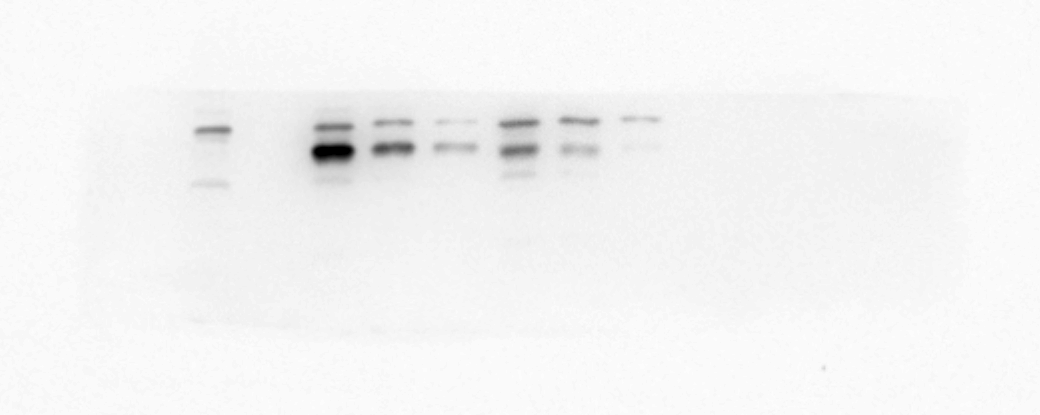

Supplement: Supplementary file 2 — Source Data for Appendix [file EMBJ-41-e109324-s008.zip › EMBOJ-2021-109324_Source_Data_Appendix/Appendix FigS1/FigS1C/FigS1C_CH12F3_AID_dilution_WB.tif]

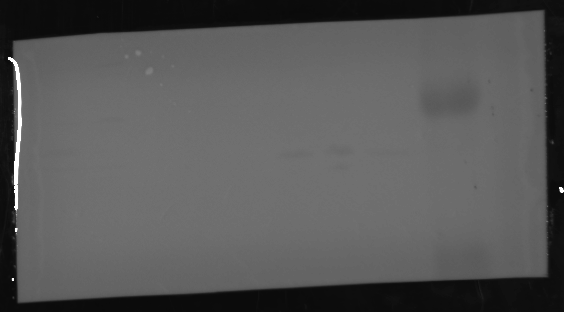

Supplement: Supplementary file 2 — Source Data for Appendix [file EMBJ-41-e109324-s008.zip › EMBOJ-2021-109324_Source_Data_Appendix/Appendix FigS1/FigS1D/FigS1D_AID_Nuc_marker.tif]

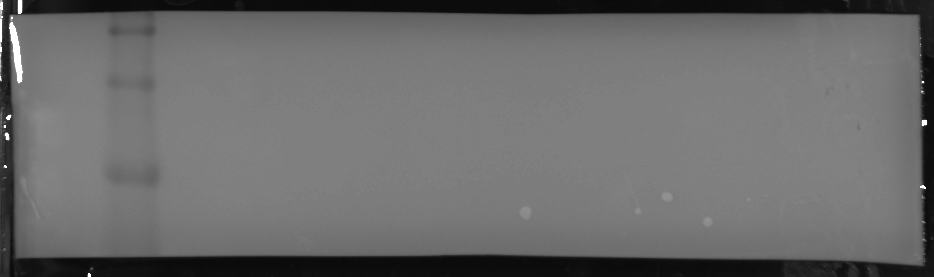

Supplement: Supplementary file 2 — Source Data for Appendix [file EMBJ-41-e109324-s008.zip › EMBOJ-2021-109324_Source_Data_Appendix/Appendix FigS1/FigS1D/FigS1D_tubulin_marker.tif]

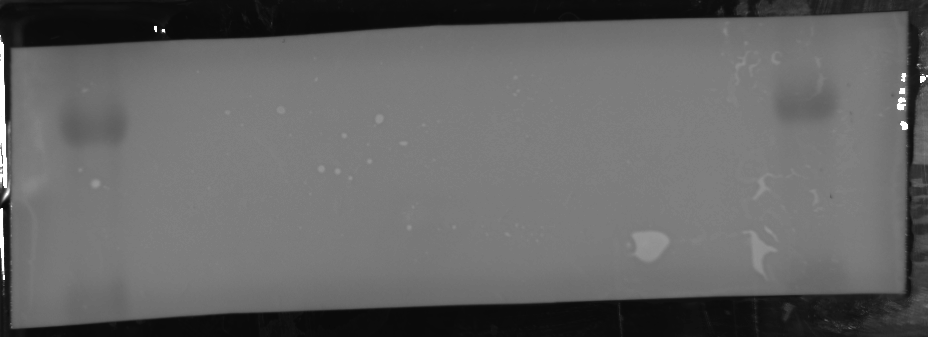

Supplement: Supplementary file 2 — Source Data for Appendix [file EMBJ-41-e109324-s008.zip › EMBOJ-2021-109324_Source_Data_Appendix/Appendix FigS1/FigS1D/FigS1D_AID_marker.tif]

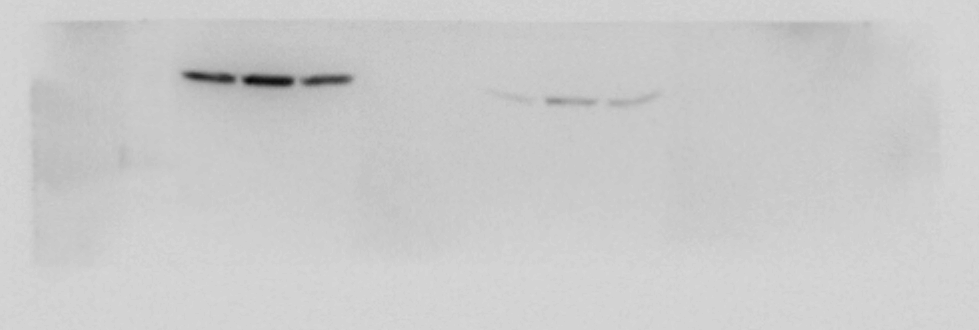

Supplement: Supplementary file 2 — Source Data for Appendix [file EMBJ-41-e109324-s008.zip › EMBOJ-2021-109324_Source_Data_Appendix/Appendix FigS1/FigS1D/FigS1D_tubulin_WB.tif]

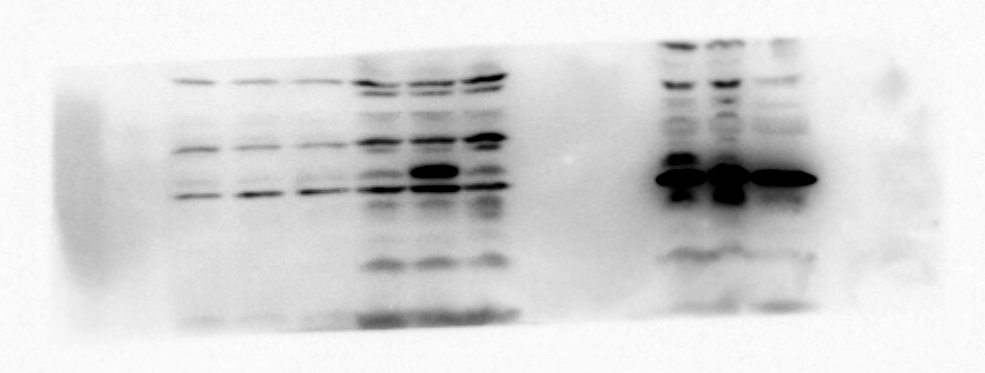

Supplement: Supplementary file 2 — Source Data for Appendix [file EMBJ-41-e109324-s008.zip › EMBOJ-2021-109324_Source_Data_Appendix/Appendix FigS1/FigS1D/FigS1D_AID_WB.tif]

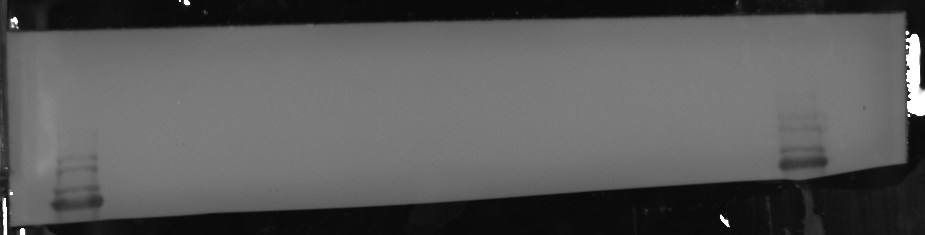

Supplement: Supplementary file 2 — Source Data for Appendix [file EMBJ-41-e109324-s008.zip › EMBOJ-2021-109324_Source_Data_Appendix/Appendix FigS1/FigS1D/FigS1D_PARP1_marker.tif]

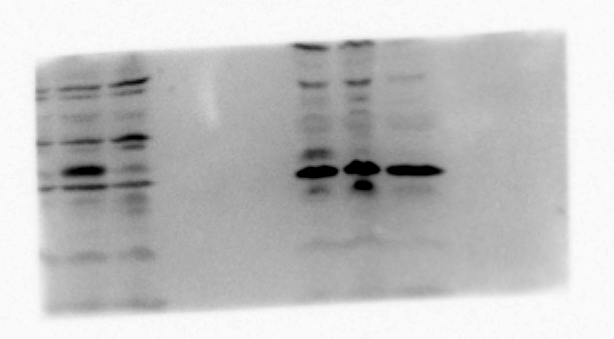

Supplement: Supplementary file 2 — Source Data for Appendix [file EMBJ-41-e109324-s008.zip › EMBOJ-2021-109324_Source_Data_Appendix/Appendix FigS1/FigS1D/FigS1D_AID_Nuc_WB.tif]

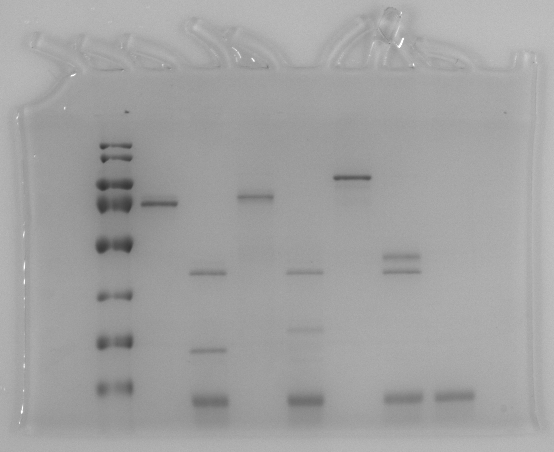

Supplement: Supplementary file 2 — Source Data for Appendix [file EMBJ-41-e109324-s008.zip › EMBOJ-2021-109324_Source_Data_Appendix/Appendix FigS9/FigS9B/FigS9B_purified protein_CoomassieBlue.tif]

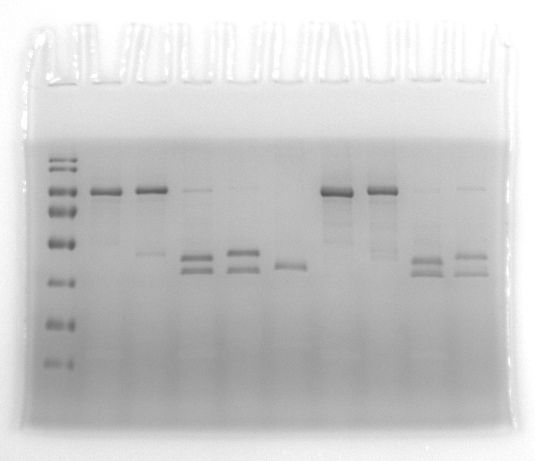

Supplement: Supplementary file 2 — Source Data for Appendix [file EMBJ-41-e109324-s008.zip › EMBOJ-2021-109324_Source_Data_Appendix/Appendix FigS9/FigS9J/FigS9J_MBP-GFP-AIDcry and MBP-GFP-AIDcry+CTT.tif]

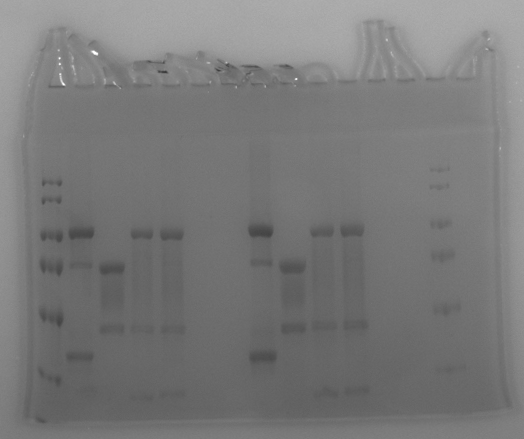

Supplement: Supplementary file 2 — Source Data for Appendix [file EMBJ-41-e109324-s008.zip › EMBOJ-2021-109324_Source_Data_Appendix/Appendix FigS9/FigS9J/FigS9J_MBP-GFP-AID.tif]

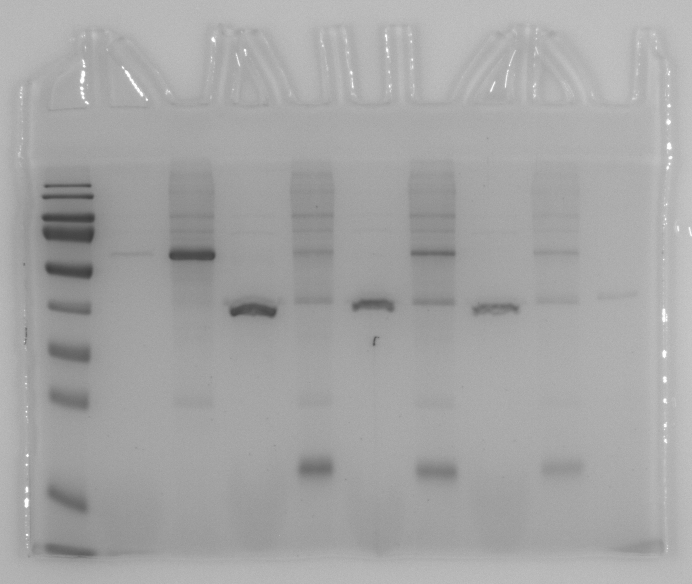

Supplement: Supplementary file 2 — Source Data for Appendix [file EMBJ-41-e109324-s008.zip › EMBOJ-2021-109324_Source_Data_Appendix/Appendix FigS9/FigS9F/FigS9F_PreScission cut_CoomassieBlue.tif]

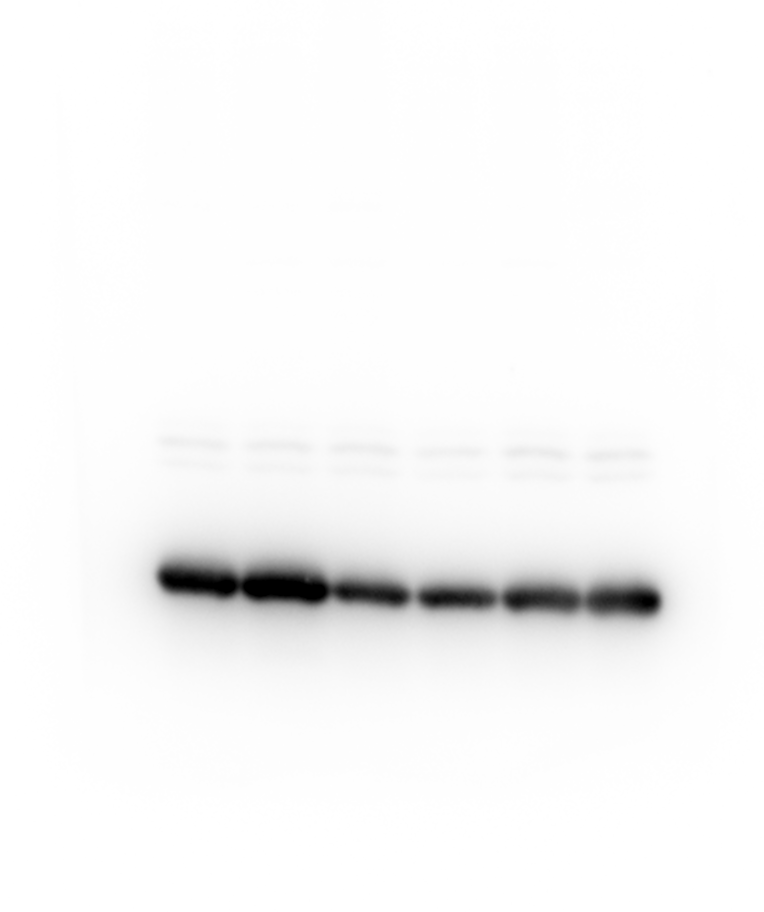

Supplement: Supplementary file 2 — Source Data for Appendix [file EMBJ-41-e109324-s008.zip › EMBOJ-2021-109324_Source_Data_Appendix/Appendix FigS5/FigS5G/FigS5G_GFP_WB.tif]

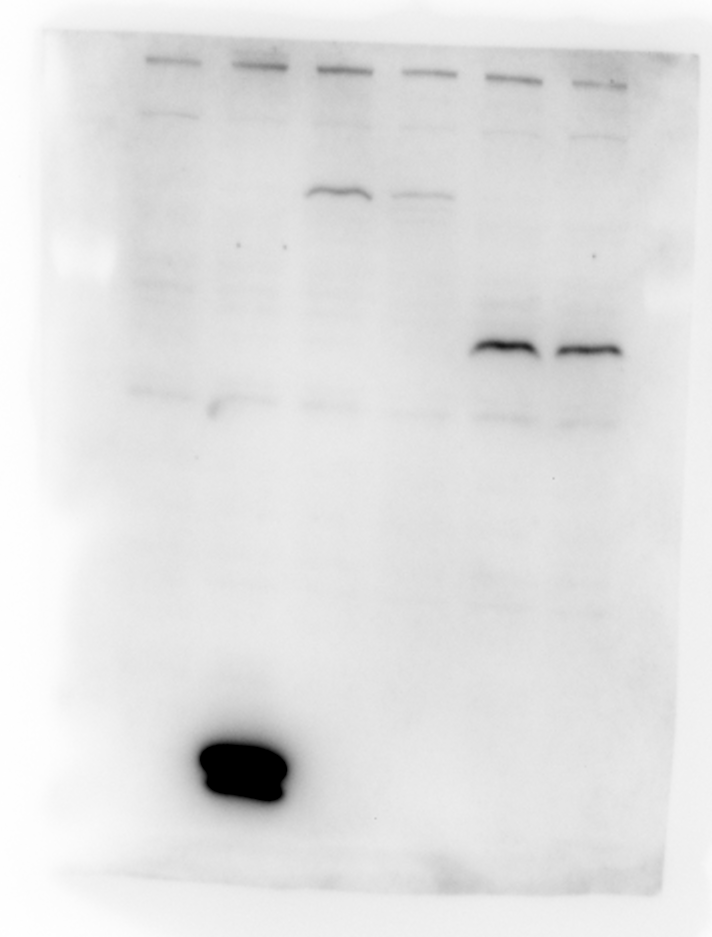

Supplement: Supplementary file 2 — Source Data for Appendix [file EMBJ-41-e109324-s008.zip › EMBOJ-2021-109324_Source_Data_Appendix/Appendix FigS5/FigS5G/FigS5G_AID_WB.tif]

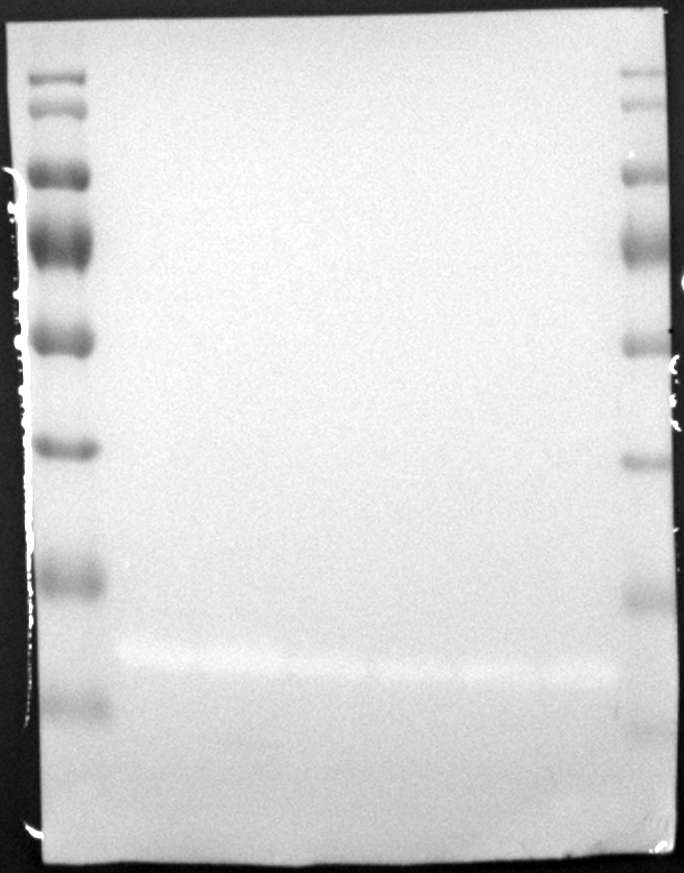

Supplement: Supplementary file 2 — Source Data for Appendix [file EMBJ-41-e109324-s008.zip › EMBOJ-2021-109324_Source_Data_Appendix/Appendix FigS5/FigS5G/FigS5G_GFP_marker.tif]

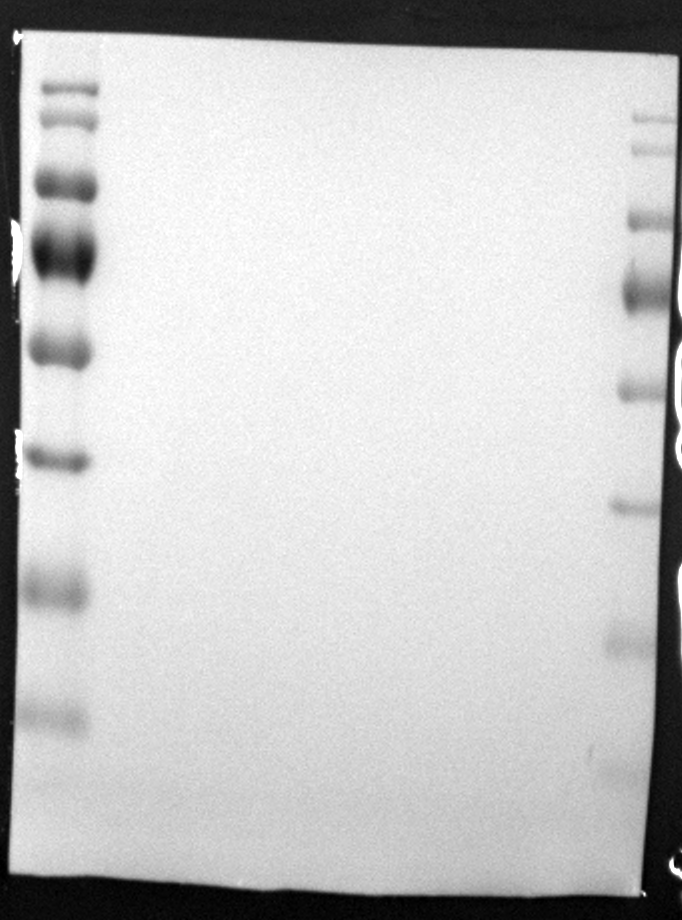

Supplement: Supplementary file 2 — Source Data for Appendix [file EMBJ-41-e109324-s008.zip › EMBOJ-2021-109324_Source_Data_Appendix/Appendix FigS5/FigS5G/FigS5G_AID_marker.tif]

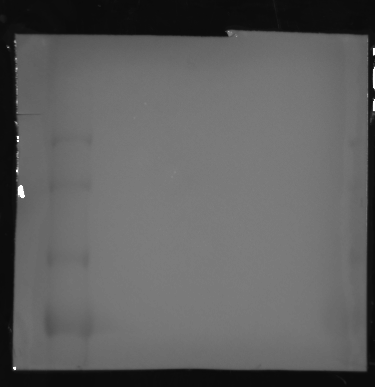

Supplement: Supplementary file 2 — Source Data for Appendix [file EMBJ-41-e109324-s008.zip › EMBOJ-2021-109324_Source_Data_Appendix/Appendix FigS3/FigS3B/FigS3B_Flag_marker.tif]

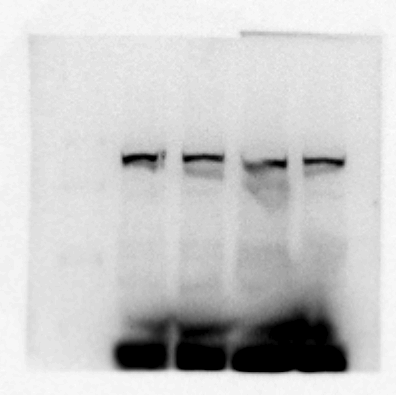

Supplement: Supplementary file 2 — Source Data for Appendix [file EMBJ-41-e109324-s008.zip › EMBOJ-2021-109324_Source_Data_Appendix/Appendix FigS3/FigS3B/FigS3B_Flag_WB.tif]

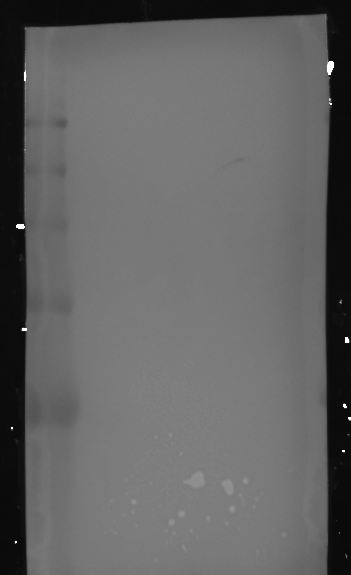

Supplement: Supplementary file 2 — Source Data for Appendix [file EMBJ-41-e109324-s008.zip › EMBOJ-2021-109324_Source_Data_Appendix/Appendix FigS3/FigS3B/FigS3B_MCP_marker.tif]

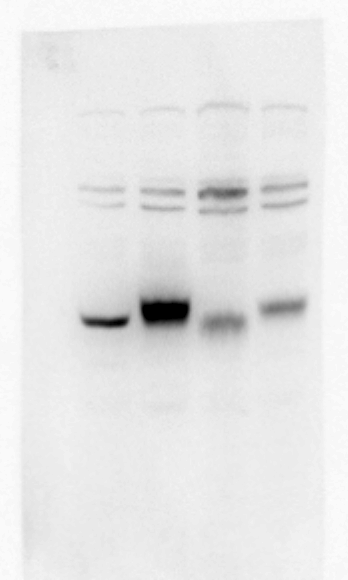

Supplement: Supplementary file 2 — Source Data for Appendix [file EMBJ-41-e109324-s008.zip › EMBOJ-2021-109324_Source_Data_Appendix/Appendix FigS3/FigS3B/FigS3B_MCP_WB.tif]

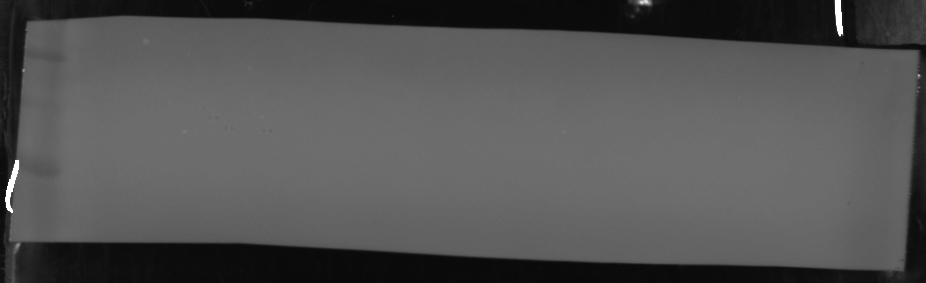

Supplement: Supplementary file 2 — Source Data for Appendix [file EMBJ-41-e109324-s008.zip › EMBOJ-2021-109324_Source_Data_Appendix/Appendix FigS3/FigS3E/FigS3E_tubulin for AID_marker.tif]

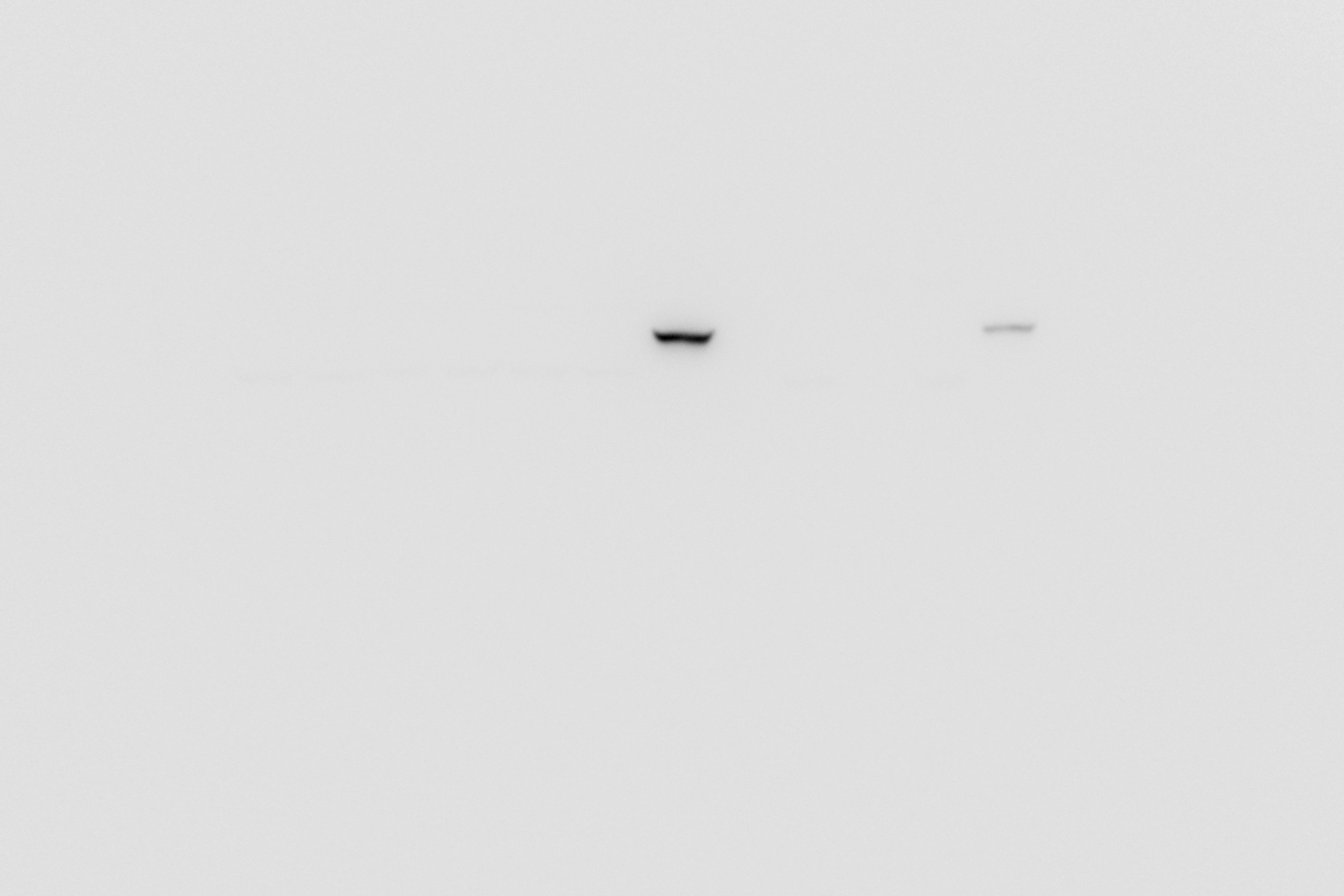

Supplement: Supplementary file 2 — Source Data for Appendix [file EMBJ-41-e109324-s008.zip › EMBOJ-2021-109324_Source_Data_Appendix/Appendix FigS3/FigS3E/FigS3E_Msh2_WB.tif]

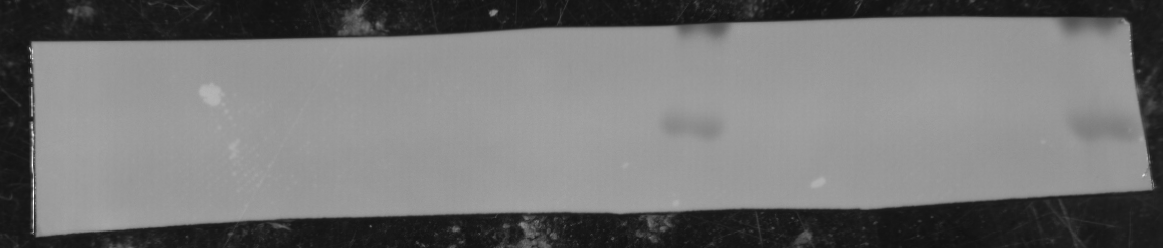

Supplement: Supplementary file 2 — Source Data for Appendix [file EMBJ-41-e109324-s008.zip › EMBOJ-2021-109324_Source_Data_Appendix/Appendix FigS3/FigS3E/FigS3E_tubulin for Msh2_marker.tif]

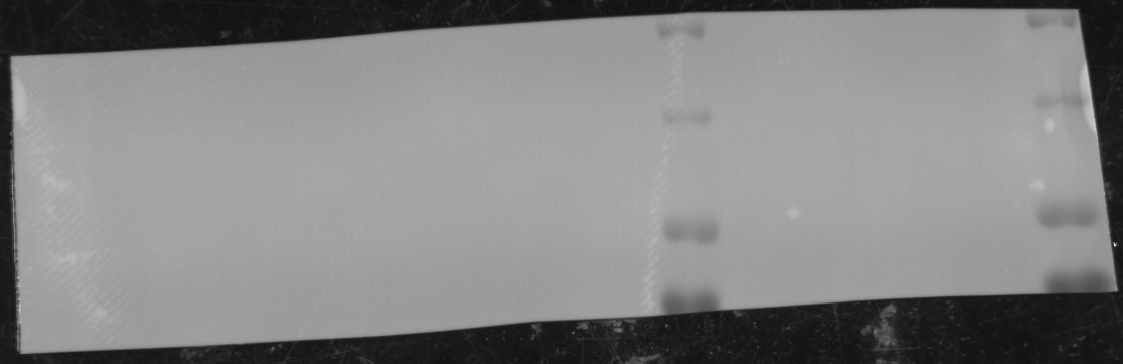

Supplement: Supplementary file 2 — Source Data for Appendix [file EMBJ-41-e109324-s008.zip › EMBOJ-2021-109324_Source_Data_Appendix/Appendix FigS3/FigS3E/FigS3E_Msh2_marker.tif]

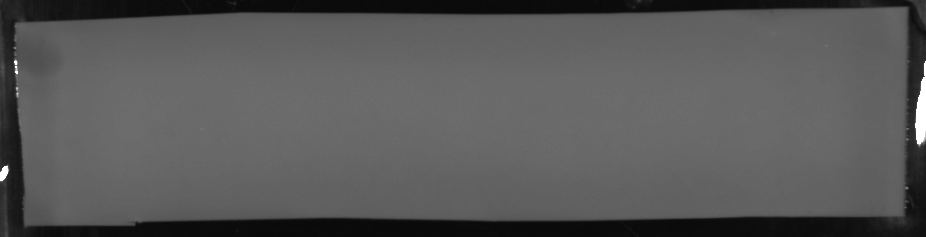

Supplement: Supplementary file 2 — Source Data for Appendix [file EMBJ-41-e109324-s008.zip › EMBOJ-2021-109324_Source_Data_Appendix/Appendix FigS3/FigS3E/FigS3E_AID_marker.tif]

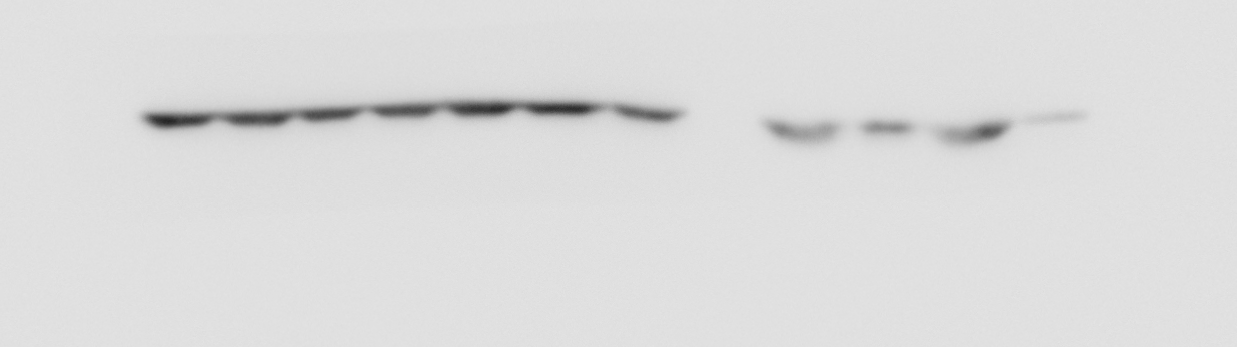

Supplement: Supplementary file 2 — Source Data for Appendix [file EMBJ-41-e109324-s008.zip › EMBOJ-2021-109324_Source_Data_Appendix/Appendix FigS3/FigS3E/FigS3E_tubulin for Msh2_WB.tif]

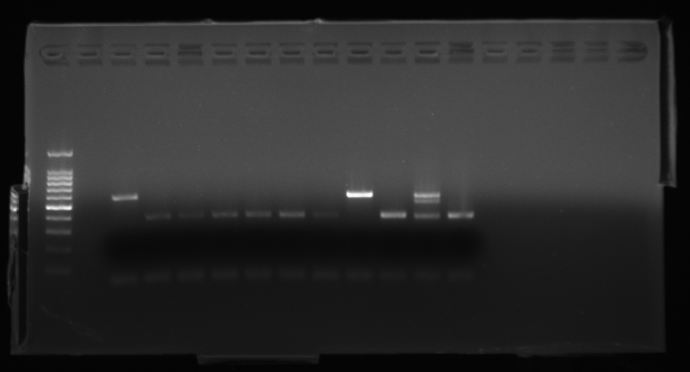

Supplement: Supplementary file 2 — Source Data for Appendix [file EMBJ-41-e109324-s008.zip › EMBOJ-2021-109324_Source_Data_Appendix/Appendix FigS3/FigS3E/FigS3E_Ung.tif]

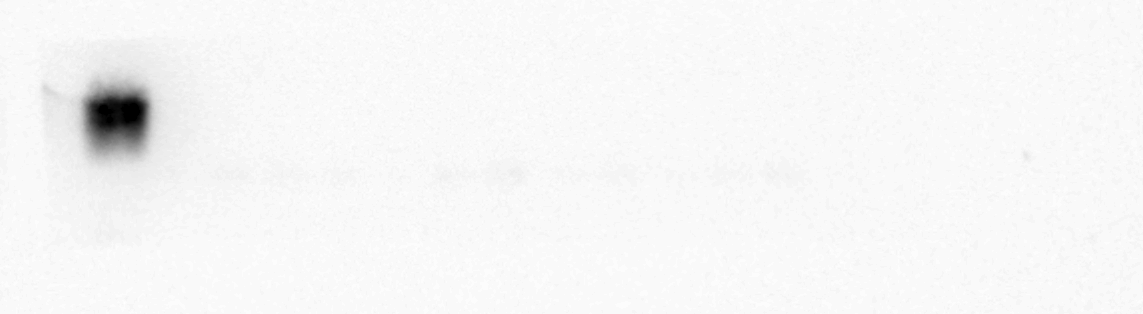

Supplement: Supplementary file 2 — Source Data for Appendix [file EMBJ-41-e109324-s008.zip › EMBOJ-2021-109324_Source_Data_Appendix/Appendix FigS3/FigS3E/FigS3E_AID_WB.tif]

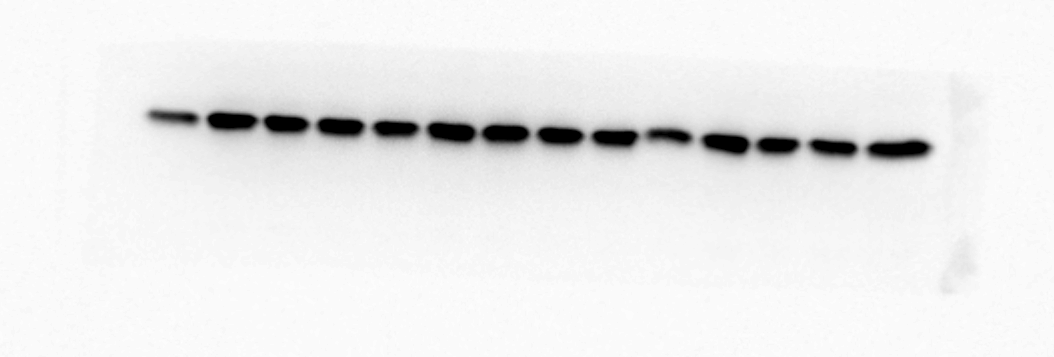

Supplement: Supplementary file 2 — Source Data for Appendix [file EMBJ-41-e109324-s008.zip › EMBOJ-2021-109324_Source_Data_Appendix/Appendix FigS3/FigS3E/FigS3E_tubulin for AID_WB.tif]

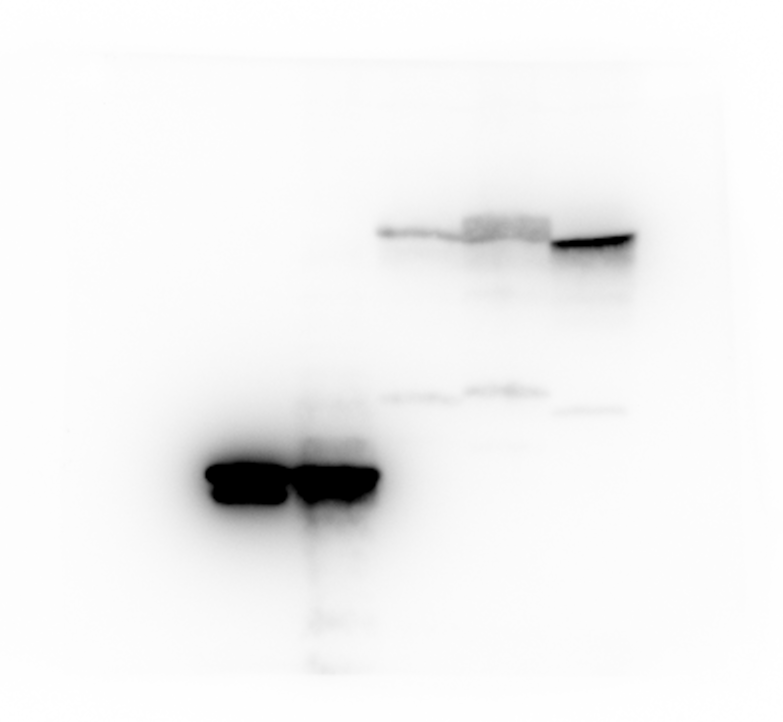

Supplement: Supplementary file 2 — Source Data for Appendix [file EMBJ-41-e109324-s008.zip › EMBOJ-2021-109324_Source_Data_Appendix/Appendix FigS3/FigS3C/FigS3C_AID_WB.tif]

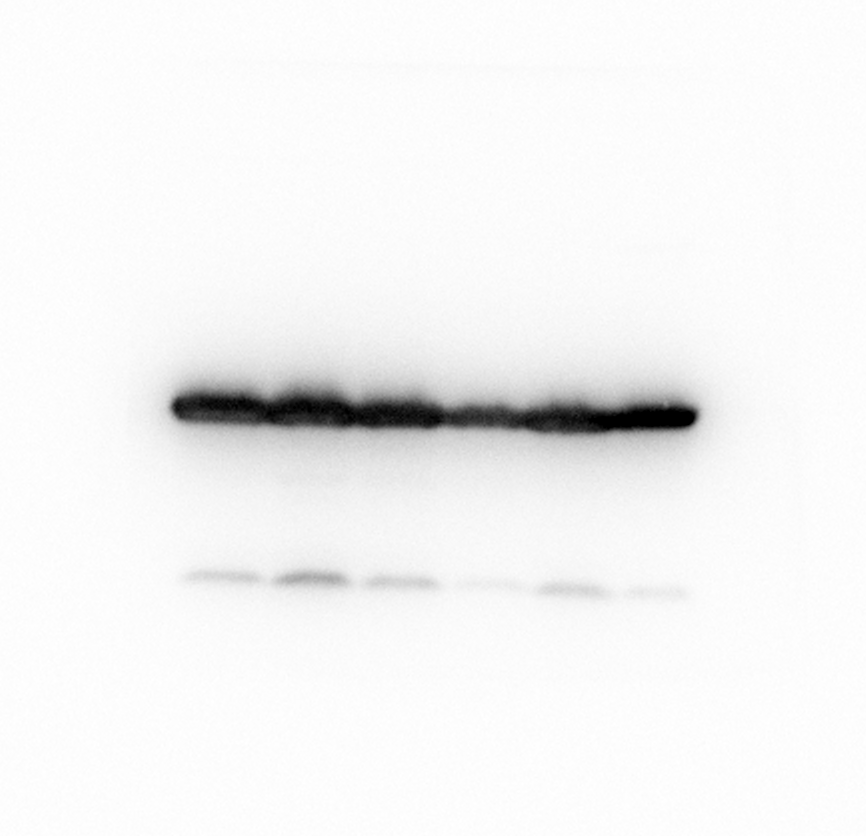

Supplement: Supplementary file 2 — Source Data for Appendix [file EMBJ-41-e109324-s008.zip › EMBOJ-2021-109324_Source_Data_Appendix/Appendix FigS3/FigS3C/FigS3C_GFP_WB.tif]

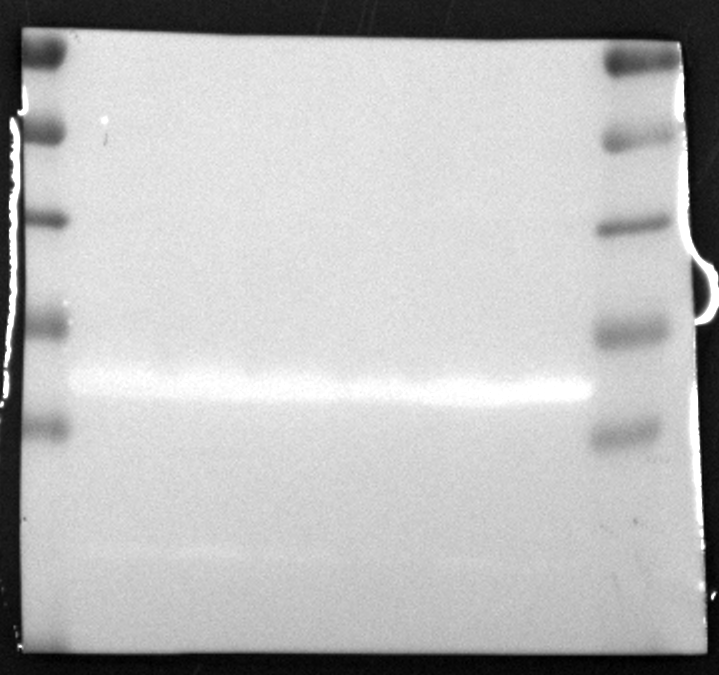

Supplement: Supplementary file 2 — Source Data for Appendix [file EMBJ-41-e109324-s008.zip › EMBOJ-2021-109324_Source_Data_Appendix/Appendix FigS3/FigS3C/FigS3C_GFP_marker.tif]

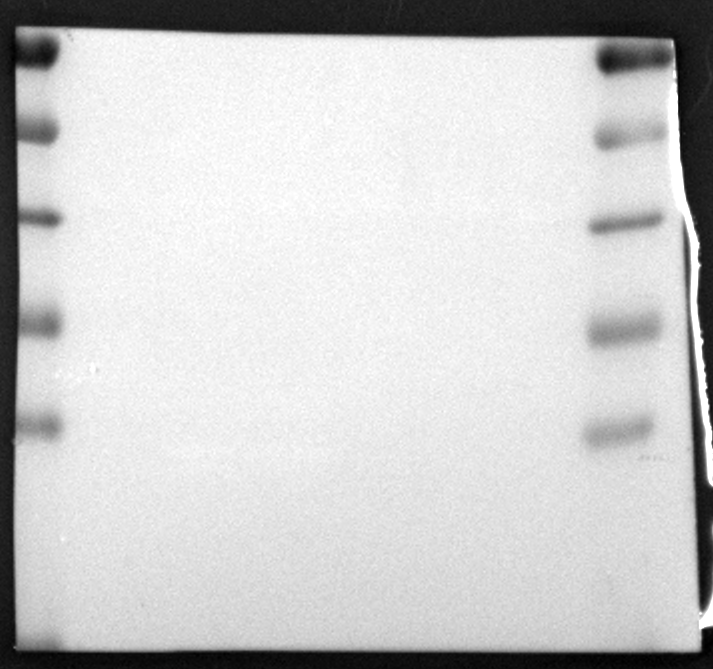

Supplement: Supplementary file 2 — Source Data for Appendix [file EMBJ-41-e109324-s008.zip › EMBOJ-2021-109324_Source_Data_Appendix/Appendix FigS3/FigS3C/FigS3C_AID_marker.tif]

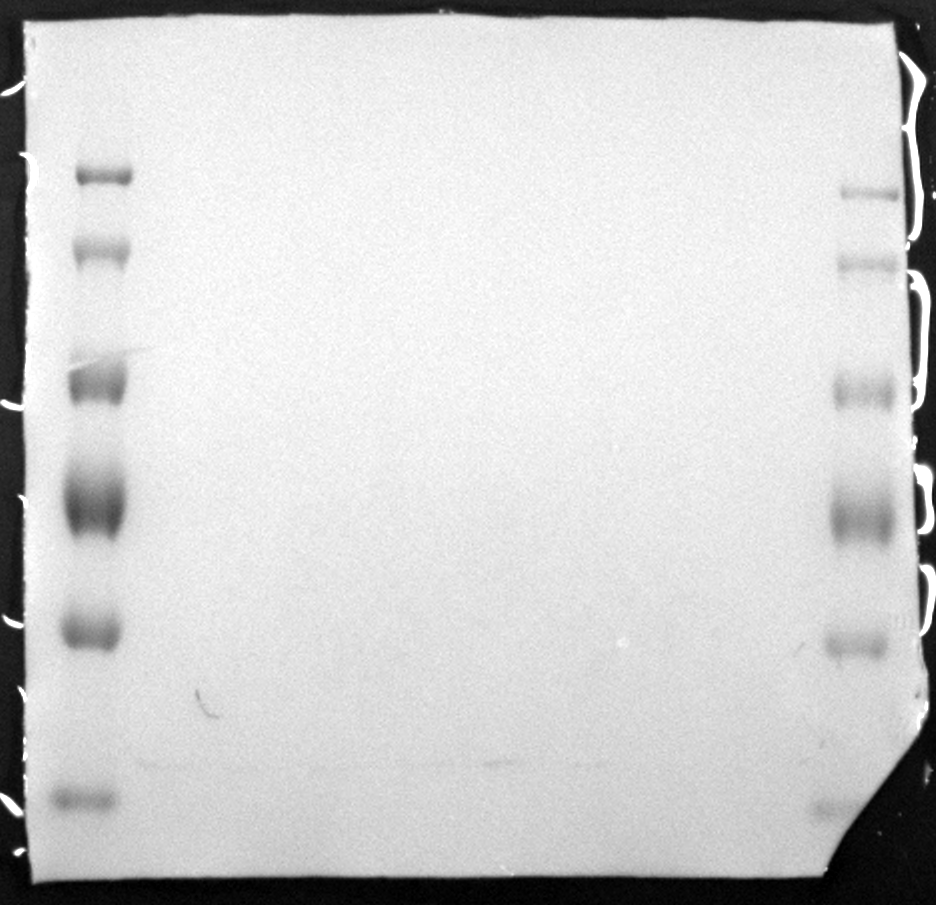

Supplement: Supplementary file 2 — Source Data for Appendix [file EMBJ-41-e109324-s008.zip › EMBOJ-2021-109324_Source_Data_Appendix/Appendix FigS3/FigS3G/FigS3G_tubulin_marker.tif]

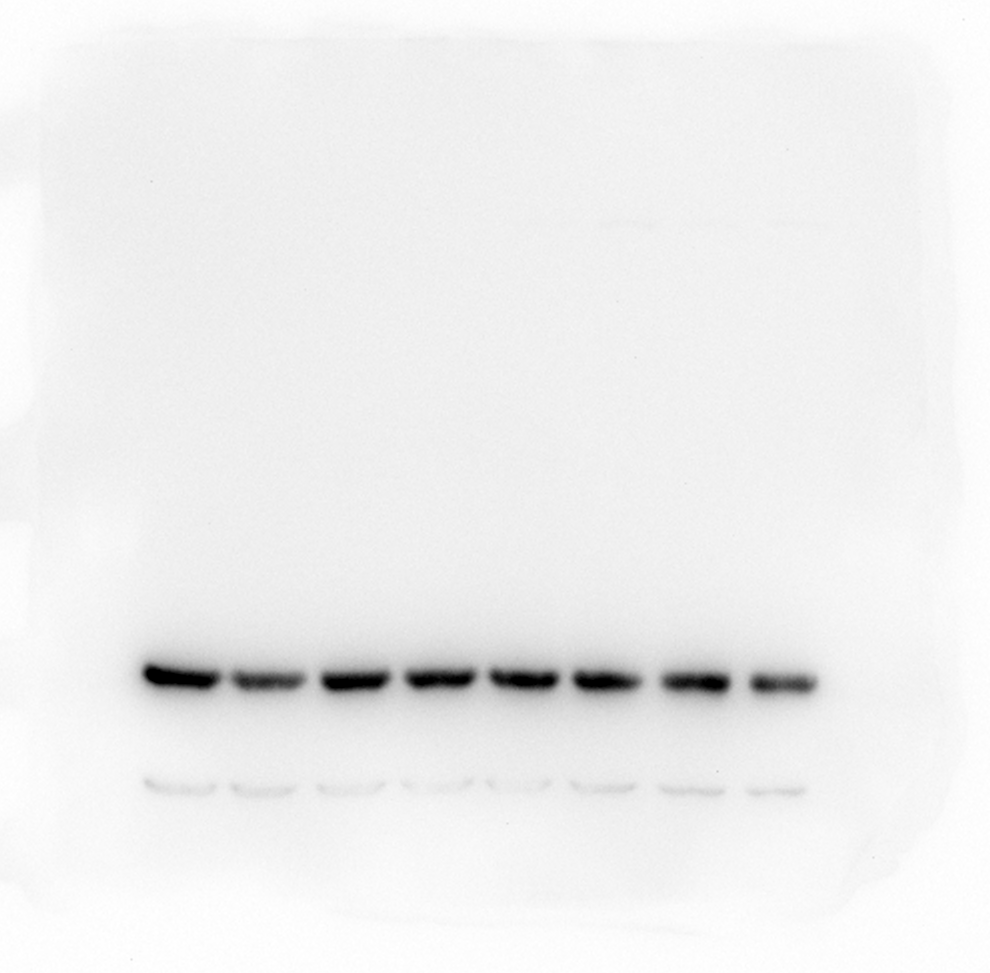

Supplement: Supplementary file 2 — Source Data for Appendix [file EMBJ-41-e109324-s008.zip › EMBOJ-2021-109324_Source_Data_Appendix/Appendix FigS3/FigS3G/FigS3G_tubulin_WB.tif]

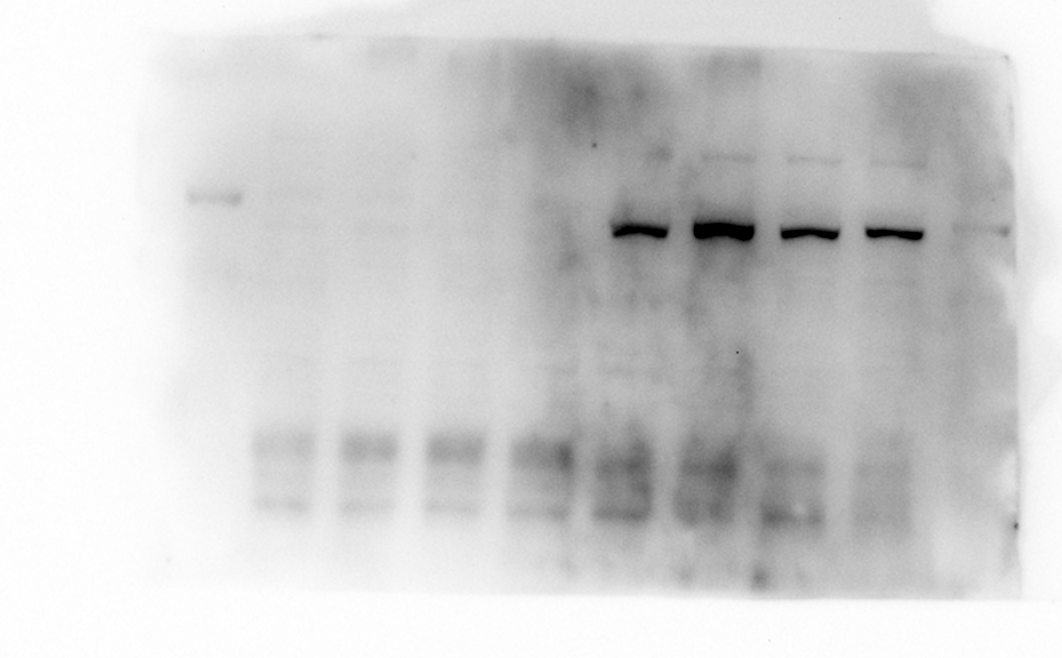

Supplement: Supplementary file 2 — Source Data for Appendix [file EMBJ-41-e109324-s008.zip › EMBOJ-2021-109324_Source_Data_Appendix/Appendix FigS3/FigS3G/FigS3G_flag_WB.tif]

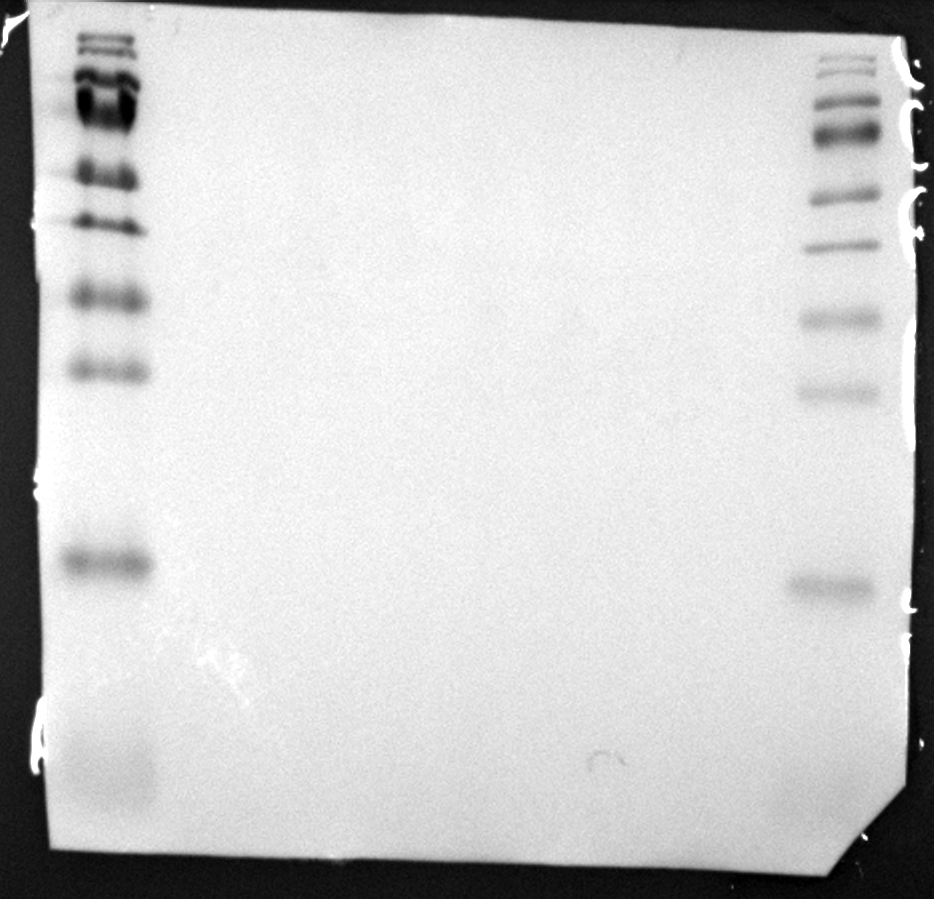

Supplement: Supplementary file 2 — Source Data for Appendix [file EMBJ-41-e109324-s008.zip › EMBOJ-2021-109324_Source_Data_Appendix/Appendix FigS3/FigS3G/FigS3G_MCP_marker.tif]

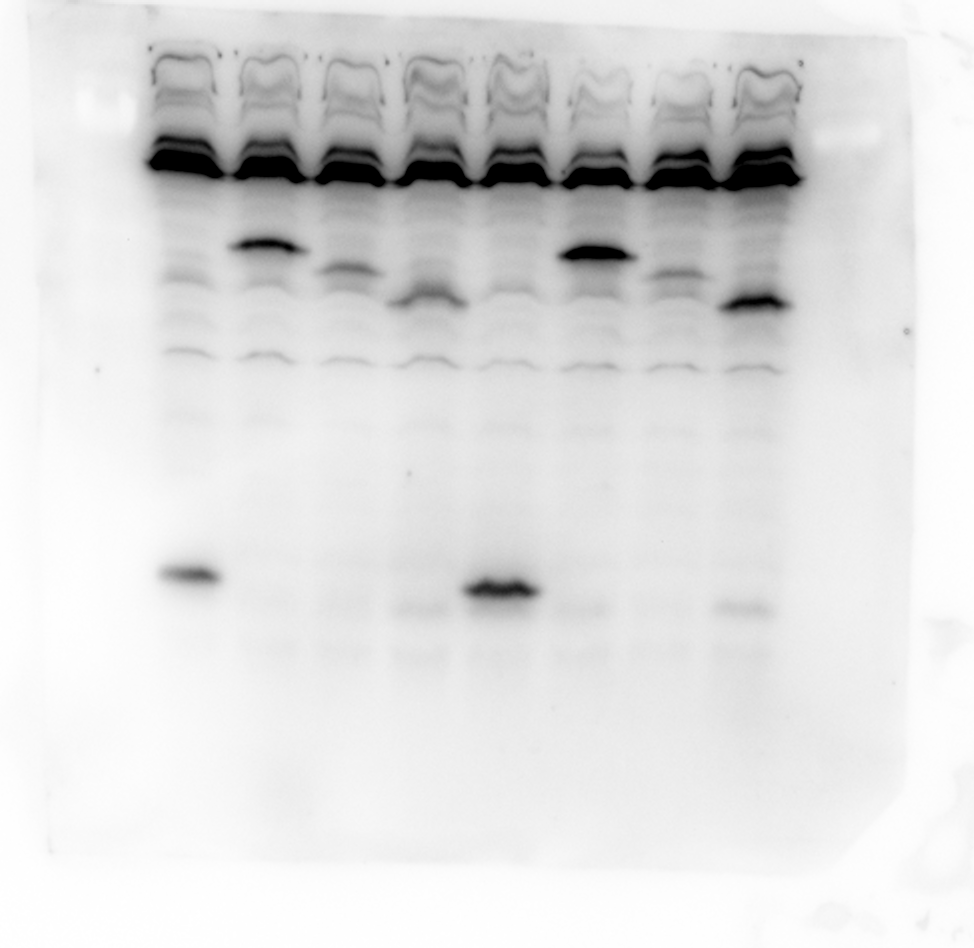

Supplement: Supplementary file 2 — Source Data for Appendix [file EMBJ-41-e109324-s008.zip › EMBOJ-2021-109324_Source_Data_Appendix/Appendix FigS3/FigS3G/FigS3G_MCP_WB.tif]

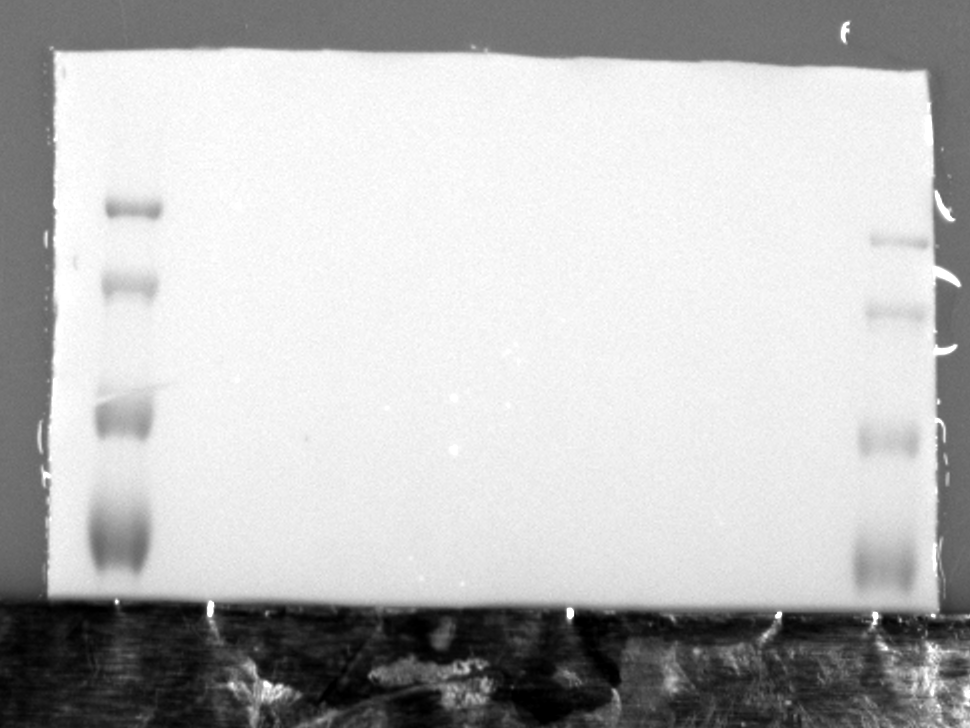

Supplement: Supplementary file 2 — Source Data for Appendix [file EMBJ-41-e109324-s008.zip › EMBOJ-2021-109324_Source_Data_Appendix/Appendix FigS3/FigS3G/FigS3G_flag_marker.tif]

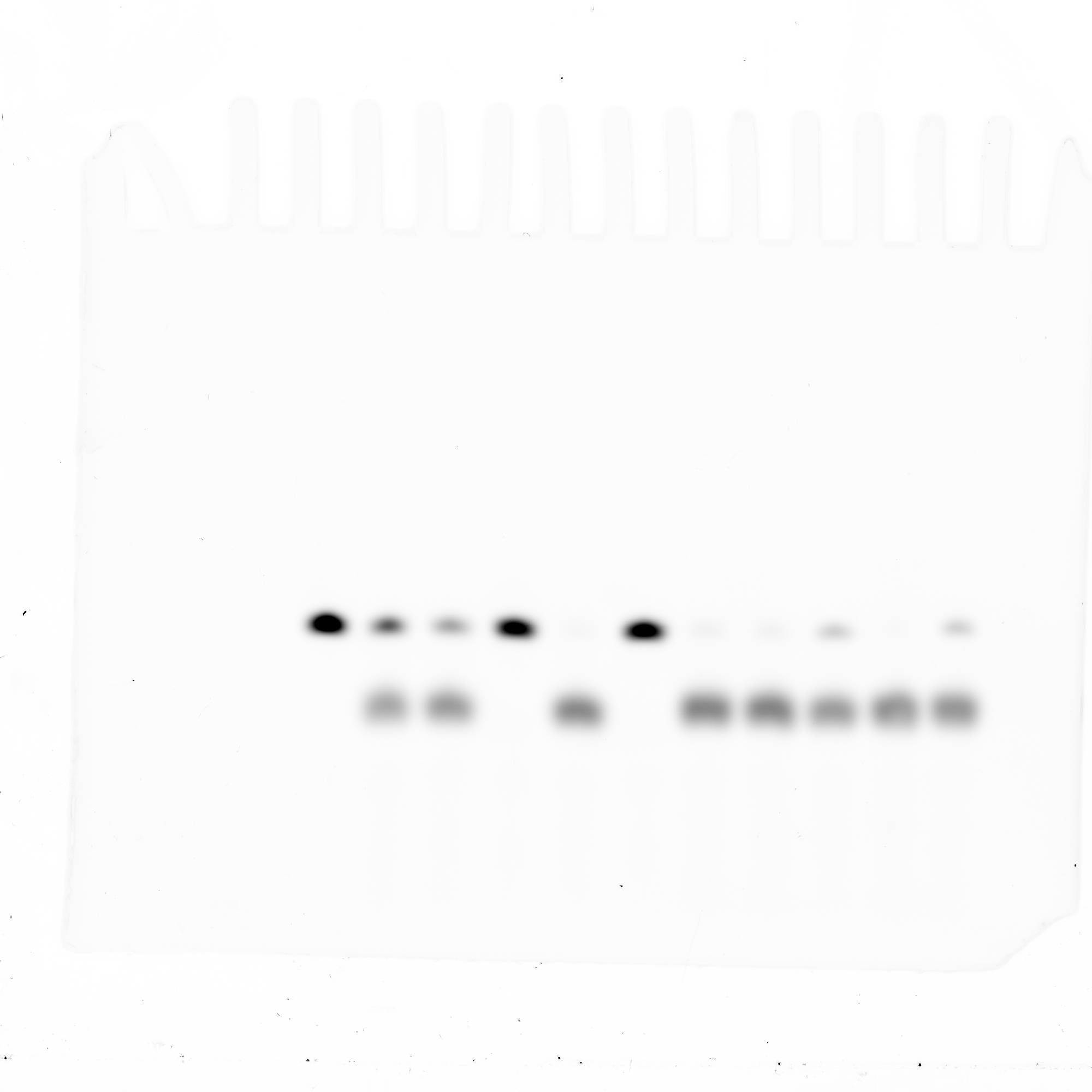

Supplement: Supplementary file 8 — Source Data for Figure 6 [file EMBJ-41-e109324-s002.zip › EMBOJ-2021-109324_SourceData_Fig6/Fig6A/Fig6A_deamination.tif]
