# Supplementary material for: C‐terminal deletion‐induced condensation sequesters AID from IgH targets in immunodeficiency
Source: EMBO J. 2022 Apr 26;41(11):e109324. doi: 10.15252/embj.2021109324 (PMC9156971; doi:10.15252/embj.2021109324)
Supplement: Supplementary file 9 [file EMBJ-41-e109324-s004.pdf]

THE EMBO JOURNAL

C-terminal deletion-induced condensation sequesters AID from IgH targets in immunodeficiency

Feilong Meng, Jiazhi Hu, Xia Xie, Tingting Gan, Bing Rao, Weiwei Zhang, Rohit Panchakshari, Dingpeng Yang, Xiong Ji, Yu Cao, and Frederick Alt
DOI: 10.15252/embj.2021109324

Corresponding author(s): Feilong Meng (Feilong.Meng@sibcb.ac.cn) , Jiazhi Hu (hujz@pku.edu.cn)

Review Timeline:

Submission Date:	30th Jul 21
Editorial Decision:	8th Sep 21
Resubmission:	31st Jan 22
Author pre-consultation:	15th Mar 22
Author response:	23rd Mar 22
Editorial Decision:	1st Apr 22
Revision Received:	7th Apr 22
Accepted:	8th Apr 22

Editor: Hartmut Vodermaier

Transaction Report:

(Note: With the exception of the correction of typographical or spelling errors that could be a source of ambiguity, letters and reports are not edited. Depending on transfer agreements, referee reports obtained elsewhere may or may not be included in this compilation. Referee reports are anonymous unless the Referee chooses to sign their reports.)

1st Editorial Decision 8th Sep 2021

Thank you again for submitting your manuscript on condensation/phase-separation of mutant AID to The EMBO Journal. I am sorry for the delay in its external review over the summer vacation period, but we have now finally received all reports from three expert referees, copied below for your information. Based on these reports and further discussions with/between the reviewers, we unfortunately had to conclude that the study is presently not a sufficiently strong candidate for publication in our journal. As you will see, while referee 2 is not convinced about the wider significance of understanding C-terminal AID mutants, both referees 1 and 3 consider this question important in principle, and your current findings potentially interesting. However, they raise the overriding concern that it remains unclear whether AID condensation is the cause, or just another consequence, of its defective nuclear targeting/function or binding to interaction partners; moreover, especially referee 1 indicates that your present data do not provide sufficiently definitive evidence in support of the trapping model, nor good insight into the mechanism of how AID mutants are concentrated in nucleoli or other nuclear compartments. Combined with the more general concern by referee 2 that there is currently no evidence for phase separation also being involved in the biology/regulation of wild-type AID, I am afraid these issues preclude us from offering publication of this work in The EMBO Journal at the present stage.

I am sorry that the reports do not allow me to come to a more positive conclusion on this occasion, but hope that you will nevertheless find the detailed comments and suggestions from our referees helpful for considering your further proceedings with this study. Thank you once again for having had the opportunity to consider this work for publication.

****

Referee #1:

AID incudes DNA lesions and plays an important role in class switch recombination and somatic hypermutation during B cell development. Xie and colleagues discovered that a C-terminal deletion mutant of AID shows abnormal nucleus localization, accompanied by a loss of function in triggering class switch recombination. The authors proposed that the mutant has increased condensation properties, which, in a heterozygous background, traps the wild-type protein from approaching the target sites. This study highlighted the functional importance of intranuclear localization of AID and resolved a puzzling observation that the C-terminal deletion mutant has increased nuclear localization but decreased activity. The data quality is generally high. However, data supporting the trapping model need to be strengthened. In addition, the mechanism of how AID mutants are concentrated in nucleoli has not been elucidated.

Major points:

1. To prove the model that AID R190X traps AID WT in condensates and prevents WT approaching target sites, it is critical to quantify the nuclear fluoresce intensity of WT AID in the presence and absence of AID R190X. In Fig. 6A and 6B, it looks like in AID R190X cells, AID WT has a similar nuclear intensity (outside the condensates region), if not higher, than the one without R190X. If this is true by quantification, it will challenge the trapping model. An explanation or alternative mechanism will be needed.

2. Elegant optogenetic tools were implemented to show AID Delta C has a higher tendency to form condensates than AID WT in the nucleus. This is very nice. However, there are some technical caveats on the in vitro biochemistry showing AID

condensation. The current in vitro data did not strengthen the conclusion. For reasons elaborated below, I would suggest either improving the in vitro condensation assays or removing this part and acknowledging the difficulty in obtaining high quantity of AID WT and R190X.
1) AID-Cry, but not AID WT and AID R190X, was used in the in vitro condensation assays. What is the physiological or pathological relevance of AID-Cry? 2) All the in vitro condensation assays included 10% PEG in the buffer, which is problematic because PEG can promote non-specific condensation as a crowding reagent. It is better to test condensation without PEG. 3) MBP increases the solubility of protein, which also inhibits condensation in general. Using an MBP-tagged protein in the condensation assay would dramatically affect the protein-of-interest's ability to form condensates.

3. The physical properties of R190X condensates formed in cells needs to be better characterized (Figure 3A, 5A and 6A): 1) FRAP performed both in condensates and the surrounding nuclear compartment to determine if condensation reduces R190X mobility; 2) Quantifying the fold-enrichment of R190X in condensates as compared to the surrounding area.

4. AID R190X condensates form on nucleoli or other nuclear compartments (Fig 3A and S4B). This strongly suggests AID 190X does not form condensates by itself. Instead, it is recruited to other pre-formed nuclear condensates. This mechanism needs to be clearly acknowledged and the candidate proteins in nucleoli that recruit AID mutants should be discussed.

Minor points
1. The experiments of testing the chimeric protein AID R190X-AID E58Q is elegantly designed. Is this chimeric protein enriched in specific nuclear localization?

2. Can the author comment on how the truncated C-terminus in AID R190X helps to recruit AID to the target sites?

3. The authors proposed that the frequent infiltration in nuclear compartment of AID delta C might serve as a way to avoid aberrant AID damages. This is a very intriguing point. Is it possible to design experiments to test this idea, e.g., by using the mutants that have attenuated condensation properties?

Referee #2:

Xie et al.
C-terminal deletion-induced condensation sequesters AID from IgH targets in immunodeficiency

In humans, the cytidine deaminase AID drives somatic diversification of antibody genes during the secondary immune response. It targets the V regions of the Ig loci causing hypermutation, allowing affinity maturation of antibody specificity, and it also causes class switch recombination in which the constant region is switch from mu (IgM) to one of the downstream isotypes. Loss of AID activity leads to immunodeficiency characterised by poor affinity maturation and high levels of unswitched IgM antibody. Most mutations in AID affect its ability to deaminate C effectively and are recessive. However, a rare class of mutations results in loss of the C-terminus of the protein and behave in a dominant manner.
The reason for this has been unclear although a number of theories have been advanced, related to proposed functions of the C terminus of AID.

The current manuscript provides a plausible explanation for why loss of the AID C-terminus acts as a dominant negative mutation by proposing that in the absence of the C-terminus the protein form condensates and can be sequestered into membraneless compartments within the nucleus, and indeed can bring wild type AID with it, reducing the activity of the enzyme at the Ig loci.

The experiments to support this hypothesis are well performed, well controlled and clearly presented. The paper uses a wealth of up-to-date approaches. I have only some minor comments and questions about the data that is presented. However, I am less convinced about the extent of the advance and its general interest, rather than interest to those in the field, in terms of understanding the role of the C terminus of AID in the function of the protein. The work nicely explains why these rare C-terminus mutants act as dominant negatives, but it does not address whether phase separation is a part of the normal biology and/or regulation of AID nor how the C terminus prevents inappropriate sequestration of the protein in existing membraneless organelles. Further insight into these points could significantly raise the significance of the study.

Specific points & questions:

Does the MBP tag affect the phase separation behaviour of AID in the in vitro assays shown in Figure 4?

Do the mutations (shown expressed in CH12 in Fig 5G) that reduce droplet formation by the deltaC mutant also rescue any of the lost CSR-inducing activity in CH12?

There are a few places where the English needs some attention and a few typos need correction (page numbers would have

been helpful):

Abstract, line 5: deficiency should be deficient

Section heading: AIDdeltaC shows a defect in targeting ... add 'a'

Same section first line: not sure what 'The defect of the arousing DNA repair ...' means.

Section AIDdeltaC is frequently enriched ... line 2: experimental

Referee #3:

In this manuscript Xie and coworkers have explored the dominant negative phenotype of AID truncated at the C-terminus. This is a worthy effort since the molecular mechanism of why truncated AID (t-AID) fails to support CSR has been an open question for many years. The authors have applied a long series of assays to unravel this question. They have confirmed the work of others shows that t-AID supports deamination in vitro but not CSR in vivo. They have engineered a CRISPR model to drag t-AID to its template and show that it can mediate CSR. They confirm the work of others showing that t-AID diffuses from the cytoplasm to the nucleus. In new studies they demonstrate that when t-AID is in the nucleus it can form molecular condensates. They go on to explore the nature of the AID condensates and suggest that the presence of t-AID can alter the localization of full length AID and move it into the nucleolus and can force AID to also form condensates. They wish to conclude that the dominant negative phenotype of AID is directly correlated to the formation of condensates in the nucleus. This manuscript reports many studies and much data. Unfortunately, the presentation of the data is very frequently unclear and the writing is replete with grammatical and syntactical mistakes. More importantly the manuscript is very poorly edited and spends space on confirming the results of others and not enough effort or focus on clearly explaining their new studies and the assays they perform. Although there may be some important and novel findings it is difficult to assent to the authors conclusions as in many instances that data are unclear or lacking.

A partial set of examples of how the manuscript could be streamlined follow: Much of Figure 1A-E and Suppl. figure 1 show that previously described AID mutants fail to support CSR but have deamination activity. Not much novel information is provided and should be moved to Suppl. Figures. The new studies begin with Fig. 1F-H and go together with Figure 2 A-C.
Figure 3A,B provide little new information and should be moved to Suppl. Figures. The experiment shown in Figure 3C is relevant to the new studies. Figure 4A-D is weak because there is no WT control and therefore should be moved to Suppl. Figures. Figure 4 E is new and interesting information and could be amalgamated with Figure 5.

Specific Comments

Pages are not numbered.

The following sentence is run-on and lacks grammatical sense:"We found the pathogenic AID R190X mutant (AIDR190X) without the C terminal 9 amino acids (aa) failed to support efficient CSR in AID-deficient primary B cells stimulated by LPS and IL4 (termed as CSR-activated B cells), in which assay only a tiny portion (~3%) of primary B cells switched to IgG1 (Fig. 1A), consistent with previous reports (Barreto et al, 2003; Geisberger et al, 2009; Ta et al, 2003; Zahn et al, 2014). What does "in which assay only a tiny portion...." mean?

Fig. 1A and S1A - representative flow cytometry must be shown for all experiments
Figure 1B - A better description of the deamination Assay used in this figure is required.
Figure 1C -"Blue, junctions orientated from left to right; Red, junctions orientated from right to left." There are no "blue" or "red" junctions evident
Figure 1D,E: The position of the AGCT/RGYW (red) are very difficult to see.

Suppl. fig. 1C. The authors write that AID_R190X accumulates to higher levels than WT AID in nuclei. Although this seems to occur in primary AID KO B cells, this seems not to be the case for the CH12 complementation experiments.

The HTGTS assay is not defined or explained.

The authors write: "In the B cell complemented with wild-type AID, the junctions at the 5'S bait side frequently located in AID-preferred AGCT and other RGYW (R: A/G; Y: T/C; W: A/T) motifs (Fig. 1D)." However, this is not obvious from the figure 1D where only one peak coincides with the AGCT/RGYW position.

The authors write: "Collectively, the catalytic-active AID C mutants have low activity on the physiological IgH substrates in vivo and generate fewer breaks though with robust deamination activity. How the fully active AID C protein loses its deamination activity on the endogenous IgH substrate in vivo is a long-sought question." It is not clear why the author believe that

deamination activity of the AIDdeltaC mutant is lost in vivo. It is possible that deamination activity is intact and BER proteins are not recruited to the site thereby reducing DSB formation.

Fig. 1F : Is CRISPR-guided AID actually referring to AIDdeltaC? Why does the CRISPR guided WT AID not work in this system?

Fig. 1F and S1F - there is no clear explanation of the in vitro deamination assay anywhere in the text or legends.

Why use the APOBEC3G derived N terminus in the MBP-purified AID_cry deamination experiments? No explanation is given.

Suppl. fig. 2B. The authors write that the MCP-fusion AIDs are expressed to similar levels. Although the Western shows equivalent levels of fused AID proteins the MCP loading controls are widely different. I conclude that MCP-AID_cry and MCP-AID_R190X are overexpressed relative to the control.

What is PEM-seq?

Figure 2B,C. The author report that the mutation frequency is decreased in the miniS region for the AID_R190X as compared to WT (fig. 2B). However, inspection of the CRISPR-guided AID mutation frequency in the figure 2C suggests that opposite is true; the mutation frequency is lower for the WT as compared to AID_R190X. This goes back to the question of expression levels of these AID constructs. Thus, the conclusion drawn by the authors is not yet substantiated.

Suppl. fig. 4A. The authors report a qRT-PCR experiment but no where is the method they used described.

Figure 3B. What method was used to measure fluorescence in the nucleus?

Figure 4A,B,C: The author examine only MBP-mCherry-AID_cry and not the MBP-GFP-AID_cry+CTT. These sections should be re-positioned into the Suppl. figures as they do not have an control for comparison. Panel E of Figure 4 is the only relevant information.

Figure 5. The authors write: "Conversely, wild-type AID could not drive the formation of optoDroplet either in the cytoplasm or in the nucleus (Fig. 5A and 5B) despite at a comparable protein level to AID C in nuclei after CRM1i treatment in HEK293T cells (Fig. 5C)." How do we know that there is a comparable level of AID deltaC after CRM1i treatment? And - comparable to what? Finally, how does fig. 5C show this? Figure 5F. The color coding is impossible to discern.

The authors wrote: "To dissect key residues involved in AID C phase separation, we generated a panel of AID mutants with N-(N), C-terminus(C) deletions, and performed optoDroplet analysis (Fig. 5E and S6E)..... " this section of the Results and Figure 5E-G is extremely difficult to evaluate. The data are not well described and the Figure legends are no help.

The authors wrote: "In the control and AID samples, most of the AID protein was found in the cytoplasm with a diffused distribution pattern (Fig. 6A). However, a bright wild-type AID spot occurred inside of nuclear in the AIDC - transfected cells (Fig. 6A), which colocalized with nucleolus marked by FBL (Fig. 6A). These data suggest that AIDC can alter the subcellular location of endogenous wild-type AID protein."

A major concern regarding the studies shown in Figure 7 is that the levels of ectopic expression of AID mutants was not directly analyzed and compared to WT AID by Western. The authors note that "In the AID C/AID heterogeneous cells, the estimated nuclear molecular ratio was around 10:1 based on measurements of nuclear protein levels in AID C or AID expressing B cells (Fig. 3A, Fig. 6B and Fig. S4A)", thus highlighting these concerns. It is unclear how the authors determine the protein ratios that they cite.

#EMBOJ-2021-109324

Response to Reviews:

We have performed new sets of experiments to address the concerns from the referees and also revised the manuscript as indicated below in our point-by-point response.

Referee #1:

AID incudes DNA lesions and plays an important role in class switch recombination and somatic hypermutation during B cell development. Xie and colleagues discovered that a C-terminal deletion mutant of AID shows abnormal nucleus localization, accompanied by a loss of function in triggering class switch recombination. The authors proposed that the mutant has increased condensation properties, which, in a heterozygous background, traps the wild-type protein from approaching the target sites. This study highlighted the functional importance of intranuclear localization of AID and resolved a puzzling observation that the C-terminal deletion mutant has increased nuclear localization but decreased activity. The data quality is generally high. However, data supporting the trapping model need to be strengthened. In addition, the mechanism of how AID mutants are concentrated in nucleoli has not been elucidated.

Response: We thank this referee for the commendation on the significance and data quality of our work, and the critical and insightful suggestions. In the revision, we have performed several new sets of experiments to address the specific comments point-by-point as follows. We believe the proposed co-condensation model is further strengthened by the new data.

Major points:

1. To prove the model that AID R190X traps AID WT in condensates and prevents WT approaching target sites, it is critical to quantify the nuclear fluoresce intensity of WT AID in the presence and absence of AID R190X. In Fig. 6A and 6B, it looks like in AID R190X cells, AID WT has a similar nuclear intensity (outside the condensates region), if not higher, than the one without R190X. If this is true by quantification, it will challenge the trapping model. An explanation or alternative mechanism will be needed.

Response: We have performed a new set of experiments in the early-G1 B cells to address this comment.

As suggested by the observations from the Nussenzweig Lab (Wang *et al*, 2017), the majority of nuclear-localized AID occurs at the early-G1 phase. In this context, in the cycling B cells, the nuclear full-length AID cannot be directly compared in the presence/absence of AID^{R190X}, as the former may represent an accumulation of trapped nuclear full-length AID in all cell cycle phases. In the early-G1 B cells obtained with the same procedure as described in Wang et al., we observed a higher fraction of nuclear AID than that in the cycling B cells (Fig. R1A, also as Revised Fig. S6C). Thus, to properly compare the nuclear AID levels (outside condensates), we performed new experiments and calculated the relative signals in early-G1 cells (Fig. R1B and R1C, also as Revised Fig. 3B and 3C). We observed that AID-GFP had a significantly reduced nuclear intensity outside the co-condensation region with AID^{R190X} (Fig. R1C, also as Revised Fig. 3C), further supporting the co-condensation model.

We thank this referee for this insightful suggestion, which, we believe, strengthens our model.

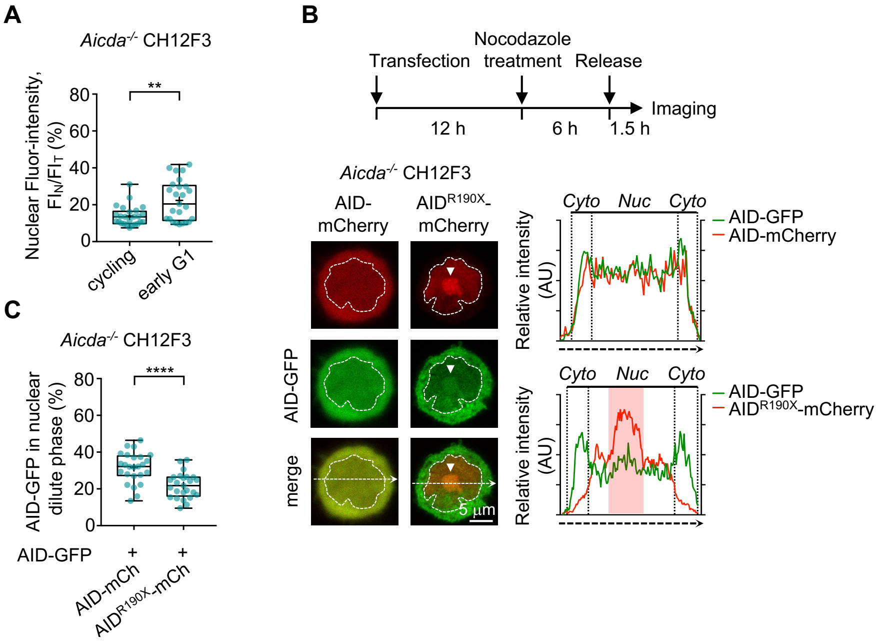

Fig. 1 to referee: Co-condensation of wild-type AID with AID$^{\Delta C}$ in B cell nucleus. (**A**, also presented as Revised **Fig. S6C**) The fluorescence intensity of ectopically expressed AID-GFP in each *Aicda*$^{-/-}$CH12F3 cell nucleus was measured and normalized to the fluorescence intensity of the whole cell. The normalized fluorescence intensity in cycling cells and early G1 cells upon nocodazole release is shown by box plots, respectively. FI_N: fluorescence intensity in the nucleus; FI_T: fluorescence intensity in the whole cell. "+" indicates the mean level. Each dot indicates one cell. Unpaired two-tailed Student's *t*-test was performed. **, p<0.01. (**B**, also presented as Revised **Fig. 3B**) Co-condensation of AID and AID^{R190X} in the nucleus of CH12F3 cells in the early G1 phase. Top: experimental layout. GFP-tagged wild-type AID (green) and mCherry-tagged AID variants (red) were co-nucleofected in *Aicda*$^{-/-}$ CH12F3 cells. The resulting cells were arrested with nocodazole for 12 hours after transfection. Six hours later, nocodazole was removed from the system for 1.5 hours before imaging. Bottom left: representative images showing subcellular localization of indicated AID variants. The white dashed line depicts the shape of the nucleus based on Hoechst staining. The white arrow indicates the location of the co-condensed puncta. Bottom right: plot profiles of the images. Cyto: cytoplasm, Nuc: nucleus. (**C**, also presented as Revised **Fig. 3C**) Quantification of the relative fluorescence intensity of AID-GFP in diluted phase of cells is shown in (**B**). The fluorescence intensity of AID-GFP in diluted nuclear phase was normalized by the total intensity from the whole cell. Data are shown as box plots. "+" indicates the mean level. Each dot indicates one cell, and a total of 25 cells was counted. Unpaired two-tailed Student's *t*-test was performed. ****, p<0.0001.

2. Elegant optogenetic tools were implemented to show AID Delta C has a higher tendency to form condensates than AID WT in the nucleus. This is very nice. However, there are some technical caveats on the in vitro biochemistry showing AID condensation. The current in vitro data did not strengthen the conclusion. For reasons elaborated below, I would suggest either improving the in vitro condensation assays or removing this part and acknowledging the difficulty in obtaining high quantity of AID WT and R190X.
1) AID-Cry, but not AID WT and AID R190X, was used in the in vitro condensation assays. What is the physiological or pathological relevance of AID-Cry? 2) All the in vitro condensation assays included 10% PEG in the buffer, which is problematic because PEG can promote non-

specific condensation as a crowding reagent. It is better to test condensation without PEG. 3) MBP increases the solubility of protein, which also inhibits condensation in general. Using an MBP-tagged protein in the condensation assay would dramatically affect the protein-of-interest's ability to form condensates.

Response: We are happy that the referee appreciates the optogenetic data and thank the referee for the critical comment on the *in vitro* biochemistry data. In the revision, we performed new biochemistry experiments to address the specific comments and re-organized the manuscript to move the majority of this part to the supplementary figures.

1). We acknowledge the difficulty of obtaining a high quantity of AID and R190X proteins in the revised manuscript (Page 12, Line 4-12). In brief, although we are able to purify MBP-AID and MBP-AID^{R190X} proteins from Expi293F cells for the deamination assay (Revised Fig. S1E and S1F), the purity and quantity do not allow the application in the condensation assays. We use chimeric protein AID^{cry} as a surrogate of $AID^{\Delta C}$ for the following reasons: AID^{cry} behaves similarly as AID^{R190X} in CSR (Revised Fig. S1A), guided-AID (Revised Fig. 1A, 1B and 1D), dominant-negative effect (Revised Fig. S8E), optoDroplet (Revised Fig. 2B and 2C) and 'trapping' (Revised Fig. 3A and S6E) assays. However, as suggested by this referee, we have moved most of the biochemistry results into the supplementary figures. In the revised main figures, we present a comparison of AID^{cry} and $AID^{cry+CTT}$ to briefly discuss the role of the C terminal tail (CTT), as the CTT greatly diminishes its condensation ability.

2). We show additional experiments of characterizing $AID^{\Delta C}$ protein condensation with/without the crowding agent in the Revised Supplementary figures (Revised Fig. S9D). Similar reagents were used in protein condensation assay (e.g. Pol II CTD with dextran by the Darzacq and Zweckstetter labs; tau with 10% PEG by the Wolozin lab; Brd4/Med1 IDRs with 10% PEG8000 by the Young lab, etc). To keep the data in higher quality, we only present the results with proper controls, e.g. the comparison of AID^{cry} and $AID^{cry+CTT}$ under the same condition. As this referee pointed out, the optoDroplet assay and other *in vivo* assays have already demonstrated the higher tendency of $AID^{\Delta C}$ condensation. Based on the observation from *in vivo* and *in vitro* experiments, we think that AID^{R190X} protein unlikely condensation by itself (as discussed below). In this context, the crowding agent might function as the unknown *in vivo* factor driving AID^{R190X} condensation, which could mimic the *in vivo* condensation.

3). To exclude the impact of the MBP-GFP tag on condensation, we purified MBP-AID^{cry} protein and used PreScission protease to cut the MBP-tag off. The AID^{cry} protein underwent phase separation under the same experimental condition *in vitro* (Revised Fig. S9E and S9F). However, the removal of MBP-tag frequently caused the further degradation of AID^{cry}, prohibiting its further characterization.

In sum, we moved most of the biochemistry data into supplementary as this referee suggested, and we carefully described the data in the revised manuscript.

3. The physical properties of R190X condensates formed in cells needs to be better characterized (Figure 3A, 5A and 6A): 1) FRAP performed both in condensates and the surrounding nuclear compartment to determine if condensation reduces R190X mobility; 2) Quantifying the fold-enrichment of R190X in condensates as compared to the surrounding area.

Response: We thank the referee for these suggestions. In the revision, we performed the suggested experiments.

1). We performed FRAP experiments in both condensates and the surrounding nuclear region to further characterize the properties of AID^{R190X} condensates in B cells. After photobleaching, fluorescence signals in both puncta and the surrounding region recovered with similar kinetics (Fig. R2A, only shown in the rebuttal letter). Along the same line, we also performed FRAP in the optoDroplet assay in B cells and reached the same conclusion (Fig. R2B, only shown in the rebuttal letter). The results reveal a liquid-like nature of the condensates.

2). We quantified the fold-enrichment of AID^{R190X} in condensates. By measuring the fluorescence intensity, we found that the AID^{R190X} protein is enriched at about two-fold in the nuclear condensates (Fig. R2C, only shown in the rebuttal letter).

We have incorporated these further characterizations into the revised manuscript as abovementioned.

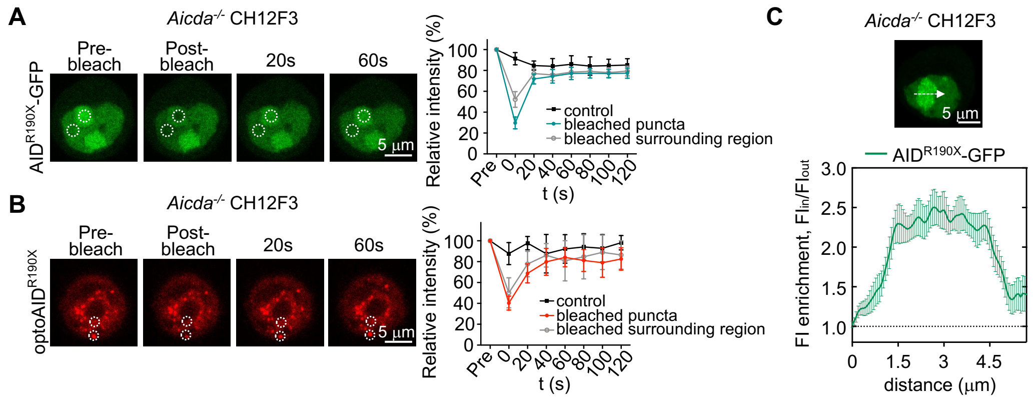

Fig. 2 to referee: Characterization of nuclear AID^{R190X} in condensates. (**A**) (Left) Representative images of FRAP experiment in *Aicda*$^{-/-}$ CH12F3 cells with AID^{R190X}-GFP expression. Dashed line circles indicate the photobleaching sites. (Right) Intensity recovery lines in FRAP experiment. Bleaching occurs at t = 0 s. The background-subtracted fluorescence intensities are normalized by pre-bleach values. Mean with SD of 8 pairs of AID^{R190X} condensates and surrounding regions are shown. (**B**) (Left) Representative FRAP images of pre-assembled optoAID^{R190X} droplets and the surrounding regions in *Aicda*$^{-/-}$ CH12F3 cells. Dashed line circles bleached dots. (Right) Intensity recovery lines in FRAP experiment, same as (A). Mean with SD of 7 pairs of optoAID^{R190X} droplets and surrounding regions are showed. (**C**) Quantification of the relative enrichment of AID^{R190X}-GFP inside the puncta. Top: a representative image indicates the measurement of fluorescence intensity (FI) along the white line. Bottom: enriched fluorescence intensity (FI) along AID^{R190X}-GFP puncta. Mean with standard error of the mean (SEM) are shown (n=6). FI_{in}: fluorescence intensity inside the puncta; FI_{out}: fluorescence intensity outside the puncta.

4. AID R190X condensates form on nucleoli or other nuclear compartments (Fig 3A and S4B). This strongly suggests AID 190X does not form condensates by itself. Instead, it is recruited to other pre-formed nuclear condensates. This mechanism needs to be clearly acknowledged and the candidate proteins in nucleoli that recruit AID mutants should be discussed.

Response: We agree with the referee that AID^{R190X} is unlikely to undergo condensation by itself. The missing link between the AID^{R190X} and its condensation driver could be a protein or

RNA/DNA, as we showed that both the surface positive-charged patch and the N-terminus are required for the observed AID^{R190X} condensation (Revised Fig. 4A, 4B and S7A). Thus, we have discussed the potential cofactors in the revised manuscript (Page 14, second paragraph). The potential cofactors include CTNNBL1 (Conticello *et al*, 2008), hnRNPs (Hu *et al*, 2015), other RNA-binding proteins (Chen *et al*, 2018; Nowak *et al*, 2011), or a previously unknown AID cofactor. Besides the speculated protein cofactor, nascent or other non-coding RNA could be another candidate. To identify and validate such a factor will require a significant amount of work, which would be beyond the scope of the current manuscript. Thus, based on this referee's suggestion, we discussed the potential factors in the revision.

Minor points

1. The experiments of testing the chimeric protein AID R190X-AID E58Q is elegantly designed. Is this chimeric protein enriched in specific nuclear localization?

Response: We have performed the suggested experiment. When the GFP-tagged AID^{R190X}-AID^{E58Q} was expressed in *Aicda*$^{-/-}$primary B cell, the chimeric protein functions similarly as the full-length AID (Fig. R3, only shown in the rebuttal letter). We did not observe relative enrichment of this chimeric protein in the nuclear compartment, which is consistent with the result of the CSR assay (Revised Fig 1C).

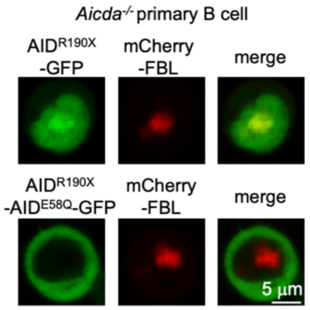

Fig. 3 to referee: Chimeric dimer protein AID^{R190X}-AID^{E58Q} does not enrich in the nuclear compartment. Representative images showing the subcellular location of indicated AID variants in *Aicda*$^{-/-}$ primary B cells. The nucleolus is indicated by mCherry-fibrillarin (FBL).

2. Can the author comment on how the truncated C-terminus in AID R190X helps to recruit AID to the target sites?

Response: We interpret this referee's question as to how the AID C-terminus helps AID targeting. First of all, we must acknowledge that the *IgH* targeting of AID is regulated through a multilayer process involving topological domain, super-enhancer, transcription, non-coding RNAs, etc. Here, the truncated AID C-terminus plays a negative role in this pathogenic setting, as the C-terminus can inhibit the pathogenic condensation and full-length AID is properly recruited. Furthermore, to establish a causal link between condensation and defective CSR, we performed new experiments with the synthetic AID-FUS fusion protein. The synthetic AID-FUS protein (prone to condensation in B cells) supported CSR at a lesser extent compared to an AID-FUSm protein (does not form puncta in B cells).

3. The authors proposed that the frequent infiltration in nuclear compartment of AID delta C might serve as a way to avoid aberrant AID damages. This is a very intriguing point. Is it

possible to design experiments to test this idea, e.g., by using the mutants that have attenuated condensation properties?

Response: We have drawn the conclusion based on the clinical observations of the $AID^{\Delta C}$ immunodeficient patients. As discussed in the revised manuscript (Page 11, second paragraph), the mutants with attenuated condensation properties also have altered AID deamination activity (Abdouni *et al*, 2018). So far, a separation-of-function mutation cannot be identified, which is not surprising as AID is a relatively small protein (198 aa) and the critical aa residues may adapt to multiple functions in different settings. However, since we do not have direct experiments performed for the aberrant damages, we have tuned down the statement, including further editing in the abstract and main text.

Referee #2:

Xie et al.
C-terminal deletion-induced condensation sequesters AID from IgH targets in immunodeficiency

In humans, the cytidine deaminase AID drives somatic diversification of antibody genes during the secondary immune response. It targets the V regions of the Ig loci causing hypermutation, allowing affinity maturation of antibody specificity, and it also causes class switch recombination in which the constant region is switch from mu (IgM) to one of the downstream isotypes. Loss of AID activity leads to immunodeficiency characterised by poor affinity maturation and high levels of unswitched IgM antibody. Most mutations in AID affect its ability to deaminate C effectively and are recessive. However, a rare class of mutations results in loss of the C-terminus of the protein and behave in a dominant manner.
The reason for this has been unclear although a number of theories have been advanced, related to proposed functions of the C terminus of AID.

The current manuscript provides a plausible explanation for why loss of the AID C-terminus acts as a dominant negative mutation by proposing that in the absence of the C-terminus the protein form condensates and can be sequestered into membraneless compartments within the nucleus, and indeed can bring wild type AID with it, reducing the activity of the enzyme at the Ig loci.

The experiments to support this hypothesis are well performed, well controlled and clearly presented. The paper uses a wealth of up-to-date approaches I have only some minor comments and questions about the data that is presented. However, I am less convinced about the extent of the advance and its general interest, rather than interest to those in the field, in terms of understanding the role of the C terminus of AID in the function of the protein. The work nicely explains why these rare C-terminus mutants act as dominant negatives, but it does not address whether phase separation is a part of the normal biology and/or regulation of AID nor how the C terminus prevents inappropriate sequestration of the protein in existing membraneless organelles. Further insight into these points could significantly raise the significance of the study.

Response: We thank this referee for the appreciation of AID dominant-negative mutation elucidation, and also appreciate the comment on wild-type AID regulation. The dominant-negative effect of these rare C-terminus mutants was a mysterious question in the field and many models have been proposed, including interaction with a cofactor(s) beyond the NES function (the Neuberger, Honjo Labs), and the link between DNA breakage and recombination (the Honjo, Stavnezer and Di Noia Labs). We do agree with this referee that the role of protein condensation in normal AID activity regulation is an important question for future study, however the property of pathological protein is also of great interest. In this context, we have discussed several implications in the discussion section.

On the other hand, we think the AID dominant-negative case could be an example to explain other dominant-negative effects in genetic disorders. We have performed preliminary characterization of several dominant-negative mutants in immune disorders. For example, we found a dominant-negative mutant of STAT3, R382Q, also showed ‘trapping’ phenotypes in the optoDroplet assay (Fig. R4, only shown in the rebuttal letter). We are hesitant to include the data in this manuscript, as they are still preliminary and not directly relevant to the focus of the paper. However, the explanation of the dominant-negative effect has the potential to be applied to a wider field.

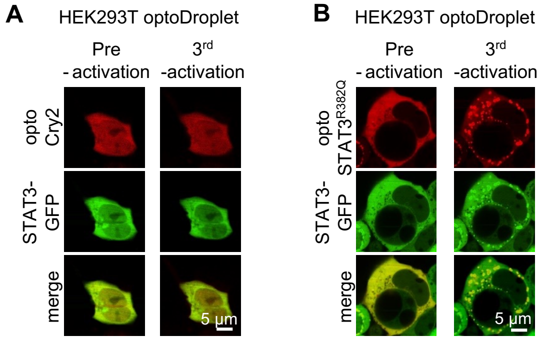

Fig. 4 to referee: Co-condensation as a working model for dominant-negative mutant of STAT3. STAT3-GFP fusion protein (green) was co-expressed with the optoDroplet system (red), and representative images before activation and after triple blue light activation are shown. STAT3-GFP cannot form puncta when co-expressed with optoCry2 (red) (**A**), but can be trapped into the optoSTAT3^{R382Q} condensates (**B**) in HEK293T cells.

Specific points & questions:

Does the MBP tag affect the phase separation behaviour of AID in the in vitro assays shown in Figure 4?

Response: We have performed the *in vitro* condensation experiment without the MBP tag. In the new experiment, we purified MBP-AID^{cry} protein and used PreScission protease to cut the MBP-tag off. The AID^{cry} without the MBP-tag underwent phase transition in the same experimental condition *in vitro* (Revised Fig. S9E and S9F). However, the 'tag-off' protein tends to undergo degradation *in vitro*. Thus, for the biochemistry assays, we have included MBP-GFP as controls (Revised Fig. S9C).

Do the mutations (shown expressed in CH12 in Fig 5G) that reduce droplet formation by the deltaC mutant also rescue any of the lost CSR-inducing activity in CH12?

Response: In the revision, we performed additional experiments to characterize these mutants with attenuated condensation ability on top of AID^{R190X}, which rescued the dominant-negative effect in both CH12F3 cells and CSR-activated B cells. However, the mutant, AID$^{R190X\Delta N12}$ or AID$^{R190X\text{-}4R}$, failed to support CSR by itself (Revised Fig. S8B), as the N-terminus and the 4R patch are required for AID activity (Abdouni *et al.*, 2018). So far, a separation-of-function mutation cannot be identified, which is not surprising as AID is a relatively small protein (198 aa) and the critical aa residues may adapt to multiple functions in different settings. For example, in the revision, we performed an Ala-screening experiment on top of AID^{R190X} by constructing >150 point-mutations, but did not find such a CSR-rescue mutation.

There are a few places where the English needs some attention and a few typos need correction (page numbers would have been helpful):

Abstract, line 5: deficiency should be deficient

Section heading: AIDdeltaC shows a defect in targeting ... add 'a'

Same section first line: not sure what 'The defect of the arousing DNA repair ...' means.

Section AIDdeltaC is frequently enriched ... line 2: experimental

Response: We thank this referee for the careful reading of the manuscript. We have rewritten some of the sections and corrected the grammar.

Referee #3:

In this manuscript Xie and coworkers have explored the dominant negative phenotype of AID truncated at the C-terminus. This is a worthy effort since the molecular mechanism of why truncated AID (t-AID) fails to support CSR has been an open question for many years. The authors have applied a long series of assays to unravel this question. They have confirmed the work of others shows that t-AID supports deamination in vitro but not CSR in vivo. They have engineered a CRISPR model to drag t-AID to its template and show that it can mediate CSR. They confirm the work of others showing that t-AID diffuses from the cytoplasm to the nucleus. In new studies they demonstrate that when t-AID is in the nucleus it can form molecular condensates. They go on to explore the nature of the AID condensates and suggest that the presence of t-AID can alter the localization of full length AID and move it into the nucleolus and can force AID to also form condensates. They wish to conclude that the dominant negative phenotype of AID is directly correlated to the formation of condensates in the nucleus. This manuscript reports many studies and much data. Unfortunately, the presentation of the data is very frequently unclear and the writing is replete with grammatical and syntactical mistakes. More importantly the manuscript is very poorly edited and spends space on confirming the results of others and not enough effort or focus on clearly explaining their new studies and the assays they perform. Although there may be some important and novel findings it is difficult to assent to the authors conclusions as in many instances that data are unclear or lacking.

A partial set of examples of how the manuscript could be streamlined follow: Much of Figure 1A-E and Suppl. figure 1 show that previously described AID mutants fail to support CSR but have deamination activity. Not much novel information is provided and should be moved to Suppl. Figures. The new studies begin with Fig. 1F-H and go together with Figure 2 A-C. Figure 3A, B provide little new information and should be moved to Suppl. Figures. The experiment shown in Figure 3C is relevant to the new studies. Figure 4A-D is weak because there is no WT control and therefore should be moved to Suppl. Figures. Figure 4 E is new and interesting information and could be amalgamated with Figure 5.

Response: We thank this referee for the kind suggestion on the re-organization of the manuscript. In the revision, we have streamlined the manuscript as suggested. The Revised Figure 1 only presents the novel discoveries (previous Fig. 1F-H and Fig. 2A-C). The Revised Figure 2 now presents the live imaging data (previous Fig. 3C), optoDroplet data (previous Fig. 5), and a new set of data. The majority of the biochemistry data were relocated to supplementary figures, and the previous Fig. 4E was presented in the Revised Figure 5. We hope that the referee will find the manuscript much improved.

Specific Comments

Pages are not numbered.

Response: We added the page number and line number for the main text.

The following sentence is run-on and lacks grammatical sense:"We found the pathogenic AID R190X mutant (AIDR190X) without the C terminal 9 amino acids (aa) failed to support efficient CSR in AID-deficient primary B cells stimulated by LPS and IL4 (termed as CSR-activated B cells), in which assay only a tiny portion (~3%) of primary B cells switched to IgG1 (Fig. 1A),

consistent with previous reports (Barreto et al, 2003; Geisberger et al, 2009; Ta et al, 2003; Zahn et al, 2014). What does "in which assay only a tiny portion...." mean?

Response: We have re-organized the manuscript according to this referee's suggestion, and this sentence was rewritten as "Pathogenic mutant AID^{R190X} with truncation of the C terminal 9 amino acids (aa) failed to support efficient CSR in CSR-activated B cells (Fig. S1A) and CH12F3 B cell line (Fig. S1B), consistent with previous reports (Barreto *et al*, 2003; Geisberger *et al*, 2009; Ta *et al*, 2003; Zahn *et al*, 2014)". We have also checked the revised text for grammar.

Fig. 1A and S1A - representative flow cytometry must be shown for all experiments.

Response: The representative flow cytometry plots are now shown in Fig. S1A and S1B. Similar problems have been fixed for the other experiments as in Revised Fig. 2G, 4C, 4D, 6B, and S3A.

Figure 1B - A better description of the deamination assay used in this figure is required.

Response: A schematic illustration of *in vitro* deamination assay is shown in Revised Fig. S1F, with more information in the corresponding figure legend. We also further edited the method section for this assay (Page 17).

Figure 1C -"Blue, junctions orientated from left to right; Red, junctions orientated from right to left." There are no "blue" or "red" junctions evident.

Response: The Panel is now in Revised Fig. S2A. We corrected the misleading description in the figure legend. Blue/red indicates the peak.

Figure 1D, E: The position of the AGCT/RGYW (red) are very difficult to see.

Response: The Panel is now in Revised Fig. S2B. We have enlarged the illustration on the top to indicate the positions.

Suppl. fig. 1C. The authors write that AID_R190X accumulates to higher levels than WT AID in nuclei. Although this seems to occur in primary AID KO B cells, this seems not to be the case for the CH12 complementation experiments.

Response: We are sorry for the misleading presentation. The representative western blots in Revised Fig. S1C show the expression level of AID and AID^{R190X} with the total cell lysate samples. In Revised Fig. S1D, we analyzed the indicated subcellular fractions of AID and AID^{R190X} in *Aicda*$^{-/-}$ CH12F3 cells. Although the total AID^{R190X} is lower than WT AID in the whole-cell lysates (Revised Fig. S1C), AID^{R190X} accumulated at a higher level than WT AID in the nucleus (Revised Fig. S1D). It is also consistent with the fluorescence analysis for the subcellular localization of AID and AID^{R190X} in Revised Fig. S4B.

The HTGTS assay is not defined or explained.

Response: We have further clarified the HTGTS assay in the revision (Page 5, first paragraph). And the method description has been further edited.

The authors write: "In the B cell complemented with wild-type AID, the junctions at the 5'Sμ bait side frequently located in AID-preferred AGCT and other RGYW (R: A/G; Y: T/C; W: A/T) motifs (Fig. 1D)." However, this is not obvious from the figure 1D where only one peak coincides with the AGCT/RGYW position.

Response: In the revised figure (Revised Fig. S2B), we have enlarged the schematic illustration of the AGCT/RGYW position. In addition, we also indicated the 7 coincident peaks with asterisks.

The authors write: "Collectively, the catalytic-active AIDΔC mutants have low activity on the physiological IgH substrates in vivo and generate fewer breaks though with robust deamination activity. How the fully active AIDΔC protein loses its deamination activity on the endogenous IgH substrate in vivo is a long-sought question." It is not clear why the author believe that deamination activity of the AIDdeltaC mutant is lost in vivo. It is possible that deamination activity is intact and BER proteins are not recruited to the site thereby reducing DSB formation.

Response: Thanks for pointing out this inaccurate description. The sentence has been changed to "Therefore, the catalytic-active $AID^{\Delta C}$ protein induces fewer DNA breaks at *IgH* locus" to precisely describe the result.

Fig. 1F: Is CRISPR-guided AID actually referring to AIDdeltaC? Why does the CRISPR guided WT AID not work in this system?

Response: The term "CRISPR-guided AID" refers to the approach of synthetic recruitment of AID with CRISPR/Cas9. When the WT AID was introduced into the system, it could generate mutations, but at a much lower frequency compared to the $AID^{\Delta C}$. CRISPR-guided WT AID could induce an increased switch to IgA compared to cells with MCP control (Revised Fig. 1A). The higher CSR levels of $MCP\text{-}AID^{\Delta C}$ compared to MCP-AID could partially be attributed to the active nuclear export of MCP-AID protein.

Fig. 1F and S1F - there is no clear explanation of the in vitro deamination assay anywhere in the text or legends.

Response: We drew a schematic illustration of *in vitro* deamination assay and presented a representative gel image (Revised Fig. S1F) to further explain the experiment.

Why use the APOBEC3G derived N terminus in the MBP-purified AID_cry deamination experiments? No explanation is given.

Response: We have now re-organized the text and data as described on Page 12. In brief, although we are able to purify MBP-AID and $MBP\text{-}AID^{R190X}$ proteins from Expi293F cells for the deamination assay (Revised Fig. S1E and S1F), the purity and quantity do not allow the

application in the condensation assays. The difficulty in protein purification is well-documented in the field (as described on Page 12, Line 4-12). The chimeric protein AID^{cry} was used in protein biochemistry and structure studies (Qiao *et al*, 2017). We use the chimeric protein AID^{cry} as a surrogate of $AID^{\Delta C}$ in this study for the following reasons: AID^{cry} behaves similarly to AID^{R190X} in CSR (Revised Fig. S1A), guided-AID (Revised Fig. 1A, 1B and 1D), dominant-negative effect (Revised Fig. S8E), optoDroplet (Revised Fig. 2B and 2C) and 'trapping' (Revised Fig. 3A and S6E) assays.

Suppl. fig. 2B. The authors write that the MCP-fusion AIDs are expressed to similar levels. Although the Western shows equivalent levels of fused AID proteins the MCP loading controls are widely different. I conclude that MCP-AID_cry and MCP-AID_R190X are overexpressed relative to the control.

Response: We are sorry for the misleading labeling. The upper "MCP" panel indicates the MCP-AID variant expression levels, and the lower "Flag" panel indicates the level of Flag-Cas9 used as a loading control. MCP-AID expressed at a higher level than that of MCP-$AID^{\Delta C}$ (Revised Fig. S3B), which is reminiscent of the AID and $AID^{\Delta C}$ expression levels in B cells (Revised Fig. 1C). We have edited the text on Page 6.

What is PEM-seq?

Response: PEM-seq (primer-extension-mediated sequencing) (Yin *et al*, 2019) is an updated version of HTGTS method. PEM-seq combines HTGTS with targeted sequencing and thus could quantitatively detect end-joining events initiated by CRISPR/Cas9 tools through translocation capture. It can also be used for characterizing off-target sites and other abnormal chromosomal structures, including genome-wide translocations, small indels, and large deletions generated by CRISPR/Cas9. We have further edited the corresponding text on Page 6, and made additional changes in the method section. All the details of PEM-seq can be found in our recent publication Liu et al., 2022 (https://doi.org/10.1016/ j.xpro.2021.101088).

Figure 2B, C. The author report that the mutation frequency is decreased in the miniS region for the AID_R190X as compared to WT (fig. 2B). However, inspection of the CRISPR-guided AID mutation frequency in the figure 2C suggests that opposite is true; the mutation frequency is lower for the WT as compared to AID_R190X. This goes back to the question of expression levels of these AID constructs. Thus, the conclusion drawn by the authors is not yet substantiated.

Response: We are sorry for the misleading. (1). As abovementioned, the expression of MCP-AID constructs is consistent with AID variants. The misleading came from the unclear labelling of the panel. (2). The referee actually pointed out the results, which we aimed to present. In Revised Fig. 1E (previous Fig. 2B), WT AID induced a substantial level of mutations at the mini-S region, while AID^{R190X} induced mutations at a much lower frequency. In Revised Fig. 1F (previous Fig. 2C), the mutation frequency of MCP-AID and MCP-$AID^{\Delta C}$ is the opposite, because in the latter, the AID variant is recruited through a different approach. From the panels, the mutation profiles are different: in the latter, mutations happen around the sgRNA-targeting

sites (marked by blue bars at the bottom). The misleading may also come from our previous ‘crowd’ presentation with many confirmatory data. We hope the referee will find the revised version much easier to follow.

Suppl. fig. 4A. The authors report a qRT-PCR experiment but no where is the method they used described.

Response: The detailed description of the qRT-PCR (Revised Fig. S4A) method is now available in the Materials and Methods section, which is subtitled as “Assessment of AICDA expression level with qPCR” (Page 23). In the revision, we have added more details.

Figure 3B. What method was used to measure fluorescence in the nucleus?

Response: We have added more details in the method section. In brief, the ImageJ software package (1.53) was used for quantitative measurement of the fluorescence in the cells. The region of the nucleus was depicted based on Hoechst staining. The fluorescence intensities in the nucleus and the whole-cell were measured for each cell, and the background intensity was further subtracted. The relative nuclear intensity was calculated by normalizing to the whole-cell intensity.

Figure 4A,B,C: The author examine only MBP-mCherry-AID_cry and not the MBP-GFP-AID_cry+CTT. These sections should be re-positioned into the Suppl. figures as they do not have an control for comparison. Panel E of Figure 4 is the only relevant information.

Response: We thank the referee for this kind suggestion, and we have re-organized the figures in the revised manuscript as suggested.

Figure 5. The authors write: "Conversely, wild-type AID could not drive the formation of optoDroplet either in the cytoplasm or in the nucleus (Fig. 5A and 5B) despite at a comparable protein level to AIDΔC in nuclei after CRM1i treatment in HEK293T cells (Fig. 5C)." How do we know that there is a comparable level of AID deltaC after CRM1i treatment? And - comparable to what? Finally, how does fig. 5C show this? Figure 5F. The color coding is impossible to discern.

Response: The previous Figures 5A, 5B, 5C, and 5F, are now presented as Revised Figure 2B, 2C, 2D, and 4B. To clarify the aim of the experiment, we rewrote the sentence as “Conversely, wild-type AID could not drive the formation of optoDroplet either in the cytoplasm or in the nucleus (Fig. 2B and 2C), despite the nuclear protein expression level of wild-type AID in the presence of CRM1i being comparable to that of $AID^{\Delta C}$ in HEK293T cells (Fig. 2D)”. Here, we want to exclude the possibility that the nuclear location or nuclear AID protein level affects the interpretation of the data. For Revised Figure 4B, we have changed the order of the legends to better illustrate the data, as some of the lines are highly overlapped.

The authors wrote: "To dissect key residues involved in AIDΔC phase separation, we generated a panel of AID mutants with N-(ΔN), C-terminus(ΔC) deletions, and performed optoDroplet analysis (Fig. 5E and S6E)..... " this section of the Results and Figure 5E-G is extremely difficult to evaluate. The data are not well described and the Figure legends are no help.

Response: The previous Fig. 5E and S6E are now presented as Revised Fig. 4A and S7A. The panels show summarized data from the optoDroplet images. In Revised Fig. S7A, we illustrated the architectures of the mutants with a focus on the mutated amino acid residues. In Revised Fig. 4A, we depicted the AID and its variants based on the protein structure (Qiao et al., 2017) and predicted structure by AlphaFold (Jumper et al, 2021). We further edited the text and figure legend to better present the results.

The authors wrote: "In the control and AID samples, most of the AID protein was found in the cytoplasm with a diffused distribution pattern (Fig. 6A). However, a bright wild-type AID spot occurred inside of nuclear in the AIDΔC - transfected cells (Fig. 6A), which colocalized with nucleolus marked by FBL (Fig. 6A). These data suggest that AIDΔC can alter the subcellular location of endogenous wild-type AID protein."

Response: We interpret this comment as an unclear explanation. In the revision, the previous Fig. 6A is incorporated into the Revised Fig. 3. In the new figure, we elaborate on the 'trapping' phenotype from three layers: (1) in Revised Fig. 3A, we show the 'trap' of endogenous AID by ectopically expressed AID^{R190X} in B cells; (2) in Revised Fig. 3B&C, we show results from a new set of experiments. In the early-G1 phase, the ectopically expressed AID and AID^{R190X} showed the 'trap' phenomenon; (3) in Revised Fig. 3D, we present the dynamic trapping with optoDroplet assay. In the accompanied Revised Fig. S6, we show additional two layers of evidence: (4) the 'trapping' can happen in non-B U2OS cells; (5) an irrelevant optoDroplet of FUS cannot trap AID. Collectively, we think these data strongly support the co-condensation model.

A major concern regarding the studies shown in Figure 7 is that the levels of ectopic expression of AID mutants was not directly analyzed and compared to WT AID by Western. The authors note that "In the AIDΔC/AID heterogeneous cells, the estimated nuclear molecular ratio was around 10:1 based on measurements of nuclear protein levels in AIDΔC or AID expressing B cells (Fig. 3A, Fig. 6B and Fig. S4A)", thus highlighting these concerns. It is unclear how the authors determine the protein ratios that they cite.

Response: We thank the referee for the comment. We have added new data and performed new analyses to address the concern.

(1) We added the western blot data as suggested in Revised Figures S10A and S8D. For the AID dimer experiment, AID-AID was expressed at a lower level compared to $AID^{\Delta C}$-AID, which is consistent with the CSR level. Nonetheless, the data suggest the $AID^{\Delta C}$-AID dimer is active *in vivo*.

(2) In the previous version, we estimated the molecular ratio by measuring the fluorescence signals in imaging experiments and calculating the qPCR reads. The estimated ratio is ~10:1. In the revision, we only show the measuring data based on the fluorescence signals in

the imaging experiment shown in Revised Fig. S4B. In total, we measured 20 cells and got an average relative ratio of 7.2 (Fig. R5, only shown in the rebuttal letter). Thus, we wrote as "the estimated nuclear molecular ratio was around 7:1" in the revised manuscript.

Overall, we thank this referee for the patient reading of our manuscript and the critical comments, which improved the revised manuscript a lot.

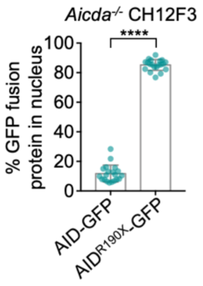

Fig. 5 to referee: Nuclear expression ratio of AID and AID^{R190X} in *Aicda*$^{-/-}$ CH12F3 cell. In nucleofected *Aicda*$^{-/-}$ CH12F3 cells, the fluorescence intensity in the nucleus was measured and normalized to that in the whole cell. The relative enrichment of GFP-tagged AID or GFP-tagged AID^{R190X} in the nucleus of each cell is shown in the bar plot (n=20 for AID-GFP and AID^{R190X}-GFP, respectively). Unpaired two-tailed Student's *t*-test was performed. ****, $p<0.0001$.

References:

Abdouni HS, King JJ, Ghorbani A, Fifield H, Berghuis L, Larijani M (2018) DNA/RNA hybrid substrates modulate the catalytic activity of purified AID. *Mol Immunol* 93: 94-106

Ash PEA, Lei S, Shattuck J, Boudeau S, Carlomagno Y, Medalla M, Mashimo BL, Socorro G, Al-Mohanna LFA, Jiang L *et al* (2021) TIA1 potentiates tau phase separation and promotes generation of toxic oligomeric tau. *Proc Natl Acad Sci U S A* 118

Barreto V, Reina-San-Martin B, Ramiro AR, McBride KM, Nussenzweig MC (2003) C-terminal deletion of AID uncouples class switch recombination from somatic hypermutation and gene conversion. *Mol Cell* 12: 501-508

Boehning M, Dugast-Darzacq C, Rankovic M, Hansen AS, Yu T, Marie-Nelly H, McSwiggen DT, Kokic G, Dailey GM, Cramer P *et al* (2018) RNA polymerase II clustering through carboxy-terminal domain phase separation. *Nat Struct Mol Biol* 25: 833-840

Chen J, Cai Z, Bai M, Yu X, Zhang C, Cao C, Hu X, Wang L, Su R, Wang D *et al* (2018) The RNA-binding protein ROD1/PTBP3 cotranscriptionally defines AID-loading sites to mediate antibody class switch in mammalian genomes. *Cell Res* 28: 981-995

Conticello SG, Ganesh K, Xue K, Lu M, Rada C, Neuberger MS (2008) Interaction between antibody-diversification enzyme AID and spliceosome-associated factor CTNNBL1. *Mol Cell* 31: 474-484

Geisberger R, Rada C, Neuberger MS (2009) The stability of AID and its function in class-switching are critically sensitive to the identity of its nuclear-export sequence. *Proc Natl Acad Sci U S A* 106: 6736-6741

Hu W, Begum NA, Mondal S, Stanlie A, Honjo T (2015) Identification of DNA cleavage- and recombination-specific hnRNP cofactors for activation-induced cytidine deaminase. *Proc Natl Acad Sci U S A* 112: 5791-5796

Jumper J, Evans R, Pritzel A, Green T, Figurnov M, Ronneberger O, Tunyasuvunakool K, Bates R, Zidek A, Potapenko A *et al* (2021) Highly accurate protein structure prediction with AlphaFold. *Nature*

Nowak U, Matthews AJ, Zheng S, Chaudhuri J (2011) The splicing regulator PTBP2 interacts with the cytidine deaminase AID and promotes binding of AID to switch-region DNA. *Nat Immunol* 12: 160-166

Qiao Q, Wang L, Meng FL, Hwang JK, Alt FW, Wu H (2017) AID Recognizes Structured DNA for Class Switch Recombination. *Mol Cell* 67: 361-373 e364

Sabari BR, Dall'Agnese A, Boija A, Klein IA, Coffey EL, Shrinivas K, Abraham BJ, Hannett NM, Zamudio AV, Manteiga JC *et al* (2018) Coactivator condensation at super-enhancers links phase separation and gene control. *Science* 361

Ta VT, Nagaoka H, Catalan N, Durandy A, Fischer A, Imai K, Nonoyama S, Tashiro J, Ikegawa M, Ito S *et al* (2003) AID mutant analyses indicate requirement for class-switch-specific cofactors. *Nat Immunol* 4: 843-848

Wang Q, Kieffer-Kwon KR, Oliveira TY, Mayer CT, Yao K, Pai J, Cao Z, Dose M, Casellas R, Jankovic M *et al* (2017) The cell cycle restricts activation-induced cytidine deaminase activity to early G1. *J Exp Med* 214: 49-58

Yin J, Liu M, Liu Y, Wu J, Gan T, Zhang W, Li Y, Zhou Y, Hu J (2019) Optimizing genome editing strategy by primer-extension-mediated sequencing. *Cell Discov* 5: 18

Zahn A, Eranki AK, Patenaude AM, Methot SP, Fifield H, Cortizas EM, Foster P, Imai K, Durandy A, Larijani M *et al* (2014) Activation induced deaminase C-terminal domain links DNA

breaks to end protection and repair during class switch recombination. *Proc Natl Acad Sci U S A* 111: E988-997

Thank you for submitting a new version of your manuscript EMBOJ-2021-109324R-Q, "C-terminal deletion-induced condensation sequesters AID from IgH targets in immunodeficiency." The original referees 1 and 3 have now assessed it once more, with their comments copied below for your information.

As you will see, both referees consider the study substantially improved and are in principle supportive of its publication. However, they both retain some significant reservations regarding the conclusions/interpretation, which may well affect the model currently favored in both title and abstract.

Before taking a final decision here, I would in this case be interested in hearing your responses to these concerns. Therefore, please carefully consider the points raised by the referees, and draft a detailed response clarifying these remaining issues, and suggesting ways how they might be experimentally or textually addressed at this stage. After considering your response (and possible consulting about it also with the referees), we might then discuss all further proceedings with the manuscript.

REFEREE REPORTS

Referee #1 (Report for Author)

The authors have successfully addressed my critiques. One question on the new data in "figure 2 to referee": as revealed by FRAP, there is no mobility difference between the dilute and condense phase in both AIDR190 and OptoAID. This is unusual for LLPS: molecules in the condensed phase usually displayed a much lower mobility. Can the author provide an explanation? The data also do not support the trapping model proposed by the authors in which AID is trapped in the condensed phase formed by AID-delta-C. Is the trapping model still valid or it needs a modification?

Referee #3 (Report for Author)

This is a revised manuscript that studies the distribution of the AID C-terminal deletion in the cell and attempts to understand the dominant negative phenotype of this mutation. The manuscript is much improved and is now quite readable. The authors have made a persuasive case that the faulty distribution of the AID C-terminal deleted mutation in the nucleus leads to a form of aggregation. The authors have responded to my critique. The only remaining concern is that they use the concept of "condensate" to describe mutant AID behavior. To date, transcriptional condensates have been described to concentrate transcriptional machinery to localized positions in the nucleus that then facilitate regulated transcription. The issue is that transcriptional condensates are functional and the "condensates" attributed to AID mutants are dysfunctional. It would be much better to soften this description to indicate that AID mutants appear to concentrate and perhaps aggregate inappropriately. We do not know if there are various types of condensates and what this means biochemically and functionally. It seems to this reader that the inappropriate nuclear distribution of mutant AID is a profoundly nonfunctional. However, we do not know if faulty AID distribution occurs via phase separation per se.

Many thanks for your efficient handling of the manuscript and also for giving us an extra chance to address the remaining concerns. We are also grateful that both referees are supportive of the publication. We strongly believe that concerns can be fully addressed by further clarification of the result/terminology and a new piece of preliminary data.

Regarding the concern from Referee 1, we re-analyzed the presented FRAP data on recovery kinetics from the raw data with more time points and found that our data fit her/his expectation. We previously presented the FRAP data with 20-second intervals in total 2 minutes, which experiment data were actually collected at 5-s intervals. As correctly pointed out by Referee 1, the 20-s interval data failed to show such an evident difference. With the raw data, we calculated the recovery kinetics with a published method (Koulouras et al, 2018), and found that AID-delC within condensed phase does have lower mobility (t1/2-dilute = 2.6 s, t1/2-condensed = 5.6 s; p = 0.027, two-tailed paired t-test). Therefore, the data are supportive of the co-condensation model. Since the AID protein level is sensitive for its physiological function (Takizawa et al., JEM 2008), the collective in vitro and in vivo data strongly support the condensation model. We have attached a re-calculated figure for your reference. We are sorry for the previous vague description and hope the re-calculation can clarify this specific issue.

We thank Referee 3 for the kind suggestion on the terminology/concept of condensation and discuss our thoughts below.

The term "condensation" has been described in a range of distinct physiological processes beyond transcription, including DNA repair, cytoskeletal network, chromatin organization, autophagic degradation, synaptic signaling, etc (Fujioka et al, 2020; Kilic et al, 2019; Sanulli et al, 2019; Woodruff et al, 2017; Wu et al, 2019; Zeng et al, 2018). In addition, the emerging concept of dysfunctional/pathological condensation has been described, e.g. cancer-associated SHP2 mutants (Zhu et al, 2020). Although protein aggregation and condensation both involve high-order assemblies with indeterminate stoichiometry and the terms "condensation" and "aggregation" are loosely used in the literature, there are also many important differences between them. The term "aggregation" describes a non-native state of misfolded protein assembled by irreversible intermolecular interactions. While the term "condensation" refers to dynamic and reversible assemblies of molecules, in which the molecules are functional, mobile, and exchange with the dilute phase (Alberti & Hyman, 2021).

In the main figures, we have shown that AID-delC did not form an inactive complex in vitro (Fig 6A) and in vivo (Fig. 1&6B), and clearly showed the shape difference between AID aggregation and condensation in Fig 5A&B. Here, we also present another piece of preliminary data that shows AID-delC in the condensed phase is also catalytically active in vitro. We tested AID-delC catalytic activity in the condensed phase by adding a DNA substrate that did not affect the protein condensation and found AID-delC exhibited robust deamination activity in the condensed phase. In addition, the "aggregation" term is often referred to as the denatured protein in the AID literature. In a quick survey, we found the description of denatured AID protein as "aggregation" from papers published by 11 labs in the years from 2004 to 2019. For example, papers from the Mani Larijani, Alberto Martin, Hao Wu, Fred Alt, Myron Goodman, Rahul Kohli, Ashok Bhagwat, Kefei Yu, Michael Lieber, Cristina Rada, and Michael Neuberger labs (Bhagwat, 2004; Bransteitter et al, 2004; Gajula et al, 2014; Larijani et al, 2007; Qiao et al, 2017; Uchimura et al, 2011; Yu & Lieber,

2019). In these regards, we believe that the term "condensation" is more suitable to describe the dynamic and catalytic-active assemblies of AID-delC protein.

We think the multiple lines of evidence strongly suggest a condensation model. We also agree with this referee that "the various types of condensates need further biochemical and functional characterization". In this context, we will revise the discussion to fit these comments in the manuscript.

In summary, we hope you will find that the remaining concerns came from either unclear elaboration of the data or loosely-used terminology, and our data are consistent with the proposed model. We have attached the abovementioned figures and references as a PDF file for your reference. Since the figures were previously shown to Referee only, we suggest not to present them in the manuscript to avoid any confusion to the general audience as Referee 3 previously suggested, unless you think it is necessary. In this context, we will further discuss the terminology, our lines of evidence to support the condensation model, and potential alternatives in the revised manuscript. We hope it can help you to make an acceptance decision.

Figures:

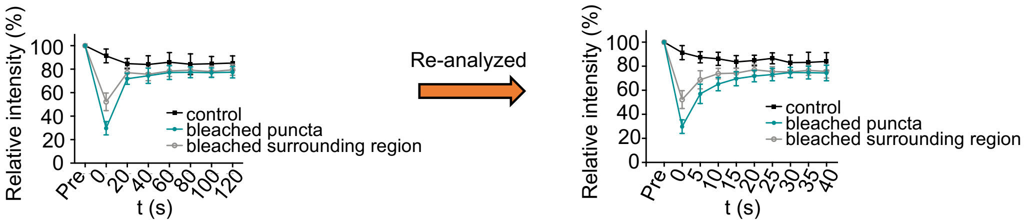

Fig. R1: Characterization of nuclear AID^{R190X} in condensates. Comparison of recovery kinetics between condensed phase and dilute phase with FRAP experiment performed in *Aicda*$^{-/-}$ CH12F3 cells with AID^{R190X}-GFP expression. Left panel: Intensity recovery lines with 20-second intervals. Right panel: Reanalyzed intensity recovery lines with 5-second interval. Both panels were analyzed with a same set of FRAP data. Bleaching occurs at t = 0 s. The background-subtracted fluorescence intensities are normalized by pre-bleach values. Mean with SD of 8 pairs of AID^{R190X} condensates and surrounding regions are shown.

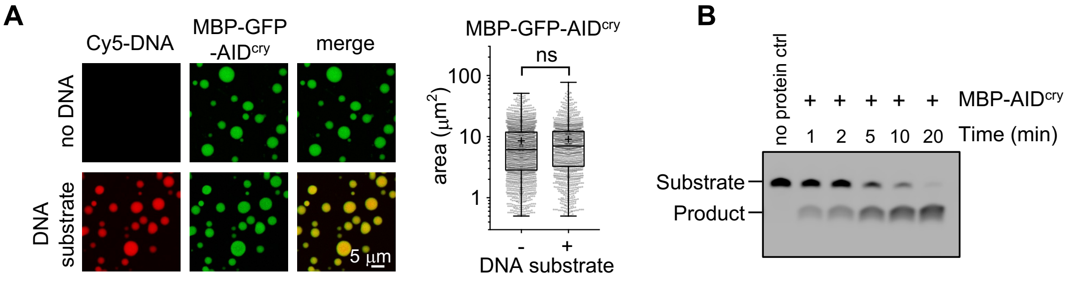

Fig. R2: *In vitro* deamination activity of MBP-AID^{cry} condensates. (**A**) Left: Representative images showing *in vitro* MBP-GFP-AID^{cry} condensates in the presence of a fluorescence-labeled DNA substrate. Right: Sizes of MBP-GFP-AID^{cry} protein condensates in the absence/presence of the DNA substrate are plotted as box plots with all points listed and mean indicated by "+". Unpaired two-tailed Student's *t*-test was performed. ns, $p>0.05$. (**B**) Representative urea gel for visualization of the DNA substrate and product in the deamination reaction. The reactions were performed with 5 μM MBP-AID^{cry} condensates and 50 μM DNA substrates.

References:

Alberti S, Hyman AA (2021) Biomolecular condensates at the nexus of cellular stress, protein aggregation disease and ageing. *Nat Rev Mol Cell Biol* 22: 196-213

Bhagwat AS (2004) DNA-cytosine deaminases: from antibody maturation to antiviral defense. *DNA Repair (Amst)* 3: 85-89

Bransteitter R, Pham P, Calabrese P, Goodman MF (2004) Biochemical analysis of hypermutational targeting by wild type and mutant activation-induced cytidine deaminase. *J Biol Chem* 279: 51612-51621

Fujioka Y, Alam JM, Noshiro D, Mouri K, Ando T, Okada Y, May AI, Knorr RL, Suzuki K, Ohsumi Y *et al* (2020) Phase separation organizes the site of autophagosome formation. *Nature* 578: 301-305

Gajula KS, Huwe PJ, Mo CY, Crawford DJ, Stivers JT, Radhakrishnan R, Kohli RM (2014) High-throughput mutagenesis reveals functional determinants for DNA targeting by activation-induced deaminase. *Nucleic Acids Res* 42: 9964-9975

Kilic S, Lezaja A, Gatti M, Bianco E, Michelena J, Imhof R, Altmeyer M (2019) Phase separation of 53BP1 determines liquid-like behavior of DNA repair compartments. *EMBO J* 38: e101379

Koulouras G, Panagopoulos A, Rapsomaniki MA, Giakoumakis NN, Taraviras S, Lygerou Z (2018) EasyFRAP-web: a web-based tool for the analysis of fluorescence recovery after photobleaching data. *Nucleic Acids Res* 46: W467-W472

Larijani M, Petrov AP, Kolenchenko O, Berru M, Krylov SN, Martin A (2007) AID associates with single-stranded DNA with high affinity and a long complex half-life in a sequence-independent manner. *Mol Cell Biol* 27: 20-30

Qiao Q, Wang L, Meng FL, Hwang JK, Alt FW, Wu H (2017) AID Recognizes Structured DNA for Class Switch Recombination. *Mol Cell* 67: 361-373 e364

Sanulli S, Trnka MJ, Dharmarajan V, Tibble RW, Pascal BD, Burlingame AL, Griffin PR, Gross JD, Narlikar GJ (2019) HP1 reshapes nucleosome core to promote phase separation of heterochromatin. *Nature* 575: 390-394

Takizawa M, Tolarova H, Li Z, Dubois W, Lim S, Callen E, Franco S, Mosaico M, Feigenbaum L, Alt FW *et al* (2008) AID expression levels determine the extent of cMyc oncogenic translocations and the incidence of B cell tumor development. *J Exp Med* 205: 1949-1957

Uchimura Y, Barton LF, Rada C, Neuberger MS (2011) REG-gamma associates with and modulates the abundance of nuclear activation-induced deaminase. *J Exp Med* 208: 2385-2391

Woodruff JB, Ferreira Gomes B, Widlund PO, Mahamid J, Honigmann A, Hyman AA (2017) The Centrosome Is a Selective Condensate that Nucleates Microtubules by Concentrating Tubulin. *Cell* 169: 1066-1077 e1010

Wu X, Cai Q, Shen Z, Chen X, Zeng M, Du S, Zhang M (2019) RIM and RIM-BP Form Presynaptic Active-Zone-like Condensates via Phase Separation. *Mol Cell* 73: 971-984 e975

Yu K, Lieber MR (2019) Current insights into the mechanism of mammalian immunoglobulin class switch recombination. *Crit Rev Biochem Mol Biol* 54: 333-351

Zeng M, Chen X, Guan D, Xu J, Wu H, Tong P, Zhang M (2018) Reconstituted Postsynaptic Density as a Molecular Platform for Understanding Synapse Formation and Plasticity. *Cell* 174: 1172-1187 e1116

Zhu G, Xie J, Kong W, Xie J, Li Y, Du L, Zheng Q, Sun L, Guan M, Li H *et al* (2020) Phase Separation of Disease-Associated SHP2 Mutants Underlies MAPK Hyperactivation. *Cell* 183: 490-502 e418

1st Revision - Editorial Decision 1st Apr 2022

Thank you for your detailed response to the second round of comments by referees 1 and 3, which I have now been able to consider and also to get further feedback on from referee 1. I am happy to say that in light of these clarifications and additional data, we shall be able to accept the manuscript for EMBO Journal publication, following incorporation of the modifications proposed in your tentative response letter, and some additional discussion of the fact that the possibility cannot be excluded that AID mutants are recruited to nucleolus also (or alternatively) through a simple protein-protein interaction (something referee 1 raised in our follow-up discussion). Regarding the previous and newly provided referee-only figures, I agree that they do not need to be incorporated into the manuscript itself, but feel it would be good to retain them as part of the transparent point-by-point response published by in accordance with EMBO Journal's transparent review process.

In addition, please also address the following editorial issues required for EMBO Journal revisions at this stage:

#EMBOJ-2021-109324R1

Response to Referee Comments.

Referee #1 (Report for Author)

The authors have successfully addressed my critiques. One question on the new data in "figure 2 to referee": as revealed by FRAP, there is no mobility difference between the dilute and condense phase in both AIDR190 and OptoAID. This is unusual for LLPS: molecules in the condensed phase usually displayed a much lower mobility. Can the author provide an explanation? The data also do not support the trapping model proposed by the authors in which AID is trapped in the condensed phase formed by AID-delta-C. Is the trapping model still valid or it needs a modification?

Response: We thank the referee for the appreciation of our efforts to address her/his concerns. To address the new specific comment, we re-analyzed the presented FRAP data on recovery kinetics from the raw data with more time points and found that our data fit her/his expectation. We previously presented the FRAP data with 20-second intervals in total 2 minutes, which experiment data were actually collected at 5-s intervals. As correctly pointed out by Referee 1, the 20-s interval data failed to show such an evident difference. With the raw data, we calculated the recovery kinetics with a published method (Koulouras *et al*, 2018), and found that AID-delC within condensed phase does have lower mobility (t1/2-dilute = 2.6 s, t1/2-condensed = 5.6 s; $p = 0.027$, two-tailed paired t-test).

Therefore, the data are supportive of the co-condensation model. Since the AID protein level is sensitive for its physiological function (Takizawa *et al*, 2008), the collective *in vitro* and *in vivo* data strongly support the condensation model. We have attached a re-calculated figure for your reference (Fig. R1). We are sorry for the previous vague description and hope the re-calculation can clarify this specific issue. In the revised manuscript, we further discussed the co-condensation model.

Referee #3 (Report for Author)

This is a revised manuscript that studies the distribution of the AID C-terminal deletion in the cell and attempts to understand the dominant negative phenotype of this mutation. The manuscript is much improved and is now quite readable. The authors have made a persuasive case that the faulty distribution of the AID C-terminal deleted mutation in the nucleus leads to a form of aggregation. The authors have responded to my critique. The only remaining concern is that they use the concept of "condensate" to describe mutant AID behavior. To date,

transcriptional condensates have been described to concentrate transcriptional machinery to localized positions in the nucleus that then facilitate regulated transcription. The issue is that transcriptional condensates are functional and the "condensates" attributed to AID mutants are dysfunctional. It would be much better to soften this description to indicate that AID mutants appear to concentrate and perhaps aggregate inappropriately. We do not know if there are various types of condensates and what this means biochemically and functionally. It seems to this reader that the inappropriate nuclear distribution of mutant AID is a profoundly nonfunctional. However, we do not know if faulty AID distribution occurs via phase separation per se.

Response: We thank referee for the kind suggestion on the terminology/concept of condensation and discuss our thoughts below.

The term "condensation" has been described in a range of distinct physiological processes beyond transcription, including DNA repair, cytoskeletal network, chromatin organization, autophagic degradation, synaptic signaling, etc (Fujioka *et al*, 2020; Kilic *et al*, 2019; Sanulli *et al*, 2019; Woodruff *et al*, 2017; Wu *et al*, 2019; Zeng *et al*, 2018). In addition, the emerging concept of dysfunctional/pathological condensation has been described, e.g. cancer-associated SHP2 mutants (Zhu *et al*, 2020). Although protein aggregation and condensation both involve high-order assemblies with indeterminate stoichiometry and the terms "condensation" and "aggregation" are loosely used in the literature, there are also many important differences between them. The term "aggregation" describes a non-native state of misfolded protein assembled by irreversible intermolecular interactions. While the term "condensation" refers to dynamic and reversible assemblies of molecules, in which the molecules are functional, mobile, and exchange with the dilute phase (Alberti & Hyman, 2021).

In the main figures, we have shown that AID-delC did not form an inactive complex in vitro (Fig 6A) and in vivo (Fig. 1&6B), and clearly showed the shape difference between AID aggregation and condensation in Fig 5A&B. Here, we also present another piece of preliminary data that shows AID-delC in the condensed phase is also **catalytically active *in vitro***. We tested AID-delC catalytic activity in the condensed phase by adding a DNA substrate that did not affect the protein condensation and found AID-delC exhibited robust deamination activity in the condensed phase (Fig. R2). In addition, the "aggregation" term is often referred to as the denatured protein in the AID literature. In a quick survey, we found the description of denatured AID protein as "aggregation" from papers published by 11 labs in the years from 2004 to 2019. For example, papers from the Mani Larijani, Alberto Martin, Hao Wu, Fred Alt, Myron Goodman, Rahul Kohli, Ashok Bhagwat, Kefei Yu, Michael Lieber, Cristina Rada, and Michael Neuberger labs (Bhagwat, 2004; Bransteitter *et al*, 2004; Gajula *et al*, 2014; Larijani *et al*, 2007; Qiao *et al*, 2017; Uchimura *et al*, 2011; Yu & Lieber, 2019). In these regards, we believe that the term "condensation" is more suitable to describe the dynamic and catalytic-active assemblies of AID-delC protein.

We think the multiple lines of evidence strongly suggest a condensation model. We also agree with this referee that "*the various types of condensates need further biochemical and functional characterization*". In this context, we discussed the terminology and the condensation model in the revised manuscript.

Figures:

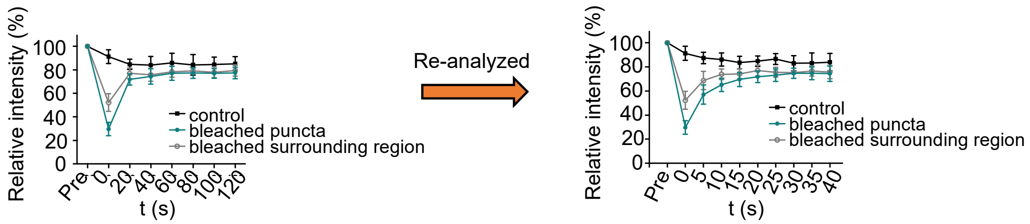

Fig. R1: Characterization of nuclear AID^{R190X} in condensates. Comparison of recovery kinetics between condensed phase and dilute phase with FRAP experiment performed in *Aicda*$^{-/-}$ CH12F3 cells with AID^{R190X}-GFP expression. Left panel: Intensity recovery lines with 20-second intervals. Right panel: Reanalyzed intensity recovery lines with 5-second interval. Both panels were analyzed with a same set of FRAP data. Bleaching occurs at t = 0 s. The background-subtracted fluorescence intensities are normalized by pre-bleach values. Mean with SD of 8 pairs of AID^{R190X} condensates and surrounding regions are shown.

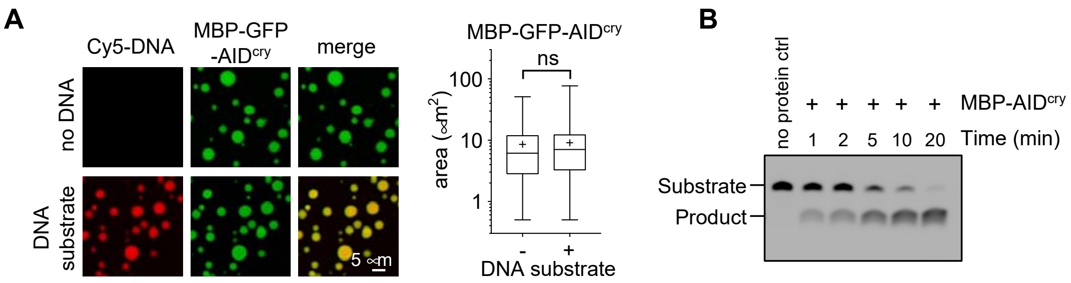

Fig. R2: *In vitro* deamination activity of MBP-AID^{cry} condensates. (**A**) Left: Representative images showing *in vitro* MBP-GFP-AID^{cry} condensates in the presence of a fluorescence-labeled DNA substrate. Right: Sizes of MBP-GFP-AID^{cry} protein condensates in the absence/presence of the DNA substrate are plotted as box plots with all points listed and mean indicated by "+". Unpaired two-tailed Student's *t*-test was performed. ns, $p>0.05$. (**B**) Representative urea gel for visualization of the DNA substrate and product in the deamination reaction. The reactions were performed with 5 µM MBP-AID^{cry} condensates and 50 µM DNA substrates.

References:

Alberti S, Hyman AA (2021) Biomolecular condensates at the nexus of cellular stress, protein aggregation disease and ageing. *Nat Rev Mol Cell Biol* 22: 196-213

Bhagwat AS (2004) DNA-cytosine deaminases: from antibody maturation to antiviral defense. *DNA Repair (Amst)* 3: 85-89

Bransteitter R, Pham P, Calabrese P, Goodman MF (2004) Biochemical analysis of hypermutational targeting by wild type and mutant activation-induced cytidine deaminase. *J Biol Chem* 279: 51612-51621

Fujioka Y, Alam JM, Noshiro D, Mouri K, Ando T, Okada Y, May AI, Knorr RL, Suzuki K, Ohsumi Y *et al* (2020) Phase separation organizes the site of autophagosome formation. *Nature* 578: 301-305

Gajula KS, Huwe PJ, Mo CY, Crawford DJ, Stivers JT, Radhakrishnan R, Kohli RM (2014) High-throughput mutagenesis reveals functional determinants for DNA targeting by activation-induced deaminase. *Nucleic Acids Res* 42: 9964-9975

Kilic S, Lezaja A, Gatti M, Bianco E, Michelena J, Imhof R, Altmeyer M (2019) Phase separation of 53BP1 determines liquid-like behavior of DNA repair compartments. *EMBO J* 38: e101379

Koulouras G, Panagopoulos A, Rapsomaniki MA, Giakoumakis NN, Taraviras S, Lygerou Z (2018) EasyFRAP-web: a web-based tool for the analysis of fluorescence recovery after photobleaching data. *Nucleic Acids Res* 46: W467-W472

Larijani M, Petrov AP, Kolenchenko O, Berru M, Krylov SN, Martin A (2007) AID associates with single-stranded DNA with high affinity and a long complex half-life in a sequence-independent manner. *Mol Cell Biol* 27: 20-30

Qiao Q, Wang L, Meng FL, Hwang JK, Alt FW, Wu H (2017) AID Recognizes Structured DNA for Class Switch Recombination. *Mol Cell* 67: 361-373 e364

Sanulli S, Trnka MJ, Dharmarajan V, Tibble RW, Pascal BD, Burlingame AL, Griffin PR, Gross JD, Narlikar GJ (2019) HP1 reshapes nucleosome core to promote phase separation of heterochromatin. *Nature* 575: 390-394

Takizawa M, Tolarova H, Li Z, Dubois W, Lim S, Callen E, Franco S, Mosaico M, Feigenbaum L, Alt FW *et al* (2008) AID expression levels determine the extent of cMyc oncogenic translocations and the incidence of B cell tumor development. *J Exp Med* 205: 1949-1957

Uchimura Y, Barton LF, Rada C, Neuberger MS (2011) REG-gamma associates with and modulates the abundance of nuclear activation-induced deaminase. *J Exp Med* 208: 2385-2391

Woodruff JB, Ferreira Gomes B, Widlund PO, Mahamid J, Honigmann A, Hyman AA (2017) The Centrosome Is a Selective Condensate that Nucleates Microtubules by Concentrating Tubulin. *Cell* 169: 1066-1077 e1010

Wu X, Cai Q, Shen Z, Chen X, Zeng M, Du S, Zhang M (2019) RIM and RIM-BP Form Presynaptic Active-Zone-like Condensates via Phase Separation. *Mol Cell* 73: 971-984 e975

Yu K, Lieber MR (2019) Current insights into the mechanism of mammalian immunoglobulin class switch recombination. *Crit Rev Biochem Mol Biol* 54: 333-351

Zeng M, Chen X, Guan D, Xu J, Wu H, Tong P, Zhang M (2018) Reconstituted Postsynaptic Density as a Molecular Platform for Understanding Synapse Formation and Plasticity. *Cell* 174: 1172-1187 e1116

Zhu G, Xie J, Kong W, Xie J, Li Y, Du L, Zheng Q, Sun L, Guan M, Li H *et al* (2020) Phase Separation of Disease-Associated SHP2 Mutants Underlies MAPK Hyperactivation. *Cell* 183: 490-502 e418

2nd Revision - Editorial Decision 8th Apr 2022

Thank you for submitting your final revised manuscript for our consideration. I am pleased to inform you that we have now accepted it for publication in The EMBO Journal.

EMBO Press Author Checklist

Corresponding Author Name: Fei-Long Meng, Jiazhi Hu	
Journal Submitted to: The EMBO Journal	
Manuscript Number: EMBOJ-2021-109324	

USEFUL LINKS FOR COMPLETING THIS FORM
[The EMBO Journal - Author Guidelines]
[EMBO Reports - Author Guidelines]
[Molecular Systems Biology - Author Guidelines]
[EMBO Molecular Medicine - Author Guidelines]

Reporting Checklist for Life Science Articles (updated January 2022)
This checklist is adapted from Materials Design Analysis Reporting (MDAR) Checklist for Authors. MDAR establishes a minimum set of requirements in transparent reporting in the life sciences (see Statement of Task: 10.31222/osf.io/9sm4x). Please follow the journal's guidelines in preparing your manuscript.
Please note that a copy of this checklist will be published alongside your article.

Abridged guidelines for figures
1. Data
The data shown in figures should satisfy the following conditions:
- the data were obtained and processed according to the field's best practice and are presented to reflect the results of the experiments in an accurate and unbiased manner.
- ideally, figure panels should include only measurements that are directly comparable to each other and obtained with the same assay.
- plots include clearly labeled error bars for independent experiments and sample sizes. Unless justified, error bars should not be shown for technical replicates.
- if n<5, the individual data points from each experiment should be plotted. Any statistical test employed should be justified.
- Source Data should be included to report the data underlying figures according to the guidelines set out in the authorship guidelines on Data Presentation.

2. Captions
Each figure caption should contain the following information, for each panel where they are relevant:
- a specification of the experimental system investigated (eg cell line, species name).
- the assay(s) and method(s) used to carry out the reported observations and measurements.
- an explicit mention of the biological and chemical entity(ies) that are being measured.
- an explicit mention of the biological and chemical entity(ies) that are altered/varied/perturbed in a controlled manner.
- the exact sample size (n) for each experimental group/condition, given as a number, not a range;
- a description of the sample collection allowing the reader to understand whether the samples represent technical or biological replicates (including how many animals, litters, cultures, etc.).
- a statement of how many times the experiment shown was independently replicated in the laboratory.
- definitions of statistical methods and measures:
  - common tests, such as t-test (please specify whether paired vs. unpaired), simple χ2 tests, Wilcoxon and Mann-Whitney tests, can be unambiguously identified by name only, but more complex techniques should be described in the methods section;
  - are tests one-sided or two-sided?
  - are there adjustments for multiple comparisons?
  - exact statistical test results, e.g., P values = x but not P values < x;
  - definition of 'center values' as median or average;
  - definition of error bars as s.d. or s.e.m.

Please complete ALL of the questions below.
Select "Not Applicable" only when the requested information is not relevant for your study.

Materials

Newly Created Materials	Information included in the manuscript?	In which section is the information available? (Reagents and Tools Table, Materials and Methods, Figures, Data Availability Section)
New materials and reagents need to be available; do any restrictions apply?	Yes	Materials and Methods

Antibodies	Information included in the manuscript?	In which section is the information available? (Reagents and Tools Table, Materials and Methods, Figures, Data Availability Section)
For **antibodies** provide the following information:<br>- Commercial antibodies: RRID (if possible) or supplier name, catalogue number and or/clone number<br>- Non-commercial: RRID or citation	Yes	Materials and Methods

DNA and RNA sequences	Information included in the manuscript?	In which section is the information available? (Reagents and Tools Table, Materials and Methods, Figures, Data Availability Section)
**Short novel DNA or RNA including primers, probes**: provide the sequences.	Yes	Materials and Methods, Appendix Table S2

Cell materials	Information included in the manuscript?	In which section is the information available? (Reagents and Tools Table, Materials and Methods, Figures, Data Availability Section)
**Cell lines:** Provide species information, strain. Provide accession number in repository **OR** supplier name, catalog number, clone number, and/**OR** RRID.	Yes	Materials and Methods
**Primary cultures:** Provide species, strain, sex of origin, genetic modification status.	Yes	Materials and Methods
Report if the cell lines were recently **authenticated** (e.g., by STR profiling) and tested for mycoplasma contamination.	Yes	Materials and Methods

Experimental animals	Information included in the manuscript?	In which section is the information available? (Reagents and Tools Table, Materials and Methods, Figures, Data Availability Section)
**Laboratory animals or Model organisms:** Provide species, strain, sex, age, genetic modification status. Provide accession number in repository **OR** supplier name, catalog number, clone number, **OR** RRID.	Yes	Materials and Methods
**Animal observed in or captured from the field:** Provide species, sex, and age where possible.	Not Applicable	
Please detail **housing and husbandry conditions**.	Yes	Materials and Methods

Plants and microbes	Information included in the manuscript?	In which section is the information available? (Reagents and Tools Table, Materials and Methods, Figures, Data Availability Section)
**Plants:** provide species and strain, ecotype and cultivar where relevant, unique accession number if available, and source (including location for collected wild specimens).	Not Applicable	
**Microbes:** provide species and strain, unique accession number if available, and source.	Yes	Materials and Methods

Human research participants	Information included in the manuscript?	In which section is the information available? (Reagents and Tools Table, Materials and Methods, Figures, Data Availability Section)
If collected and within the bounds of privacy constraints report on age, sex and gender or ethnicity for all study participants.	Not Applicable	

Core facilities	Information included in the manuscript?	In which section is the information available? (Reagents and Tools Table, Materials and Methods, Figures, Data Availability Section)
If your work benefited from core facilities, was their service mentioned in the acknowledgments section?	Yes	Acknowledgments

Design

Study protocol	Information included in the manuscript?	In which section is the information available? (Reagents and Tools Table, Materials and Methods, Figures, Data Availability Section)
If study protocol has been **pre-registered, provide DOI in the manuscript**. For clinical trials, provide the trial registration number **OR** cite DOI.	Not Applicable	
Report the **clinical trial registration number** (at ClinicalTrials.gov or equivalent), where applicable.	Not Applicable	

Laboratory protocol	Information included in the manuscript?	In which section is the information available? (Reagents and Tools Table, Materials and Methods, Figures, Data Availability Section)
Provide DOI OR other citation details if **external detailed step-by-step protocols** are available.	Not Applicable	

Experimental study design and statistics	Information included in the manuscript?	In which section is the information available? (Reagents and Tools Table, Materials and Methods, Figures, Data Availability Section)

Include a statement about **sample size** estimate even if no statistical methods were used.	Yes	Materials and Methods
Were any steps taken to minimize the effects of subjective bias when allocating animals/samples to treatment (e.g. **randomization procedure**)? If yes, have they been described?	Not Applicable	
Include a statement about **blinding** even if no blinding was done.	Not Applicable	
Describe **inclusion/exclusion criteria** if samples or animals were excluded from the analysis. Were the criteria pre-established?<br><br>If sample or data points were omitted from analysis, report if this was due to attrition or intentional exclusion and provide justification.	Not Applicable	
For every figure, are **statistical tests** justified as appropriate? Do the data meet the assumptions of the tests (e.g., normal distribution)? Describe any methods used to assess it. Is there an estimate of variation within each group of data? Is the variance similar between the groups that are being statistically compared?	Yes	Materials and Methods, Figure legends

Sample definition and in-laboratory replication	Information included in the manuscript?	In which section is the information available?<br>(Reagents and Tools Table, Materials and Methods, Figures, Data Availability Section)
In the figure legends: state number of times the experiment was **replicated** in laboratory.	Yes	Figure legends
In the figure legends: define whether data describe **technical or biological replicates**.	Yes	Figure legends

Ethics

Ethics	Information included in the manuscript?	In which section is the information available?<br>(Reagents and Tools Table, Materials and Methods, Figures, Data Availability Section)
Studies involving **human participants**: State details of **authority granting ethics approval** (IRB or equivalent committee(s), provide reference number for approval.	Not Applicable	
Studies involving **human participants**: Include a statement confirming that **informed consent** was obtained from all subjects and that the experiments conformed to the principles set out in the WMA Declaration of Helsinki and the Department of Health and Human Services Belmont Report.	Not Applicable	
Studies involving **human participants:** For publication of **patient photos**, include a statement confirming that consent to publish was obtained.	Not Applicable	
Studies involving experimental **animals**: State details of **authority granting ethics approval** (IRB or equivalent committee(s), provide reference number for approval. Include a statement of compliance with ethical regulations.	Yes	Materials and Methods
Studies involving **specimen and field samples:** State if relevant **permits** obtained, provide details of authority approving study; if none were required, explain why.	Not Applicable	

Dual Use Research of Concern (DURC)	Information included in the manuscript?	In which section is the information available?<br>(Reagents and Tools Table, Materials and Methods, Figures, Data Availability Section)
Could your study fall under dual use research restrictions? Please check biosecurity documents and list of **select agents and toxins** (CDC): https://www.selectagents.gov/sat/list.htm	Not Applicable	
If you used a select agent, is the security level of the lab appropriate and reported in the manuscript?	Not Applicable	
If a study is subject to dual use research of concern regulations, is the name of the **authority granting approval and reference number** for the regulatory approval provided in the manuscript?	Not Applicable	

Reporting

The MDAR framework recommends adoption of discipline-specific guidelines, established and endorsed through community initiatives. Journals have their own policy about requiring specific guidelines and recommendations to complement MDAR.

Adherence to community standards	Information included in the manuscript?	In which section is the information available?<br>(Reagents and Tools Table, Materials and Methods, Figures, Data Availability Section)
State if relevant guidelines or checklists (e.g., **ICMJE, MIBBI, ARRIVE, PRISMA**) have been followed or provided.	Not Applicable	
For **tumor marker prognostic studies,** we recommend that you follow the **REMARK** reporting guidelines (see link list at top right). See author guidelines, under 'Reporting Guidelines'. Please confirm you have followed these guidelines.	Not Applicable	
For **phase II and III randomized controlled trials**, please refer to the **CONSORT** flow diagram (see link list at top right) and submit the CONSORT checklist (see link list at top right) with your submission. See author guidelines, under 'Reporting Guidelines'. Please confirm you have submitted this list.	Not Applicable	

Data Availability

Data availability	Information included in the manuscript?	In which section is the information available?<br>(Reagents and Tools Table, Materials and Methods, Figures, Data Availability Section)
Have **primary datasets** been deposited according to the journal's guidelines (see 'Data Deposition' section) and the respective accession numbers provided in the Data Availability Section?	Yes	Data availability
Were **human clinical and genomic datasets** deposited in a public access-controlled repository in accordance to ethical obligations to the patients and to the applicable consent agreement?	Not Applicable	
Are **computational models** that are central and integral to a study available without restrictions in a machine-readable form? Were the relevant accession numbers or links provided?	Not Applicable	
If publicly available data were reused, provide the respective **data citations in the reference list**.	Yes	Appendix Table S3
